# Pangolin distribution and conservation status in Bangladesh

**DOI:** 10.1371/journal.pone.0175450

**Published:** 2017-04-07

**Authors:** Scott J. Trageser, Animesh Ghose, Muzaffar Faisal, Passing Mro, Poroy Mro, Shahriar Caesar Rahman

**Affiliations:** Creative Conservation Alliance, Dhaka, Bangladesh; University of Tasmania, AUSTRALIA

## Abstract

Asian pangolins are a highly-threatened species group, mainly due to the perceived medicinal value of their scales. Increased demand from China has resulted in pangolins being the most trafficked mammal in the world. Three pangolin species are reported to occur in Bangladesh: *Manis pentadactyla*, *M*. *crassicaudata*, and *M*. *javanica*. No peer-reviewed studies exist detailing these species’ current distribution or status within Bangladesh. A literature review was conducted resulting in the clarification of conflicting reports and misidentified observations and specimen records. In this paper, we also report the current status of pangolins (*Manis* spp.) in Bangladesh based on semi-structured interviews, camera trapping, media queries, and field surveys employing traditional ecological knowledge and non-randomized transect surveys. Ethnozoological knowledge pertaining to the natural history of *M*. *pentadactyla* is also reported from experienced Mro tribal hunters. The critically endangered *M*. *pentadactyla* was verified to occur in northwest, northeast, and southeast Bangladesh in natural and degraded habitats. Interviews with the Mro tribe in the southeast indicate that pangolin populations there were likely extirpated in 2014 due to skilled commercial collection beginning in 2010. Evidence of extant *M*. *crassicaudata* and *M*. *javanica* populations remain unverified and questionable, and historical records of *M*. *crassicaudata* and *M*. *javanica* are likely a result of misidentification.

## Introduction

Bangladesh is rich in biodiversity, yet geographically-explicit knowledge regarding the status and distribution of pangolins, as well as many other species, is limited. Pangolins are a highly-threatened order of mammal whose presence in Bangladesh is debated in the literature. IUCN considers the Sunda pangolin (*Manis javanica*) and Chinese pangolin (*M*. *pentadactyla*) as critically endangered [1,2], while Indian pangolins (*M*. *crassicaudata*) are considered endangered [3]. A review of the literature detailing the distribution of three pangolin species reported to range Bangladesh reveals that the existing body of knowledge for these species is scant, contradictory, and unreliable. This knowledge gap limits the efficacy of pangolin conservation efforts for the greater Indo-Burman region. The following multidisciplinary analysis establishes the presence of *M*. *pentadactyla* in multiple sites within Bangladesh and provides an important baseline for pangolin conservation efforts.

Bangladesh is situated on the western cusp of the Indo-Burma biodiversity hotspot, which begins east of the Ganges-Brahmaputra lowlands [4]. A confluence of diversity is found here as the biogeography of Bangladesh is of both Gondwanan and Laurasian origin [4]. As a result, many wide-ranging species reach their distributional limits in Bangladesh, but currently it is unclear where to demarcate many of these range boundaries. The Ganges-Brahmaputra-Meghna river system is traditionally thought to be a main zoogeographical barrier [4], but misreporting of species identifications across several classes has confounded this assumption [5]. Despite these unique species assemblages, a long history of war and political instability in the country has stifled research activity, leaving many fundamental research questions unanswered. The uncertainty of pangolin species identifications and distributions, along with many other taxa, are also a direct result of a preference for hands-off research in Bangladesh, due to permitting complexities regarding the handling and harassment of wildlife. This results in conclusions being made from brief, superficial assessments or low-quality photographs. Lastly, the lack of accurate natural history literature for the region is also contributing to these specific identity and distributional uncertainties.

The family Manidae is comprised of eight extant species of pangolin distributed discontinuously through tropical and subtropical Asia, as well as Africa [1,2]. These scaled mammals evolved to prey strictly upon ants and termites and are morphologically convergent to the myrmecophagous mammals inhabiting the New World and Australia [1,2]. Asian pangolins, and more recently the African species as well, are highly-threatened and have quickly gained infamy as the most trafficked CITES protected mammal in the world [6]. Over-exploitation for the traditional Chinese medicine trade and for their meat, which is coveted as a status symbol in Vietnamese and Chinese markets, is largely responsible for their sharp decline [1]. Habitat destruction and subsistence hunting contribute to their plight, though to a lesser extent [1,7]. All wildlife, including pangolins, have legal protection from poaching in Bangladesh through the creation of natural reserves by the Wildlife (Conservation & Security) Act of 2012. Of the four species of pangolin that occur in Asia, *M*. *pentadactyla*, *M*. *crassicaudata*, and *M*. *javanica* are reported to have occurred historically in Bangladesh [7–9]. The fourth species, *Manis culionensis*, is endemic to the Philippines [10].

Based on field experience and personal communications with locals, Khan [9] concluded that *M*. *pentadactyla* and *M*. *javanica* possibly occur in Bangladesh, but no sight records or specimens of *M*. *javanica* exist in Bangladesh. The closest recorded *M*. *javanica* to Bangladesh is a specimen from the Kayah state of Myanmar preserved in the Royal Belgian Institute of Natural Sciences (RBINS 355589), approximately 470 km from the southeast border of Bangladesh. With regards to *M*. *pentadactyla*, there is more literature supporting the presence of the species in Bangladesh. Choudrury’s (2004) research experiences led him to assert that the species is common in the northeastern regions of Bangladesh, although he recognizes the lack of sight and specimen records. Bangladesh’s body of literature includes only a single pangolin photo of *M*. *pentadactyla* (S1 File), reported by Khan [8] and photographed in 2007 within the degraded forest of Khagrachari in the northern region of the Chittagong Hill Tracts (M. Khan 2015, pers. comm.). To the best of the authors’ knowledge, Specimen 18 of the Chittagong University, Department of Zoology collection is the only museum record that exists for any pangolin species within Bangladesh. Specimen 18 is a preserved *M*. *pentadactyla*, misidentified as *M*. *crassicaudata* within the museum catalog. The closest record of *M*. *pentadactyla* outside of Bangladesh is a museum specimen acquired 32 km from the northern border, in the Khasi Hills, Meghalya, India: Field Museum of Natural History specimen number FMNH 75880. Currently, the IUCN does not recognize an extant or historic distribution of *M*. *pentadactyla* or *M*. *javanica* in Bangladesh [1,2].

Lacking empirical evidence, Khan [7] states that *M*. *crassicaudata* historically occurred throughout Bangladesh, with the exception of coastal areas, and that the species has been extirpated from several northwest and west-central regions. Based on these assertions, CITES Bangladesh (1986) [11] designated *M*. *crassicaudata* as rare within the country. Sight records exist several years later for *M*. *crassicaudata* in West Bengal from the bordering districts of Koch Bihar, Jalpaiguri, and Nadia [12] in similar habitats and with similar threats to those in northwest and west-central Bangladesh. Choudrury [5] further specifies that *M*. *crassicaudata* is absent east of the Ganges-Brahmaputra-Meghna river system, but IUCN Bangladesh (2003) [13] and Ahmed (2009) [14] directly contradict Choudrury’s claim and state that *M*. *crassicaudata* remains extant only in southeast Bangladesh, east of the Ganges-Brahmaputra-Meghna river system. In addition, Ahsan (2008) [15] has falsely claimed that *M*. *crassicaudata* rarely occurs on the campus of Chittagong University, Chittagong, Bangladesh. Subsequently, the 2008 IUCN assessment claimed *M*. *crassicaudata* occurred in Bangladesh in low numbers [3] based on false information. Photographs provided by Ahsan of these observations reveal that the reported pangolin species is correctly identified as *M*. *pentadactyla* (S2 File). Heath (1995) [16] however claimed that *M*. *crassicaudata* has been extirpated from the country since 1995, which is contradictory to all subsequent reports. Heath’s publication led the IUCN (2014) [3] to declare *M*. *crassicaudata* as “possibly extinct” in Bangladesh.

Current knowledge of the distribution and status of pangolins in Bangladesh is based on unverified, and therefore unreliable assertions written in field guides and published checklists. Locally, many Bangladeshis are aware of the occurrence of pangolins in many protected areas throughout northeast Bangladesh such as Satchari National Park and Rema-Kalenga Wildlife Sanctuary (M. Khan 2015, pers. comm.), but the literature does not recognize which of the three possible pangolin species these observations refer to. No peer-reviewed studies exist assessing the status or distribution of pangolins within Bangladesh. Furthermore, no consensus exists pertaining to the validity of the historical distributions of the three pangolin species within Bangladesh [5,17].

It is clear there exists a substantial knowledge gap concerning the current status, distribution, and natural history of pangolins within Bangladesh. Clarification of this baseline information is required to develop properly informed management decisions at both the national and global level. Therefore, we conducted surveys within our study sites in the Chittagong Hill Tracts and Lawachara National park to accurately investigate which of the three potential pangolin species remain extant within the borders of Bangladesh.

### Study sites

1) Chittagong Hill Tracts (CHT), southeast Bangladesh—Sangu Wildlife Sanctuary is located within the Bandarban district in the southern region of the CHT and encompasses approximately 350 km^2^ of secondary and primary evergreen forest. The CHT shares a 200 km border with Myanmar and is part of the Arakan-Yoma mountain range that is oriented north to south, in parallel ridges, incised by deep gorges and with topography and vegetation very similar to that of western Myanmar [18]. Overall population density in Bangladesh in 2015 was 1236.81 people per square kilometer [19] and the CHT region has the lowest human population density in the country with 120 people per square kilometer of 11 different ethnic groups including the Chakma, Marma, Tripura, and Mro [20]. Until recently, much of the CHT consisted of old-growth, mixed-evergreen, and natural bamboo forests. 58,542 ha of forest (30% or greater forest cover) have been cleared through the use of shifting agriculture practices and illegal logging [21,22] between 2001 and 2014 [23]. Deforestation in the Chittagong District constitutes 85% of the total forest cover loss for Bangladesh between 2001 and 2014 [23]. Patches of old-growth forest remain in the extreme southeast near the Bangladesh-Myanmar border, and in the north near the Bangladesh-India border. Within this agricultural landscape, patches of riparian forest have been preserved by the local tribes to retain water and supply timber products for local household use [21].

2) Lawachara National Park (LNP), northeast Bangladesh—LNP is a 1,250 hectare semi-evergreen forest mosaic located near Sreemangal, Moulvibazaar District in northeast Bangladesh. Since the 1920s, much of the natural forest has been altered by patchwork plantation rotations of *Xylia dolabriformis*, *Lophopetalum fimbriatum*, *Tectona grandis*, *Lagerstroemia speciosa*, *Artocarpus chaplasha*, *Gmelina arborea*, *Neolamarckia cadamba*, and exotics such as *Eucalyptus* spp., and *Acacia* spp.. The forest is highly disturbed with only a small patch of native floral assemblages, the area of which has not been officially quantitated, but is estimated to be less than 100 hectares. This plantation model is typical for most protected forests in Bangladesh, especially in the northeastern regions [24]. Several thousand impoverished tea plantation laborers live in villages next to Lawachara National Park leading to rampant illegal, subsistence logging and poaching within the park, despite the implied protection afforded by its National Park designation.

## Materials and methods

### Chittagong hill tracts

One group of key partners of Creative Conservation Alliance’s hunting mitigation program in the Chittagong Hill Tracts are local hunters that provide traditional ecological knowledge (TEK) services e.g. tracking, trapping site identification, and elucidating historical ecological trends in local forests. In addition, these chosen advocates were trained and subsequently employed as parabiologists–laymen lacking a formal biological background yet trained to carry out specific scientific tasks—to carry out the interviews and camera trap surveys described below. Poroy Mro and Passing Mro, co-authors of this publication, are two of our primary parabiologists.

#### Interviews

To obtain baseline data of regional fauna for a separate study, twenty-eight semi-structured interview surveys were conducted during January 2015 in 28 tribal villages within the Sangu Wildlife Sanctuary and adjacent areas. Each village was visited at least twice during the surveys, and with few exceptions, interviews were conducted at night to ensure all villagers had returned from their daily activities. Interviews were conversational in nature instead of rigid question-answer sessions, following the guidelines of Huntington [25]. All interviews were conducted in the Mro language and subsequently translated into English. Interviewee ages ranged from approximately 16 to 70 and gender ratio was approximately 1:1. Interviewees were asked to recall the number of wild animals each household had harvested within the previous year. Photographs of pangolins were shown to the interviewees, while the reported species’ occurrence, related traditional ecological knowledge, and species-specific taboos were recorded.

To conduct socio-anthropological studies in Bangladesh, permission is not required from any ethical committee and, in addition, no governing body exists with purview applicable to this study; therefore no ethical oversight was obtained. Informed consent was acquired from every survey participant prior to the interview process in verbal form rather than written due to widespread illiteracy in the region. Participants were made aware of the option to decline the interview at any point during the process. No identifying participant or household data were collected.

#### Camera trap survey

Opportunistic camera trap surveys were conducted in the Sangu-Matamuhuri Valley by our parabiologists from May 2015 through February 2016. Ten Bushnel Trophy Cam HD cameras were placed non-randomly on trails known to be frequented by medium-to-large mammals to increase the likelihood of success. No measures to prevent bias were taken as this effort aimed only to document the biodiversity of the area using the most efficient methodology possible. Trap sites occurred in mixed-evergreen forest and swidden agriculture fields and were identified by local parabiologists who have hunted the region for several decades. Camera traps were placed from one kilometer to ten kilometers away from the villages in both primary and secondary forest. This effort was part of an ongoing study to assess the presence and absence of medium-to-large mammals in the area and has resulted in the rediscovery of several large mammals previously thought to be extirpated from Bangladesh [S8 File].

#### Pangolin burrow inspection

A BlueFire WiFi 10M Android Borescope connected to an Android smartphone was used to inspect a single active pangolin burrow in Lawachara National park.

### Lawachara National Park

#### Transect surveys

Non-random, trail-bound transects were performed nocturnally within LNP by groups of 3 or more amateur naturalists, aided by handheld lights or headlamps. Transects averaged approximately 1.5 km in length and focused on sections of replanted mixed-evergreen forest that were at least 50 years of age. Randomized transect sampling was not possible due to the safety restrictions imposed on foreigners by the Bangladesh Forest Department. These restrictions limited survey site selection to five maintained trail systems. Transects occurred over the course of 41 nights, averaging four hours each night, with a broad focus including mammals, herpetofauna, and invertebrates. All general visual encounter surveys were performed during the months of June through August from 2014–2016 during an annual field workshop held during this period.

In June 2015, three of our most experienced parabiologists led intensive searches in LNP seeking sign of *Manis* spp. occurrence for a period of two days from 0900 to 1800 each day. In traditional Mro fashion, the surveys began with each individual tracker following a distinct route for several hours, after which the team would regroup and assess the situation. If recent sign was discovered, one tracker would trace the sign back to its origin while being flanked on either side by the other two trackers. Signs indicating recent pangolin activity included fallen leaves with three claw piercings, tail drag traces, flies and ticks at the entrance of an active burrow, and recently excavated soil. A single-entrance burrow distinguishes a pangolin burrow from that of a Malaysian porcupine (*Hystrix brachyura*), which constructs a dual-entrance burrow, and the larger diameter of adult pangolin burrows (Approx. 15–20 cm) distinguishes them from those of ferret badger (*Melogale* sp.) and hog badger (*Arctonyx* sp.) (Less than 15 cm) (P. Mro pers. Obs.).

#### Outreach

Educational outreach programs have been conducted for the local populace residing near LNP since 2010, largely in association with the CCA’s Burmese python (*Python bivitattus*) management efforts. During these outreach programs, people were urged to contact our organization through a local representative with reports of wildlife sightings including those of pangolins. Group meetings were held with up to 100 people in attendance, focusing on bringing awareness to ecological issues such as animal persecutions, poaching, logging, and water use.

### Media queries

Media reports are often used to understand the status and distribution of threatened species [26]. Queries were performed through Google, Google Scholar, and Web of Knowledge seeking references to any verifiable pangolin reports within Bangladesh. Qualified reports were associated with unique, identifiable pictures and cross-referenced to protect against duplicate entries. These queries were carried out in English and in Bangla by utilizing the common vernacular name “Bon Rui.” Three queried terms were used: Bon rui; pangolin Bangladesh; and Manis Bangladesh. Poaching efforts in Bangladesh have reportedly increased in 2010 (P. Mro 2015, M. Khan 2015, pers. comm.) and as such, only articles published after 2010 were considered during the queries in order to establish an accurate, current distribution.

## Results and discussion

### Chittagong Hill Tracts

As a result of our interview surveys, it was concluded that at least four individual pangolins, likely *M*. *pentadactyla*, were reported to have been killed by Mro hunters in 2015. One of the most experienced Mro hunters reported that he had personally killed 32 pangolins in the area since 2010, but only 2 of those pangolins were killed in 2014, indicating a sharp decline in the regional pangolin population. A consensus existed amongst the interviewed villagers who claim that pangolins were extirpated from most of the southern regions within the Chittagong Hill Tracts by the end of 2014. It is possible that pangolins still occur in isolated pockets deep within the CHT (P. Mro 2015, pers. obs.). Corroborating this claim, our camera trap survey that consisted of 500 effective camera trap nights and 16,618 total photographs, yielded photographs of 19 mammal species, but the presence of pangolins was conspicuously absent.

In stark contrast to the normal subsistence hunting habits of the Mro, pangolins are specifically targeted for the commercial exportation of their scales. The reported price of 1 kg of pangolin scales in the area ranged from USD 250–400 in the summer of 2015. By January, 2016 the maximum price had increased to USD 500 (P. Mro 2016, pers. comm.), a 25% increase in 6 months. This is still lower than the prices reported in neighboring Nepal of USD 500–625 per kg [27]. Comparatively, tiger and leopard bones sell for USD 130 per kilogram and live bear cubs sell for USD 70 (P. Mro 2016, pers. comm.). All wildlife products are sold to traders from either Thanchi or Alikadam, the closest towns to the Mro villages. These products are intended for illegal export into Myanmar, likely destined for consumption in China.

**Fig 1:**
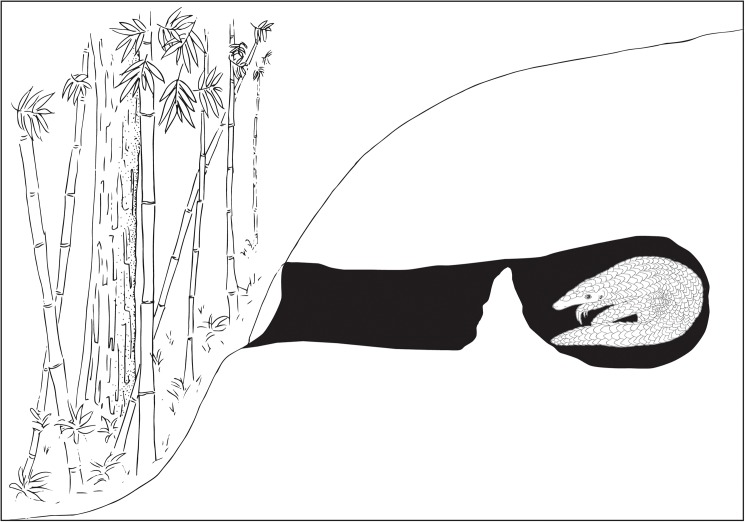
*Manis pentadactyla* burrow. Illustration of *Manis pentadactyla* burrow with barrier. Figure is not drawn to scale.

Interviews with local hunters/parabiologists provided several unreported natural history traits for pangolins with all interviewees reporting that a single foraging pangolin can excavate up to 15 burrows in a single night. Pangolins are known to be prolific burrowers with a reported total number of resident burrows for *M*. *pentadactyla* ranging from 29.4–39.6 and 72.5–83.3 for females and males respectively [28]. Based on this, the estimate of 15 burrows a night based on the Mro’s traditional ecological knowledge seems plausible. During the same study, resident burrows are reported to reach a depth of 201.6±94.8 cm [28], which affirms the Mro hunters’ claims of average resident burrow depth reaching approximately 300cm. The Mro hunters also claim resident burrows occasionally extend upwards of 10m deep, which is twice the reported maximum depth of 505cm [28]. It is also claimed by the hunters that if one is to begin excavating a pangolin from a resident burrow, the pangolin is capable of excavating an escape tunnel at a more rapid pace than the hunters can match, until the opposite surface of the hillside has been breached and the pangolin’s escape is realized. By using a 10m borescope resident burrow design was found to consist of a 30 degree upward sloping tunnel, terminating in a resting chamber at 9.7m in depth. The resting chamber is concealed by a false wall which terminates approximately 3cm prior to reaching the roof of the tunnel (Fig 1). It is not known if this false wall is constructed only upon disturbance, but it likely serves as a predator deterrent. It is known that *M*. *crassicaudata* creates a barrier of loose dirt at the entrance of the burrow to evade predator detection [29], but this behavior has not been reported with *M*. *pentadactyla*.

Two forms of pangolin were reported by Mro hunters to exist within the CHT. One is described as dark and one as yellow, with no apparent size dichotomy. The dark phenotype could be due to ontogenetic pigment variation or it is possible that not only *M*. *pentadactyla* is being observed, but also *M*. *crassicaudata* or *M*. *javanica*. Both forms appear to be strictly terrestrial which suggests the more arboreal *M*. *javanica* may not be present. Hunters claim that the two forms are syntopic. Additional surveys are required to correctly identify which species the dark form belongs to, but due to rampant commercial harvesting in the region, this species may already be extirpated.

### Lawachara National Park

41 non-random, trail-bound transects performed nocturnally by amateur naturalists within LNP yielded one pangolin observation: a 4kg female *M*. *pentadactyla* (S3 File) on June 5^th^, 2016. Off-trail surveys were performed from June 19–20, 2016, conducted by three of our parabiologists utilizing their traditional ecological knowledge. This survey effort, resulted in the capture and release of a single male *M*. *pentadactyla*. The successful capture on the second day was facilitated by a heavy rain clearing the forest of tracks after the first day of unsuccessful tracking. This individual weighed 9.2 kg, well above the reported 7.0 kg maximum weight for a male *M*. *pentadactyla* [30]. Photo vouchers were submitted to the Zoological Resources Collection, National University of Singapore and accepted into the catalog as ZRC(IMG) 4.1a (S4 File) and ZRC(IMG) 4.1b (S5 File). These photo vouchers are not yet available online. Despite the degraded habitat in LNP, pangolin sign was common and the parabiologists were confident that pangolins are currently more abundant in LNP than in any area in the CHT. Additional surveys and studies are planned to quantitatively assess density and home range within LNP.

Through the local contacts acquired during outreach programs in LNP, several reports were received of the laborers opportunistically hunting pangolins for local consumption. Unlike most Bangladeshi citizens who are primarily Muslim and refuse to eat pangolins, migrant tea plantation workers are primarily Hindu and have no religious barriers to consuming meat sourced from the nearby forest. Tea plantation laborers also report the occurrence of pangolins well within the monoculture tea estates.

### Media queries

**Fig 2:**
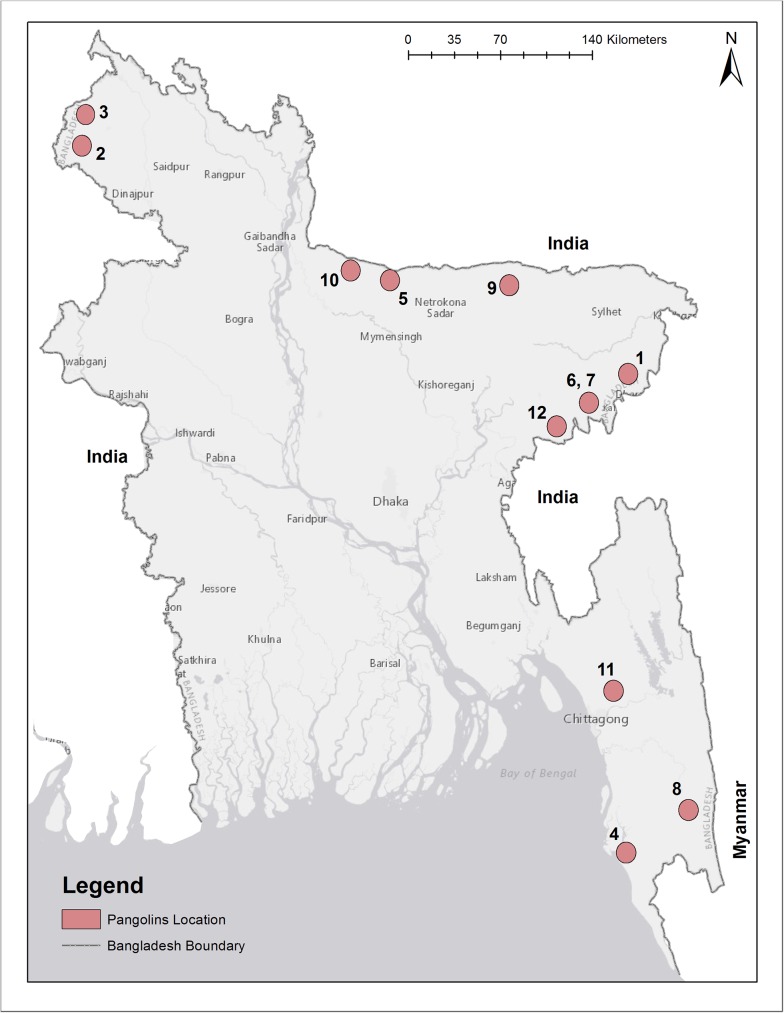
Map of Bangladesh pangolin occurrences. All currently known Pangolin occurrences in Bangladesh are contained in this dot locality map. Labels correspond to numbers within Table 1 and S7 File.

Five records of pangolins within Bangladesh were obtained through our online queries. These records were reported from 2010 or later and were associated with identifiable photographs. Approximate locations of reported sightings, along with survey results were plotted in Fig 2 and relevant information from the media records can be found in Table 1. Web links and translations of these records can be found in S6 File. Through these queries we have confirmed two reports of *M*. *pentadactyla* in the northeast region of Bangladesh, and one report of *M*. *pentadactyla* from Cox’s Bazaar in the southeast. A confirmed *M*. *pentadactyla* was reported to have been beaten to death in northwest Bangladesh. A separate report from the same region of a pangolin having been beaten to death was likely to be *M*. *pentadactyla*, but this identification was not able to be confidently confirmed due to the poor quality of the photograph.

**Table 1:** Pangolin occurrence media query results.

#	Date of Report	Species	District	Sub-district	Found	Habitat
**1**	3-May-13	*Manis pentadactyla*	Moulvibazaar	Kuluara	Village	Mixed-evergreen
**2**	20-Oct-14	*Manis pentadactyla*	Thakurgao	Ranishainkol	Village	Dry deciduous
**3**	11-Oct-14	*Manis* sp. ^a^	Thakurgao	Baliadanga	Village	Dry deciduous
**4**	2-Jul-12	*Manis pentadactyla*	Cox's Bazar	Cox's Bazar	Forest	Mixed-evergreen
**5**	5-May-11	*Manis pentadactyla*	Mymensingh	Haluaghat	Forest	Mixed-Evergreen or Deciduous

^a^ Species identification was difficult to obtain from provided picture, but was likely *M*. *pentadactyla*.

A notable record of a *M*. *pentadactyla* discovered in a botanical garden within the greater Dhaka area from 2005 was encountered, but not included in our results as it was reported prior to 2010. Current status notwithstanding, this record, along with observations of *M*. *pentadactyla* utilizing human-modified landscapes such as tea plantations, indicates *M*. *pentadactyla* are likely able to utilize altered and degraded habitat. It is possible that this observation made near an international airport is that of an escaped individual, instead of a naturally occurring individual. Additional surveys are planned to confirm *M*. *pentadactyla* habitat preference in Bangladesh.

## Conclusions

Our preliminary investigation confirms that *M*. *pentadactyla* still occurs in Lawachara National Park, several adjacent protected areas, and occasionally ranges into bordering tea plantations. Unfortunately, our village surveys strongly suggest that *M*. *pentadactyla* were extirpated from most areas of the Chittagong Hill Tracts by 2014 due to the initiation of commercial pangolin scale harvesting in 2010. The ever-decreasing numbers of pangolins in other Asian countries [1,2,15] will continue to increase commercial demand for pangolin scales from Bangladesh. Small, isolated populations likely persist in regions within the CHT where there is an absence of highly-skilled hunters. This pattern appears to be consistent throughout Bangladesh: where forested areas occur, and a paucity of skilled hunters exists, pangolins can persist.

We did not find any recent or historical empirical evidence of *M*. *javanica* or *M*. *crassicaudata* occurring in Bangladesh. Considering their ecology and purported historical distributions, it is possible that *M*. *crassicaudata* persists in regions of northern and western Bangladesh. Prior to 2010 and the initiation of commercial pangolin scale harvesting, *M*. *javanica* may have occurred in the extreme southeast regions, but the likelihood of current occurrence in Bangladesh is perceived to be low. Additional surveys are needed to verify whether these two species are extant in Bangladesh, but it is the opinion of the authors that recent records of either *M*. *javanica* or *M*. *crassicaudata* within Bangladesh are likely misidentified *M*. *pentadactyla*.

We recommend that pangolin presence/absence surveys be carried out with the aid of traditional ecological knowledge. Employing TEK proved to be more insightful, accurate, and efficient than traditional survey methods such as camera trapping and nocturnal transect surveys. Additionally, utilizing TEK increases the likelihood that surveys will occur in areas inhabited by target species. Corresponding TEK provided by our parabiologists largely agrees with the published literature, and therefore can likely be relied upon with reasonable confidence in lieu of published data. Through the utilization of TEK, novel natural history insight was gained at a more rapid rate and economically efficient manner than additional scientific studies would allow. Increased rate in knowledge acquisition is a powerful tool in dire circumstances such as that of the pangolin’s, which requires a reduced research time-frame in order to establish necessary, immediate conservation actions.

## Supporting information

S1 File Photograph of *M*. *pentadactyla* by Monirul Khan 2007. (JPG)

S2 File Photograph of Ahsan Pangolin Observation. (JPG)

S3 File Photograph of female *M*. *pentadactyla* from LNP. (JPG)

S4 File Photograph of male *M*. *pentadactyla—*ZRC(IMG) 4.1a. (JPG)

S5 File Photograph of male *M*. *pentadactyla—*ZRC(IMG) 4.1b. (JPG)

S6 File Translations and Web Links for Media Query Results. (DOCX)

S7 File Pangolin Occurrence Records. (XLSX)

S8 File CHT Report. (PDF)

## Abbreviations

LNP: Lawachara National Park
CHT: Chittagong Hill Tracts
TEK: traditional ecological knowledge

## References

[1] Challender D, Baillie J, Ades G, Kaspal P, Chan B, Khatiwada A, et al. Manis pentadactyla. The IUCN Red List of Threatened Species. 2014.

[2] Challender D, Nguyen T, Shepherd C, Krishnasamy K, Wang A, Lee B, et al. Manis javanica. The IUCN Red List of Threatened Species. 2014.

[3] Baillie J, Challender D, Kaspal P, Khatiwada A, Mohapatra R, Nash H. Manis crassicaudata. The IUCN Red List of Threatened Species. 2014.

[4] Reza A.H.M. Diversity of amphibians and reptiles in Bangladesh: Impact of climate change, biogeography, and conservation management of herpetofauna in Bangladesh. Saarbrücken, Germany: Lap Lambert Academic Publishing; 2010.

[5] Choudrury A. On the pangolin and porcupine species of Bangladesh. Journal of the Bombay Natural History Society. 2004;101(3): 444–445.

[6] Zhou Z, Zhou Y, Newman C, Macdonald D. Scaling up pangolin protection in China. Frontiers in Ecology and the Environment. 2014;12: 97–98.

[7] Khan MMH. Protected areas of Bangladesh–a guide to wildlife. Dhaka, Bangladesh: Nishorgo Program, Bangladesh Forest Department; 2008.

[8] Khan MAR. Mammals of Bangladesh: A field guide. Dhaka, Bangladesh: Nazma Reza; 1985.

[9] Khan MAR. Wildlife of Bangladesh from amphibia to mammalia–a checklist. Dhaka, Bangladesh: Shahitya Prakash; 2010.

[10] Lagrada L, Schoppe S, Challender D. Manis culionensis. The IUCN Red List of Threatened Species. 2014.

[11] Bangladesh CITES MA, 1986. In litt. to IUCN Conservation Monitoring Centre. 1986.

[12] Agrawal V, Das P, Chakraborty S, Ghose R, Mandal A, Chakraborty T, et al Mammalian: Fauna of West Bengal, state fauna series 3. Calcutta, India: Zoological Survey of India; 1992.

[13] IUCN Bangladesh. Bangladesher bipanno bonno prani (Threatened wild animals of Bangladesh). Dhaka, Bangladesh: IUCN–The World Conservation Union; 2003.

[14] Ahmed ZU, Begum ZNT, Hassan MA, Khondker M, Kabir SMH, Ahmad M, et al Encyclopedia of flora and fauna of Bangladesh, volume 27: Mammals. Dhaka, Bangladesh: Asiatic Society of Bangladesh; 2009.

[15] Ahsan MF, Chowdhury MA. W. Mammals of the Chittagong University campus, Chittagong. Bangladesh: J. Zool. 2008;36(2): 131–147.

[16] Heath ME. Manis crassicaudata. Mammalian Species. 1995;513: 1–4.

[17] WCMC, IUCN/SSC, TRAFFIC. Review of significant trade in animal species included in CITES Appendix II: Detailed reviews of 37 species. Cambridge, UK: World Conservation Monitoring Centre, IUCN Species Survival Commission and TRAFFIC Network; 1999.

[18] Platt SG, Khin MM, Win KK, Maung A, Rainwater TR. Field observations and conservation of *Heosemys depressa* in the Rakhine Yoma elephant range of western Myanmar. Chelonian Conservation and Biology. 2010;9(1): 114–119.

[19] The World Bank. World Development Indicators. Available from: http://databank.worldbank.org/data/reports.aspx?source=2&country=BGD.

[20] Lewin TH. The hill tracts of Chittagong and the dwellers therein. Calcutta, India: Bengal Printing Company Limited; 1869.

[21] Rasul G. Political ecology of degradation of forest common in the Chittagong Hill Tracts of Bangladesh. Environmental Conservation. 2007;34: 153–163.

[22] Mahony S, Reza A. A herpetofaunal collection from the Chittagong Hill Tracts, Bangladesh, with two new species records for the country. Hamadryad. 2008;32(1): 34–35.

[23] Hansen MC, Potapov PV, Moore R, Hancher M, Turubanova SA, Tyukavina A, et al High-resolution global maps of 21st-century forest cover change. Science. 2013 11 15. 10.1126/science.1244693 24233722

[24] Ahmed ZU., Begum ZNT, Hassan MA, Khondker M, Kabir SMH, Ahmad M, et al Encyclopedia of flora and fauna of Bangladesh, angiosperms: Monocotyledons (Volume 12). Dhaka, Bangladesh: Asiatic Society of Bangladesh; 2008.

[25] Huntington HP. Using traditional ecological knowledge in science: Methods and Applications. Ecological Applications. 2000;10: 1270–1274.

[26] Athreya V, Srivathsa A, Puri M, Karanth KK, Kumar NS, Karanth KU. Spotted in the news: Using media reports to examine leopard distribution, depredation, and management practices outside protected areas in southern India. PLoS ONE. 2015;10(11): e0142647 doi: 10.1371/journal.pone.0142647 26556229 10.1371/journal.pone.0142647 PMC4640542

[27] Katuwal HB, Neupane KR, Adhikari D, Sharma M, Thapa S. Pangolins in eastern Nepal: Trade and ethno-medicinal importance. Journal of Threatened Taxa. 2015;7(9): 7563–7567.

[28] Lin, JS. Range and burrow utilization in Formosan pangolins (*Manis pentadactyla pentadactyla*) at Luanshan, Taitung. M.Sc. Thesis, National Pingtung University of Science and Technology. 2011. Available from: http://aa.npust.edu.tw/htm/832-932-grade/991%E6%91%98%E8%A6%81/M9617012.doc

[29] Nowak R. Walker's mammals of the world. Baltimore: The Johns Hopkins University Press; 1999.

[30] Heath ME. Manis pentadactyla. Mammalian Species. 1992;414: 1–6.

