# Supplementary figures and images for: Pangolin distribution and conservation status in Bangladesh
Source: PLoS One. 2017 Apr 7;12(4):e0175450. doi: 10.1371/journal.pone.0175450 (PMC5384767; doi:10.1371/journal.pone.0175450)

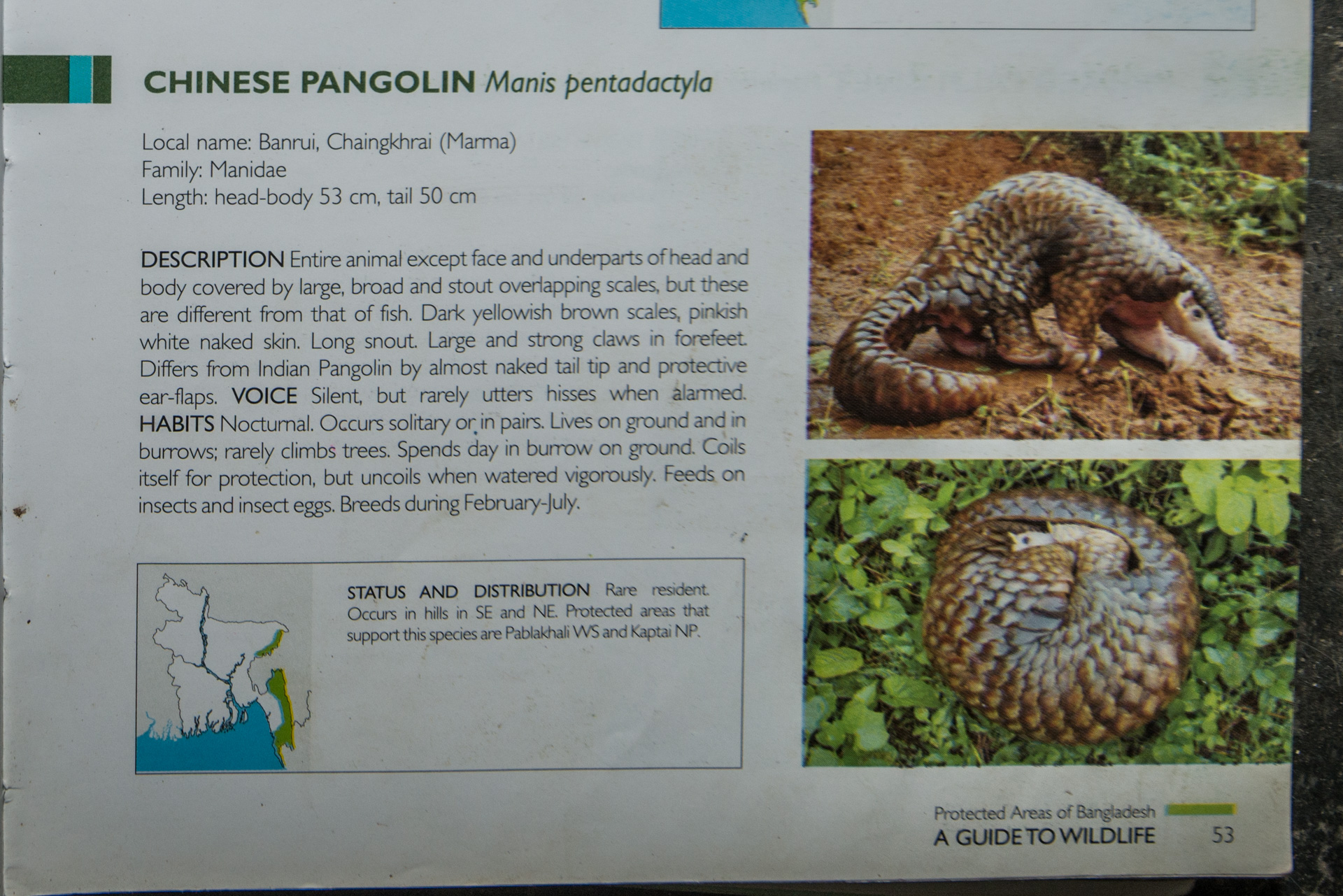

Supplement: S1 File — (JPG) [file pone.0175450.s001.jpg]

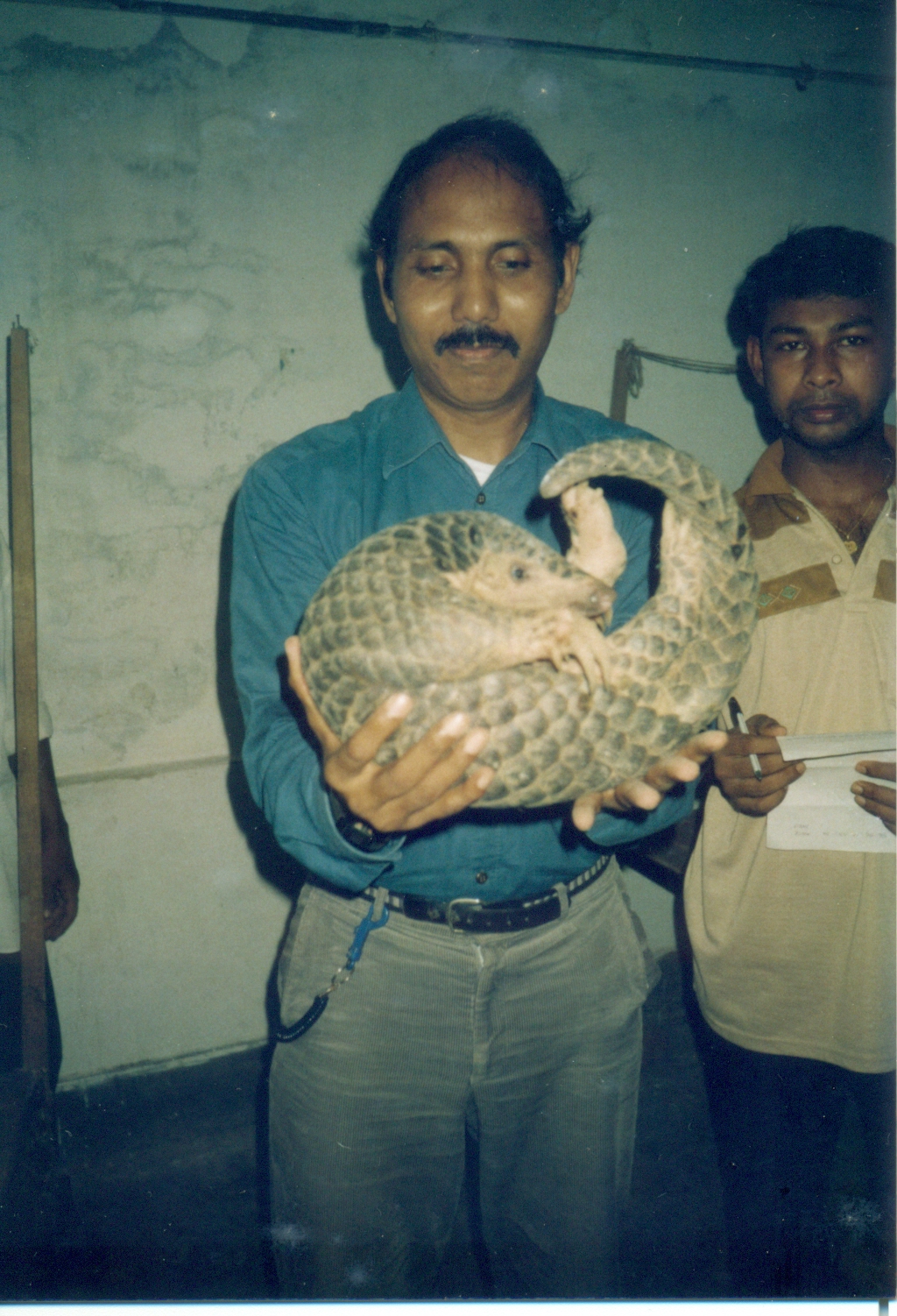

Supplement: S2 File — (JPG) [file pone.0175450.s002.jpg]

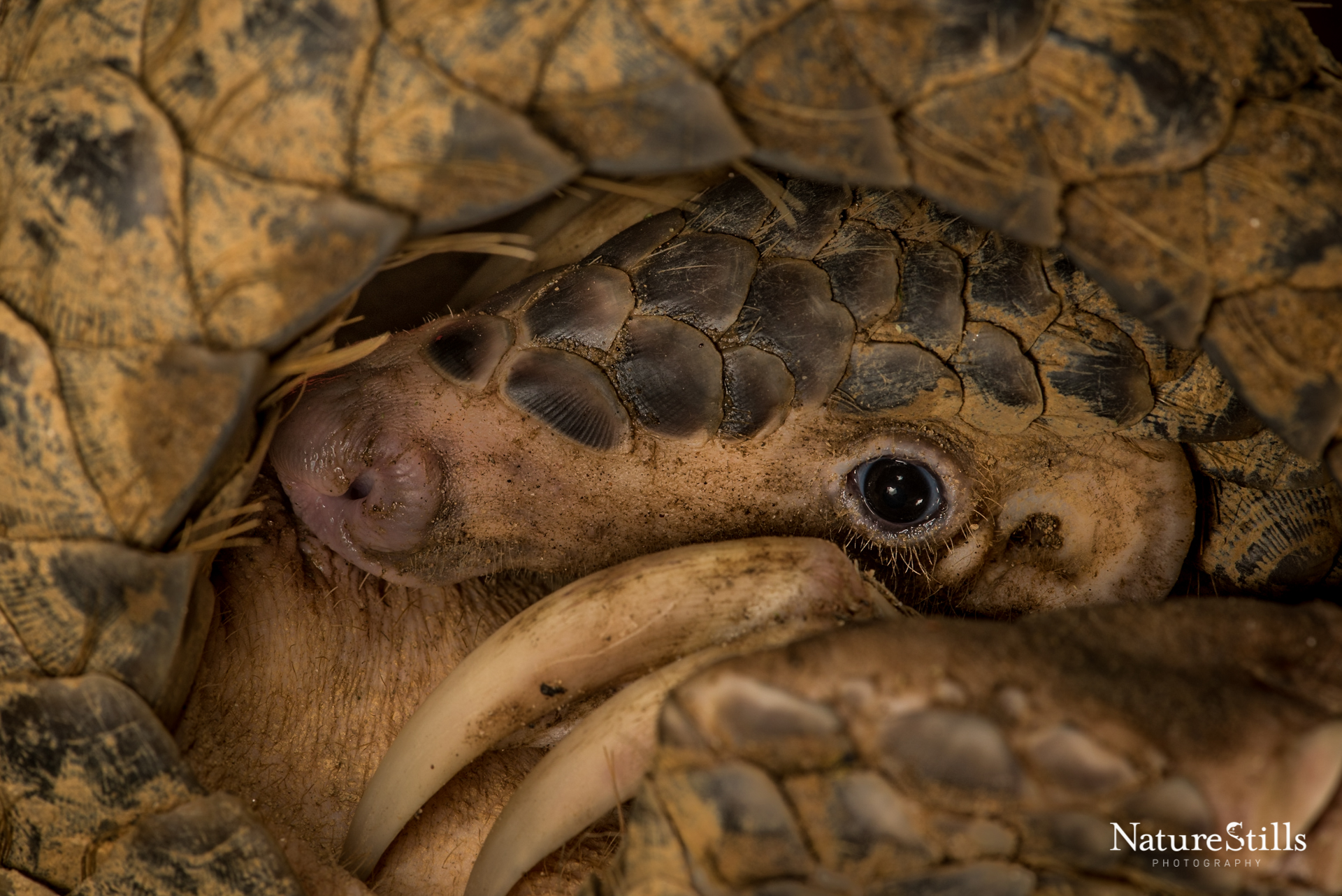

Supplement: S3 File — (JPG) [file pone.0175450.s003.jpg]

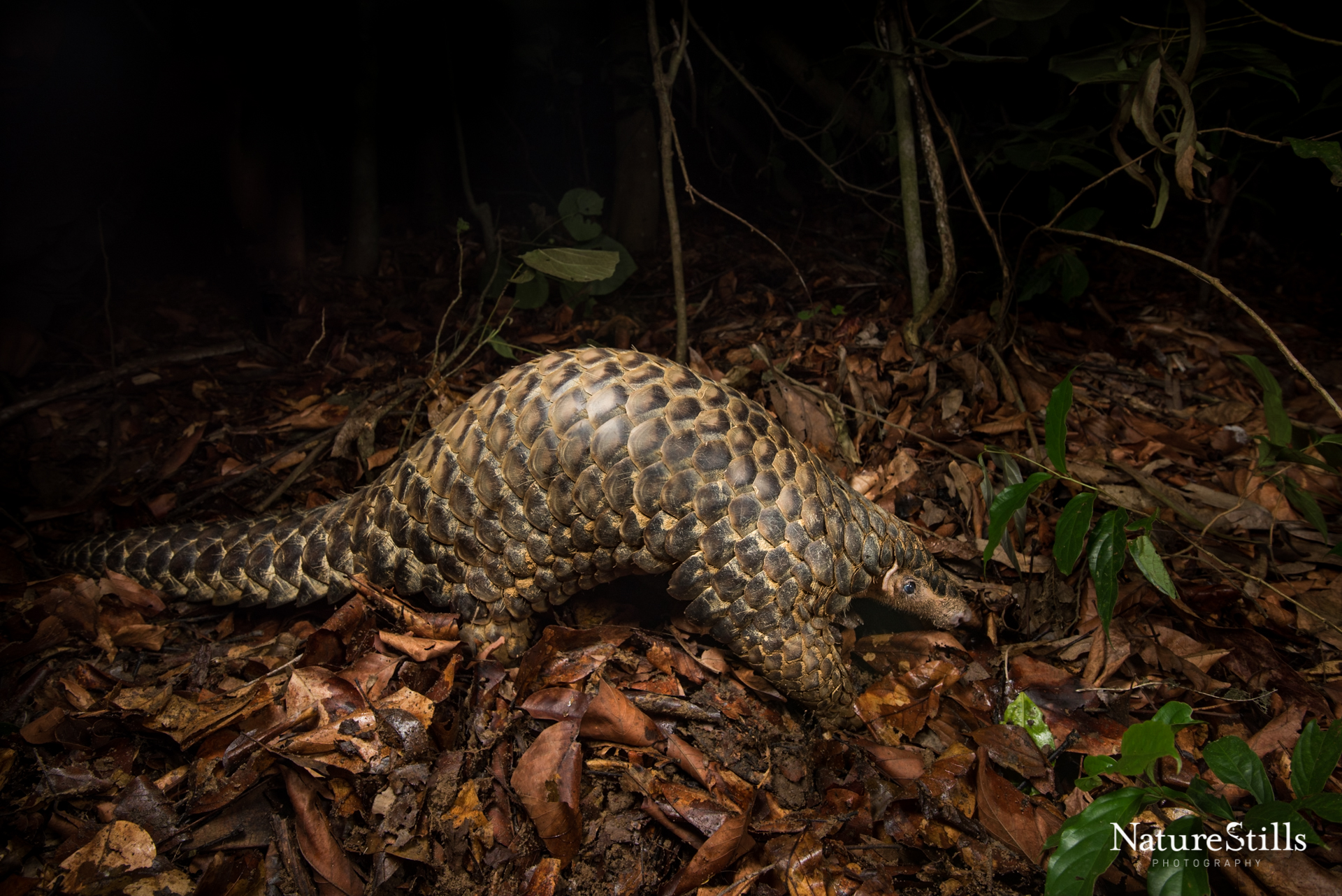

Supplement: S4 File — (JPG) [file pone.0175450.s004.jpg]

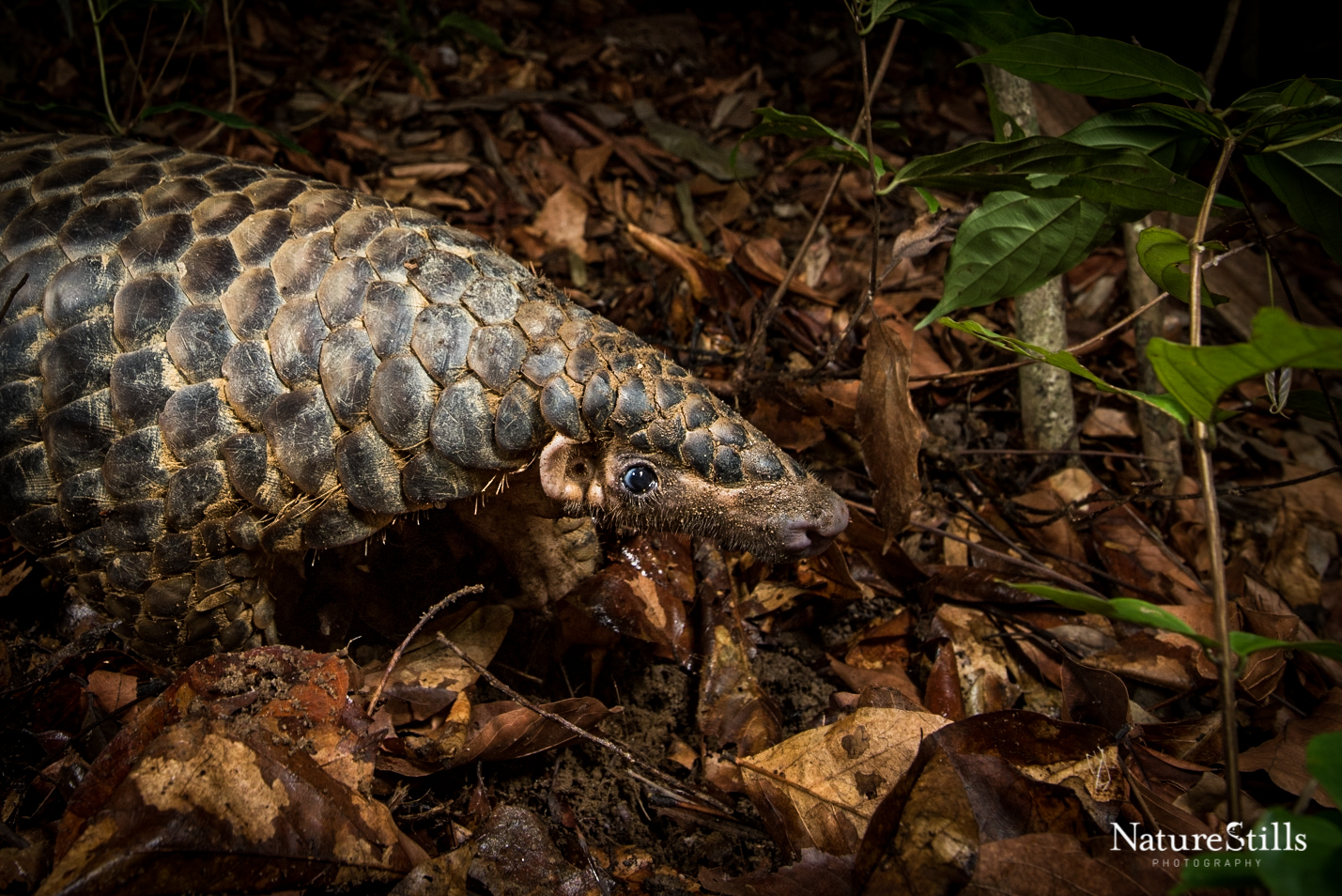

Supplement: S5 File — (JPG) [file pone.0175450.s005.jpg]
