# Supplementary material for: Pangolin distribution and conservation status in Bangladesh
Source: PLoS One. 2017 Apr 7;12(4):e0175450. doi: 10.1371/journal.pone.0175450 (PMC5384767; doi:10.1371/journal.pone.0175450)
Supplement: S6 File — (DOCX) [file pone.0175450.s006.docx]

**Translated Media Query Results**

**Pangolin captured in Tahirpur( Tahirpur/ Sunamganj)**

**9/1/2015 – Northeast Bangladesh**

**Source -** http://bdtoday24.com/%E0%A6%A4%E0%A6%BE%E0%A6%B9%E0%A6%BF%E0%A6%B0%E0%A6%AA%E0%A7%81%E0%A6%B0%E0%A7%87-%E0%A6%A7%E0%A6%B0%E0%A6%BE-%E0%A6%AA%E0%A7%9C%E0%A7%87%E0%A6%9B%E0%A7%87-%E0%A6%AC%E0%A6%A8%E0%A6%B0%E0%A7%81/

An animal named Pangolin have been captured at Tahirpur Border in Sunamganj District. The animal has 4 legs, 1 tail and the front of the head is a bit long and down to the bottom. On sunday a man captured it from a passing stream beside Lakma Madrasah. The creature is unknown to the people of the area which is why a huge crowd gathered to see the animal.

**The scaly strange thing (Nalitabari/Sherpur)**

**3/13/2015 – Central North Bangladesh**

**Source-** http://www.thedailystar.net/backpage/the-scaly-strange-thing-71343

This pangolin, a kind of ant-eater, is locally known as bonrui -- ruhi fish of the forest -- and is hunted for its meat.

One fine morning the man came with his strange booty. He called it Bonrui -- forest ruhi fish.

I had never seen anything like this. Yes, it had the scales like a fish. Its whole body was covered in scales. But then it had four legs and a tail too. How could it be a fish then?

As I searched for an answer, the man freed the strange animal. It looked at us with its black unblinking eyes and then with total disregard for us started slowly moving around.

We noticed that it did not have any mouth as we are used to know. Rather it had a long, slender tongue like a snake which it started pushing out and in of a hole where its mouth should have been.

There was an ant colony in our garden. The Bonrui wondered towards it and started picking up the ants with its sleek tongue. Sometimes it would scratch open the colony with its sharp claws.

It took about 15 minutes to finish up the whole colony. Then the man neatly put the mysterious animal back in its wooden box and left.

Later I came to know that it was a pangolin, a kind of ant-eater.

It is the only mammal wholly covered in scales. When under threat it can coil itself and make itself invincible to predators.

With its extraordinarily long tongue which measures almost its body length, it draws up ants and termites from ant hills. Although it is a slow moving animal, it still needs about seven million ants and termites a year to survive. Since they have no teeth, they ingest small stones while sucking up insects. These stones then grind up the food.

These beautiful animals are becoming extinct in Bangladesh and elsewhere for two reasons -- hunting for meat and trafficking. Globally it is the most trafficked animal.

But in Bangladesh they are hunted for meat.

About two years back, I got a call from someone in Modhupur that a Garo man had caught a pangolin. Dr Anisuzzaman and I went there and found the poor animal tied to a tree. It was injured in its front feet. It looked scared.

The Garo man described how delicious its meat is and how they hunt it at night.

“But these days we hardy get them,” the Garo said. “It's become so rare.”

After much persuasion we convinced the man to hand the animal to us to be released in the forest. We took it to Nalitapbari and released it in the forest.

The animal was all rolled up when it put it on the ground. Then it slowly unwound and looked at us with its dreary eyes. Then it turned around and ran as best as it could into the forest.

I have no idea whether it survived. But it should have enough food in the forest. Pangolins actually play a vital role in maintaining forest health. They eat up the ants and fireflies and keep the trees healthy.

I imagine our pangolin is playing its role in the Nalitabari forest and that it has found a partner. We can only hope they will breed and keep the number up.

**Endangered Species Pangolin Beaten to Death in Ranishongkoil (Ranishongkoil/Thakurgao)**

**10/19/2014 – Northwest Bangladesh**

**Source -** http://www.bd24live.com/bangla/article/8274/index.html

An endangered species Pangolin have been beaten to death on Sunday at Ranishongkoil Bhukurgao village in Thakurgao. A local man named Suresh (25) saw the Pangolin when he went for fishing next to his house. After seeing the creature he hit it with a stick and killed it. The S.I. of Ranishongkoil Thana rescued the Pangolin's body and take it to the Police Station. Thousands of local people gathered there to see the creature. Last 11th October another villager saw a Pangolin entering their kitchen and cried out in terror. Their neighbor heard them and captured the Pangolin and beated to death.

**Pangolin Beaten to Death (Baliadangi/ Thakurgao)**

**10/11/2014 – Northwest Bangladesh**

**Source -** http://www.banglanews24.com/climate%C2%ADnature/news/330902/%E0%A6%AA%E0%A6%BF%E0%A6%9F%E0%A6%BF%E0%A7%9F%E0%A7%87%C2%AD%E0%A6%25%E2%80%A6

An endangered species of Pangolin beaten to death by the people of Fatehpur village at Baliadangi Upazila in Thakurgao. After getting the news a group of people tried to take the hide from the animal. A villager Moslema, Jamal Uddin's wife said, Thursday night the animal got into their kitchen. They got scared and cried out in terror. Their neighbors came after hearing them and grabbed the creature. Later that night they beat the animal to death and Friday morning, they hung the animal to a tree. In the afternoon a few people came to take the hide of the animal but the local didn't let them take it, said by a local person named Sirajul Islam. A man from neighbor village said, He never saw anything like this creature before.

**A Pangolin captured and killed by local people at Kulaura (Kulaura/Moulovibazar)**

**5/3/2013 – Northeast Bangladesh**

**Source -** http://www.jurinews.com.bd/%E0%A6%95%E0%A7%81%E0%A6%B2%E0%A6%BE%E0%A6%89%E0%A6%A1%E0%A6%BC%E0%A6%BE%E0%A6%AF%E0%A6%BC-%E0%A6%AC%E0%A6%BF%E0%A6%B0%E0%A6%B2-%E0%A6%AA%E0%A7%8D%E0%A6%B0%E0%A6%9C%E0%A6%BE%E0%A6%A4%E0%A7%80%E0%A6%AF/

The local people of Karmadha union in Kulaura upazila captured a endangered Pangolin. After informing the Forest Department the officials delayed to reach the place. In the mean time the locals beat the Pangolin to death. According to the local people the Pangolin fell into the well of Giash Miah's house at Gutumpur Tilabari in Karmadha union. They are assuming that the Pangolin fell into the well during the Kal Baishakhi Storm on May 1, Wednesday. The next day May 2nd they pull out the Pangolin from the well. The matter is informed to the District officer and he informed the Range officer to rescue it. But they delayed to go and reach there at 4 p.m. In the mean time some people have beaten the Pangolin to death. Later when it was up for sale the Forest Department officials found the dead body of it. The Range officer and forest officials then captured and took it to Kulaura Livestock Hospital.

**Endangered Pangolin rescued from Cox's Bazar (Sadar/ Cox's Bazar)**

**7/3/2012 – Southeast Bangladesh**

**Source-** http://www.dainikazadi.org/details2.php?news_id=313&table=july2012&date=2012%AD07%AD03&page_id=6

A endangered Pangolin rescued in Cox's Bazar. It is called 'Khudak' in local language. Yesterday it was brought by a man to sell it in the city. The local youth saw that and informed the forest department. The vendor got alert and fled from there. Later the Forest Department official came and rescued it. The Pangolin weighs about 4kg. The officials assuming that the species lost its habitat due to flood which is why they are being caught by the people. Gazi Asmat, Professor of zoology at the University of Chittagong said that, this is an endangered species of Bangladesh. Sometime people kill them for their prejudices and sometimes fortunetellers cheat people by using them. But actually it is very beneficial animal for human. They clean the environment by eating insects but never harms human. It eats a lot of insects every day. Their favorite food is ants. For the sake of environment it's important to save this species, He also added. They weight about 2-9kgs. These nocturnal animals are very shy and mostly loves to stay in their pit. They can dig up to 9 feet long pit within 3-5 minutes of time. They can even move fast on tress too. These creatures are also found in India, Eastern Himalayas, China, Nepal, and Myanmar. It's meat is very popular in Hong Kong.

**Pangolin Released in Eco Park (Haluaghat/Mymensingh)**

**5/5/2011 – Northeast Bangladesh**

**Source -** http://www.thedailystar.net/news-detail-184306

An Indian Pangolin locally known as Bonrui (because it has fish-like scaly body) was captured in Haluaghat last week by a Garo man. It was later released into Madhutila Eco Park with the help of some nature lovers and forest department people. Pangolin is an anteater and a mammal now endangered in Bangladesh. Dr Reza Khan, a wildlife expert, says this animal used to be widely distributed over the country excluding the coastal parts of Khulna, Barisal, Patuakhali, Noakhali and Chittagong. It has possibly disappeared from the Kushtia, Jessore, Pabna, Bogra, Rangpur, Dinajpur, Rajshahi and most parts of Dhaka and Comilla regions. Loss of habitat and poaching are the main reasons for its disappearance. The species currently found in small numbers in the Shal, evergreen and semi-evergreen forests.
