# Supplementary material for: Pangolin distribution and conservation status in Bangladesh
Source: PLoS One. 2017 Apr 7;12(4):e0175450. doi: 10.1371/journal.pone.0175450 (PMC5384767; doi:10.1371/journal.pone.0175450)
Supplement: S8 File — (PDF) [file pone.0175450.s008.pdf]

Creative Conservation Alliance

A Report Submitted to
Bangladesh Forest Department

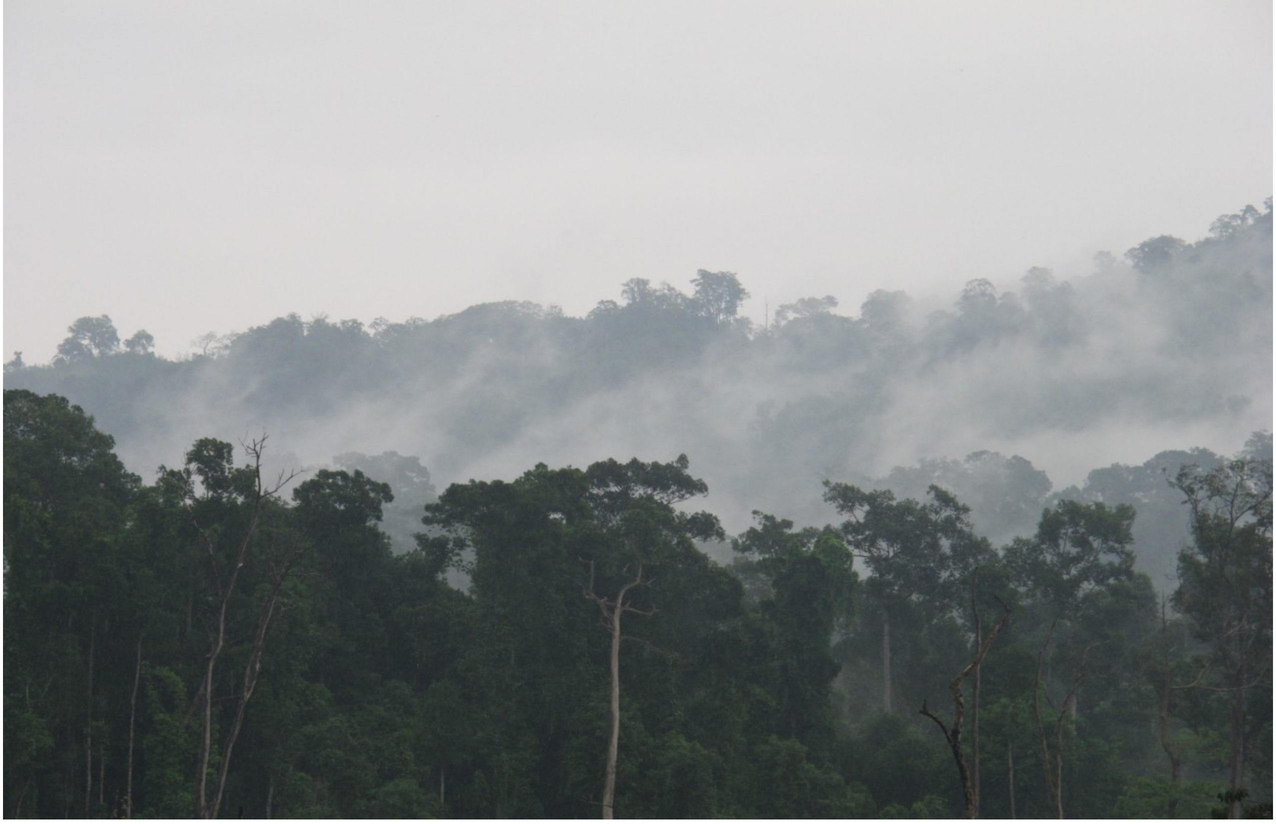

A Preliminary Wildlife Survey in Sangu-Matamuhuri Reserve Forest, Chittagong Hill Tracts, Bangladesh

Address: 1/4, Block-C, Lalmatia, Dhaka-1207; **Email:** creativeconservationalliance@gmail.com

A Preliminary Wildlife Survey of Sangu-Matamuhuri Reserve Forest, Chittagong Hill Tracts, Bangladesh

Submitted to:

Bangladesh Forest Department
Ministry of Forest and Environment
Bangladesh

Submitted By:

Creative Conservation Alliance
Lalmatia, Dhaka, Bangladesh

April 2016

Citation: Creative Conservation Alliance. (2016). A preliminary wildlife survey in Sangu-MatamuhuriReserve Forest, Chittagong Hill Tracts, Bangladesh. Unpublished report submitted to Bangladesh Forest Department, Dhaka, Bangladesh.

Cover Photo: A patch of old growth forest in Sangu Reserve Forest

Creative Conservation Alliance

Acknowledgements

Creative Conservation Alliance is grateful to Bangladesh Forest Department for providing necessary support and allowing us to publish this report. We are also thankful to Bangladesh Army (Alikadam Zone) for their support. The funding for this work was provided by Turtle Survival Alliance through a grant from Turtle Conservation Fund and Mohammed Bin Zayed Species Conservation Fund. Lastly, we thank the local ethnic communities in Alikadam and Thanchi for helping us in many ways during the work period.

Table of Contents

Content	Page No.
**1.** Foreword	5
**2.** Executive summary	6
**3.** Background	8
**4.** Objectives	10
**5.** Methodology	11
**6.** Survey Results	14
**6.1.** Mammals	14
**6.2.** Birds	25
**6.3.** Reptiles and Amphibians	26
**6.4.** The People	29
**6.5.** Subsistence Hunting Practice	33
**7.** Threats to Biological Diversity	40
**8.** Way Forward?	44
**9.** Appendix	45
**10.** Reference	50

1. Foreword

Very few areas on earth are blessed with an extremely high diversity of life forms-from charismatic mega-fauna to microscopic organisms- together with high endemism. International body of Scientists has designated these areas as “biodiversity hotspots”, which demand for the highest priority for conservation. Among 34 such hotspots on earth, one of these is Indo-Burma Biodiversity hotspot, which has its western end in the Chittagong Hill Tracts (CHT) of Bangladesh, the only hotspot that is shared by Bangladesh. CHT, comprises over 10% of the total land area of Bangladesh, is one of the last strong hold of biodiversity in Bangladesh. CHT harbors many species of fauna and flora, and even more species are yet to be discovered. Despite its importance, CHT remains as the least explored area of Bangladesh, primarily due to the remote nature of the area and its political complexities. Despite facing formidable challenges, Creative Conservation Alliance, has already done some remarkable work in CHT. Primarily, with the help of the local ethnic people they have discovered and re-discovered many globally threatened species in the most remote part of CHT- the Sangu-Matamuhuri Reserve Forest. Their work indicates that Sangu-Matamuhuri Reserve Forest is one of the last strong hold of biodiversity in Bangladesh, where a number of charismatic mega-fauna still roam in the wild. Sadly, however, the amazing wildlife and their unique habitats of Sangu-Matamuhuri Reserve Forest are vanishing rapidly due to poaching, habitat destruction and many other threats. This report provides important insights on the current situation of this important biodiversity hotpot, and based on which, Bangladesh Forest Department can implement further work.

Mohammad Monirul H. Khan, Ph.D
Professor
Department of Zoology
Jahangirnagar University
Savar, Dhaka

2. Executive Summary

The Chittagong Hill Tracts (CHT) comprises 10% of the total land area of Bangladesh and falls within the Indo-Burma Biodiversity Hotspot which renders it, undoubtedly, the richest biodiversity hotspot in Bangladesh. However, due to political instability and the general remoteness of this region, the CHT remains the least explored area in Bangladesh. In particular, we know very little concerning the current status of wildlife within the Sangu and Matamuhuri Reserve Forest. We conducted exploratory surveys in Sangu-Reserve Forest, focusing particularly in Sangu Reserve Forest (SRF), from 2011 to 2015. The interview surveys resulting in hunting records, camera trap surveys, and visual observations were conducted primarily through the assistance of the local communities, including several members of Mro tribe who have been trained as a parabiologists. Some notable findings include: Gaur – the largest cattle species in the world, which was declared extinct by IUCN Bangladesh in 2000; six species of wild cats including Asiatic golden cat, marbled cat, clouded leopard, leopard cat, and tiger. The confirmation of tiger is based on the observation of several pugmarks, identified by experts. We do not have enough evidence to show whether a breeding population of tiger exists within the CHT, or if the pugmarks belong to transient individuals, but the pugmarks do prove that tigers still use this area as part of their home range. Furthermore, we documented both sun bear and Asiatic black bear, dhole/Asiatic wild dog, Asian elephants, sambar, barking deer, wild boar, six species of primates including hoolock gibbon and Phayre's Leaf Monkey, three species of hornbill: great hornbill, wreathed hornbill and oriental-pied hornbill; Burmese python, reticulated python, and king cobra; eight species of turtles and tortoises. There are 16 ethnic settlements within the SRF with a resident human population size of about 1600 people. Human influx, seasonal harvest of forest resources, slash and burn agriculture practice, subsistence hunting and poaching and logging are the major threats for wildlife and its habitat. Our preliminary situational analysis indicates that the SRF is one of the most important sites for wildlife in Bangladesh, and with a proper management plan the SRF can have the long term potential to sustain populations of tigers and elephants. 25 of the observed species are globally threatened-2 Critically Endangered, 9 Endangered and 14 Vulnerable-which deems the SRF an important biodiversity site from not only a national perspective, but also a global perspective.

Executive Summary (in Bangla)

ইন্দো-বার্মা হটস্পট'র অন্তর্ভূক্ত পার্বত্য চট্টগ্রাম এলাকা বাংলাদেশের মোট আয়তনের দশ শতাংশ জুড়ে অবস্থিত। ইন্দো-বার্মা হটস্পটের অন্তর্গত হওয়ায় এই অঞ্চলের জীববৈচিত্র্য অনেক বৈচিত্র্যপূর্ণ। যদিও রাজনৈতিক অস্থিরতা এবং দূরবর্তীতার কারণহেতু এলাকাটি বাংলাদেশের নূন্যতম অন্বেষিত এলাকা হিসেবেই রয়ে গেছে। বিশেষত, সাঙ্গু এবং মাতামুহুরীর রক্ষিত বনাঞ্চলের বন্যপ্রাণিদের বর্তমান অবস্থা সম্পর্কে আমাদের ধারনা খুবই সীমিত।

স্থানীয় ম্রো জনগনের সহায়তায় আমরা ২০১১ থেকে ২০১৫ পর্যন্ত সাঙ্গু রক্ষিত বনাঞ্চল এবং আশপাশে অবস্থিত মাতামুহুরী রক্ষিত বনাঞ্চলে গবেষণামূলক অনুসন্ধান চালিয়েছি। প্রাথমিক অনুসন্ধানে অভ্যন্তরীণ শিকারের তথ্য যোগাড় করার জন্য ক্যামেরা ট্রাপ এবং সম্মুখ পর্যবেক্ষণের দ্বারা নিরীক্ষা চালানো হয় । এ সময়ের মধ্যে স্থানীয় জনগনের সহায়তায় আমরা কিছু উল্লেখযোগ্য বন্যপ্রানি'র সন্ধান এ এলাকায় পাই যা কিনা বাংলাদেশ থেকে বিলুপ্ত হয়ে গেছে বলে মনে করা হয় যেমন : বনগরু/গাউর - বৃহত্তম বন্যগরু প্রজাতি যা ২০০০ সালে 'আইইউসিএন বাংলাদেশ' এর দ্বারা বিলুপ্ত ঘোষনা করা হয়। ছয় প্রজাতির বনবিড়াল যার মধ্যে উল্লেখযোগ্য হল- সোনালী বিড়াল, মরমর বিড়াল, লামচিতা, চিতাবাঘ এবং বাঘ।

বাঘের পায়ের ছাপ পর্যবেক্ষণের দ্বারা বিশেষজ্ঞরা বাঘের উপস্থিতি সম্পর্কে নিশ্চিত হয়েছেন। পার্বত্য চট্টগ্রাম এলাকায় বাঘের বংশবৃদ্ধি হয় কিনা এ সম্পর্কে আমাদের কাছে পর্যাপ্ত তথ্যপ্রমাণ নেই, এমনকি পদচিহ্নগুলো অস্থায়ীভাবে তাদের আগমনের প্রমাণ কিনা এ সম্পর্কেও আমরা নিশ্চিত নই। তবে পদচিহ্নগুলো প্রমাণ করে যে, বাঘগুলো এই এলাকা তাদের আবাসস্থল কিংবা আবাসস্থলের নিকটবর্তী এলাকা হিসেবে ব্যবহার করে থাকে।

এছাড়াও আমরা নিশ্চিত করেছি- দুই প্রজাতির ভাল্লুক যার মধ্যে রয়েছে সূর্য ভাল্লুক এবং কালো ভাল্লুক; বন্য কুকুর; এশীয় হাতি; সাম্বার; মায়া হরিণ; বন্য শূকর; ছয় প্রজাতির বানর যার মধ্যে উল্লেখযোগ্য রয়েছে উল্লুক এবং হনুমান; তিন প্রজাতির ধনেশ পাখি। এছাড়া সরীসৃপ ও উভচর প্রানির মধ্যে রয়েছে আট প্রজাতির কচ্ছপ এবং কাছিম, বার্মিজ অজগর, গোলবাহার অজগর এবং কিং কোবরা এর উপস্থিতি।

সাঙ্গু রক্ষিত বনাঞ্চল'র ১৬টি গ্রামে প্রায় ১৬০০ ক্ষুদ্র নৃ-গোষ্ঠীর মানুষ বসবাস করছে। ক্ষুদ্র নৃ-গোষ্ঠীর জনসংখ্যা বৃদ্ধির সাথে সাথে বনাঞ্চল আবাসভূমিতে স্থানান্তর হওয়া, অনিয়ন্ত্রিতভাবে বনজ সম্পদ আহরন, জুম চাষ , বন্যপ্রানি শিকার এবং অবৈধভাবে গাছ কাটা ইত্যাদি এই বনাঞ্চলের বন ও বন্যপ্রানির জন্য প্রধান হুমকী। আমাদের প্রাথমিক নিরীক্ষা নির্দেশ করছে যে, সাঙ্গু রক্ষিত বনাঞ্চল বাংলাদেশে বন্যপ্রাণির জন্য অন্যতম গুরুত্বপূর্ণ ক্ষেত্র। যথাযথ ব্যবস্থাপনার দ্বারা সাঙ্গু রক্ষিত বনাঞ্চলে দীর্ঘমেয়াদীভাবে বাঘ এবং হাতির সংখ্যাবৃদ্ধির ক্ষেত্র হতে পারে। পর্যবেক্ষণকৃত প্রজাতির ভিতর ২৫ টি প্রজাতি আইইউসিএন'র লাল তালিকার অন্তর্ভুক্ত এর মধ্যে ২টি প্রজাতি মহাবিপন্ন, ৯টি প্রজাতি বিপন্ন ও ১৪টি প্রজাতি সংকটাপন্ন যা সাঙ্গু রক্ষিত বনাঞ্চলকে শুধু জাতীয় প্রেক্ষাপটেই নয় আন্তর্জাতিক পরিমন্ডলে একটি গুরুত্বপূর্ণ বন্যপ্রানি সংরক্ষন ক্ষেত্র হিসেবে গুরুত্ব বহন করে।

3. BACKGROUND

The Chittagong Hill Tracts are part of a 1,800 km-long mountain range oriented from north to south in parallel ridges, incised by deep gorges from the eastern Himalayas in China, to western Myanmar. The CHT comprises 10% of the total land area of Bangladesh (Nath et al. 1998) and lies within the Indo-Burma Biodiversity Hotspot (Myers et al. 2000) with many globally threatened species (Tordoff et al. 2012). Patches of the old-growth, semi-evergreen, and bamboo forest within the CHT has been cleared for commercial teak plantations during the British colonial period. The present-day Forest Department of Bangladesh uses the same practices (Khan 2015). There are three officially protected areas within the CHT:

1) Pablakhali Wildlife Sanctuary, year of establishment: 1962, Area: 42, 087 ha;
2) Kaptai National Park, year of establishment: 1999; Area: 5464 ha;
3) Sangu Wildlife Sanctuary, year of establishment: 2011; Area: 2331 ha;

The 33,836 ha (83,612 acres) Sangu Reserve Forest (SRF) is located in south of Boro Modok in Thanchi Upazila of Bandarban District (Figure 1). Contained within the Sangu Reserve Forest is the Sangu Wildlife Sanctuary (SWF), which was declared by the Forest Department of Bangladesh (Gazzetted on June 4, 2010), however there is no clear demarcation of its boundaries and both the wildlife sanctuaries and reserve forests lack adequate enforcement and jurisdiction. Adjacent to the SRF is the 40,661 ha (100,476 acres) Matamuhuri Reserve Forest (MRF) situated south of Babu Para in Alikadam Upazila of Bandarban District (Figure 1). The MRF has been encroached upon by Bengali and ethnic settlers since the 1980s and as a result, very relatively large patch of natural forest is not left in this area. While the more remote SRF still contains patches of primary forest due to the more recent period of degradation.

The climate of the region is tropical, with a mean annual rainfall of 2,666 mm . A dry, cool season occurs in the region during November-March, followed by a hot and sunny pre-monsoon season during April-May, and a warm, cloudy, and wet monsoon season during June-October (Khan 2015).

The CHT is undoubtedly the richest biodiversity hotspot in Bangladesh. However, due to political instability and the general remoteness of this region, the CHT remains the least explored area in Bangladesh (Khan 2015). As a consequence, there has been very little biodiversity survey work done in the area and no systematic surveys have been carried out. What we know about the biodiversity of this region is primarily based on information generated from historical expeditions in earlier colonial periods. In recent years, Dr. Monirul Khan and Dr. Suprio Chakma have conducted several expeditions in the CHT. These surveys have resulted in several new species of birds, amphibians, and reptiles being recorded for the country. Despite these surveys we still know very little about the

biodiversity of the Sangu Reserve Forest. To fill this knowledge gap, our organization – The Creative Conservation Alliance (CCA) has conducted opportunistic, exploratory surveys in Sangu Reserve Forest, and adjacent areas in Matamuhuri Reserve Forest, from 2011 to 2015, primarily with the assistance of the local ethnic people. The following report contains the preliminary findings from those surveys.

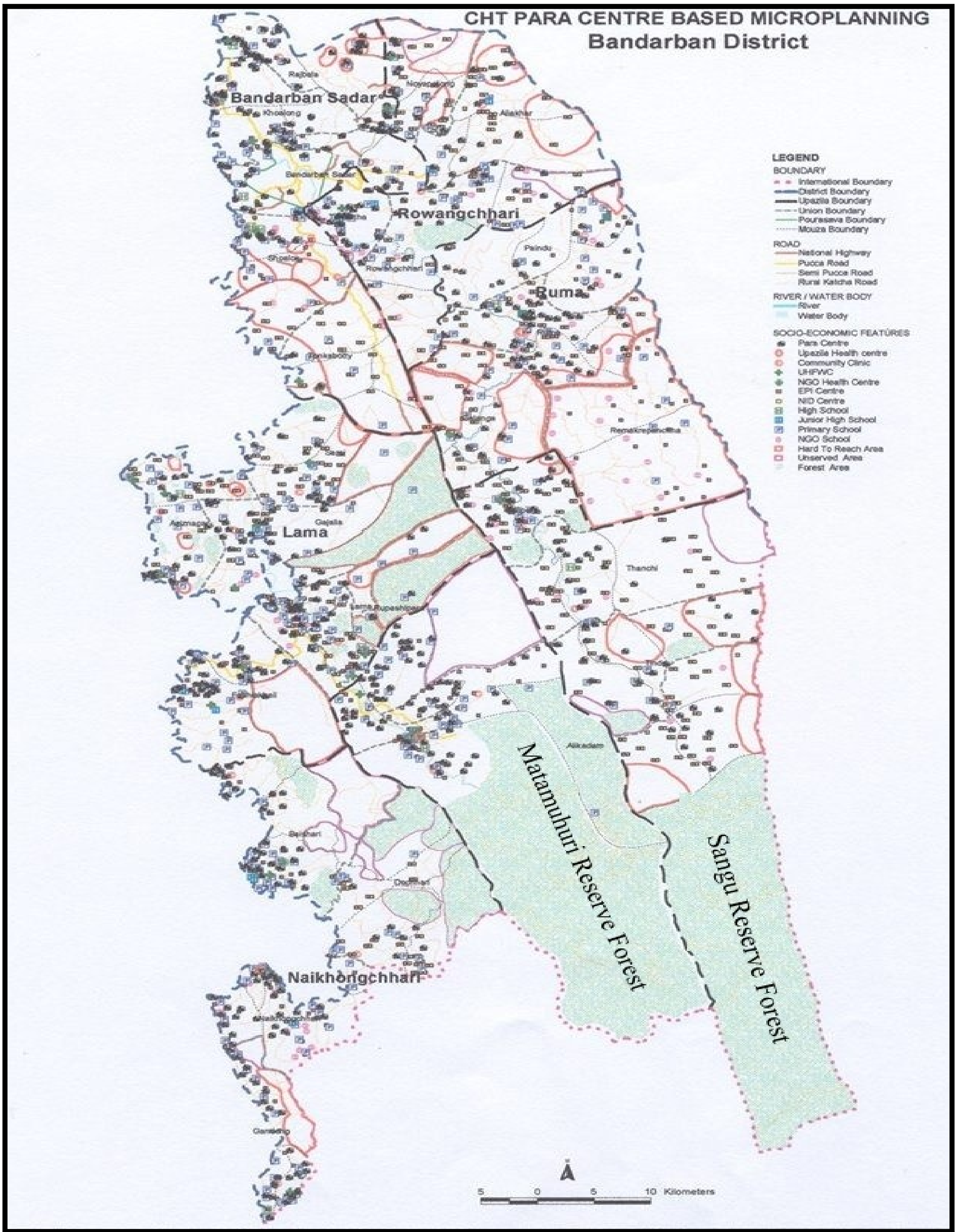

Figure 1: A map of Sangu and Matamuhuri Reserve Forest, Bandarban Disctrict

4. Objectives

1. To conduct exploratory surveys focusing on the biodiversity within the Sangu Reserve Forest and adjacent areas;
2. To gather information on traditional hunting practices of the ethnic people;
3. To identify the major threats to the biodiversity of the region;
4. To conduct a baseline socioeconomic survey of the local ethnic communities;
5. To initiate a preliminary situational analysis of the Sangu Reserve Forest through visual observation and discussions with the local communities;
6. To build rapport and gain the trust of the local communities

5. METHODOLOGY

The surveys were conducted primarily through the assistance of the local communities. Three local community members of Mro tribe were trained to operate digital cameras, camera traps, GPS, handheld weather station, and questionnaire survey techniques. The following strategies were used to collect data:

1. Visits were made in tribal villages to inspect remains of recently hunted specimens;
2. Digital point-and-shoot cameras were distributed to local community members in order to take photographs of live specimens;
3. Camera traps were distributed to previously-trained local community members in order to detect the presence of medium to large-sized mammals.

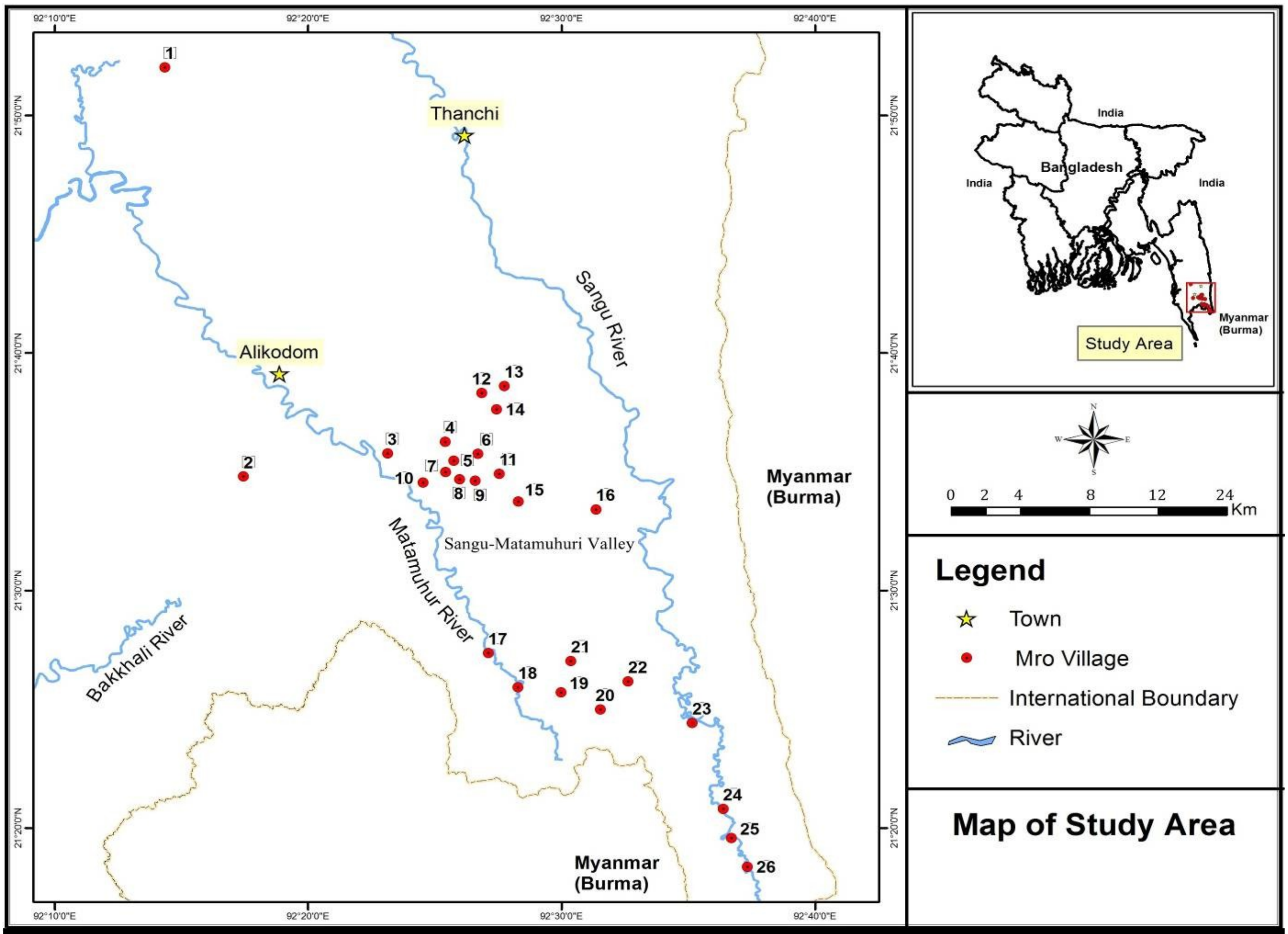

Figure 2: A map of the study area showing the Mro villages visited during the study

Data were collected between December 2011 and August 2015, from 28 of the 33 villages where the survey team has built their rapport. The surveys purposely focused on 28 of the 33 villages where we have the established the strongest trust of the local people. A total of eight visits were made south of

Alikadam and Thanchi town and every visit lasted for at least 12 days. The previously-trained Mro people conducted the survey used a combination of focus group discussions, semi-directive interviews, and collaborative field work (Huntington 2000) to gather information on traditional hunting practices and quantify hunting off take. Ten camera traps were placed from June 2015 to February 2015, totaling over 700 trap/nights.

Interviews were conducted in the evening at the house of a village chief, where a representative from each individual household of the village was present. Each representative was asked to recall the number of wild animals each household had harvested in the previous year. Larger animals (> 2 kg), which are usually consumed communally, were included in the harvest inventories, and tended to have more reliable recall values (Usher and Wenzel 1987, Knapp et al. 2010). Animals such as small mammals (< 2 kg), frogs, snakes, birds, etc. were not included as it would be difficult for villagers to give accurate information. We also gathered information on the population size of the village, average years of field fallows, distance of the village from the nearest town settlements, and number of individuals who own guns and/or actively use traps to capture animals.

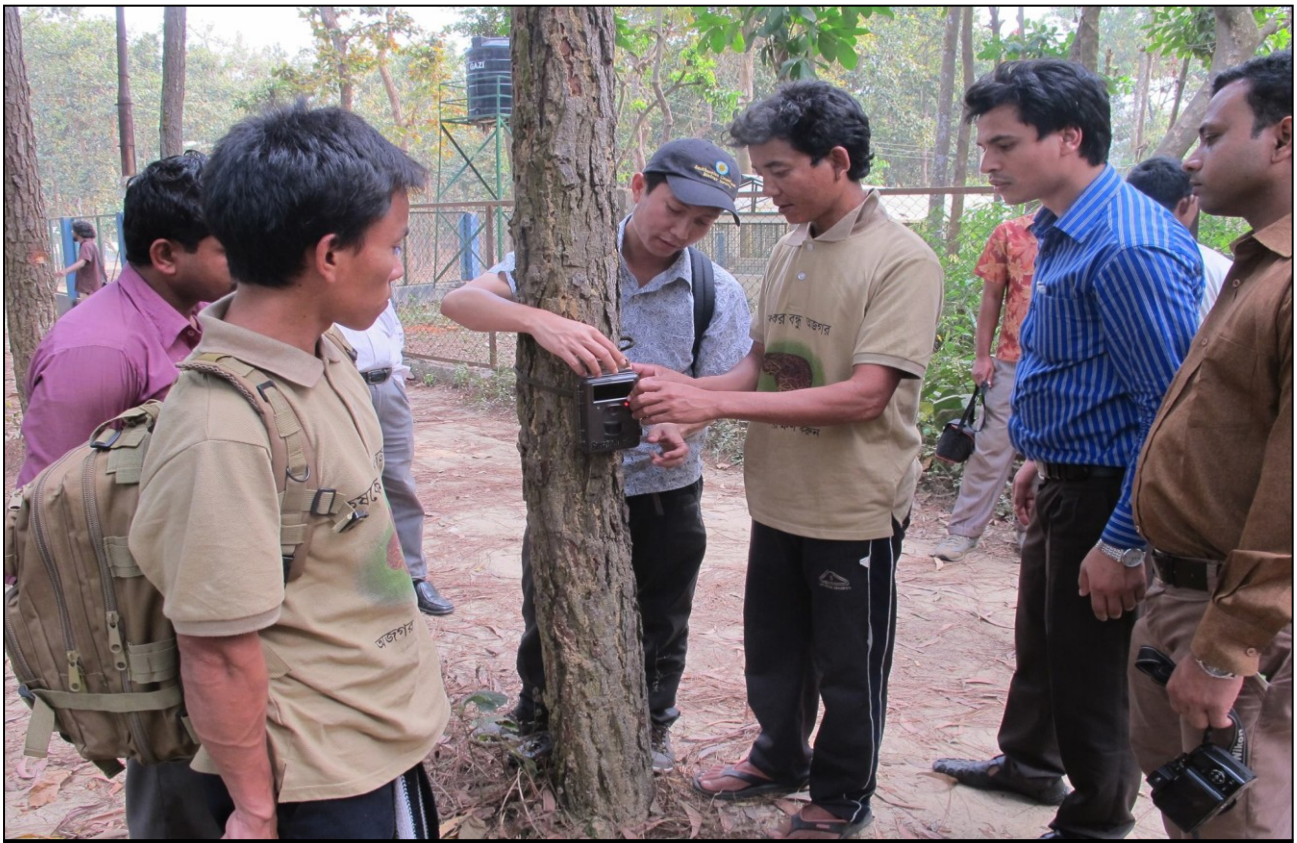

Figure 3: Mro parabiologists during a workshop on camera trapping in Bhawal National Park

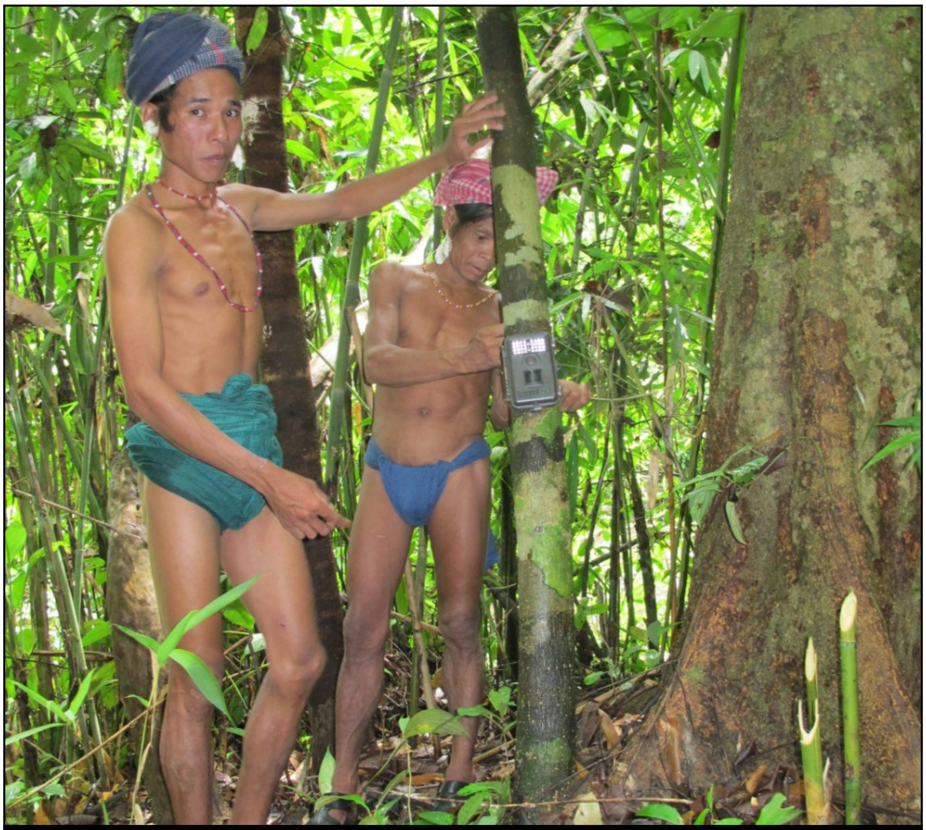

Figure 4: Our parabiologists setting up a camera trap in remote part of SRF

In addition, the team visited each village to interview people regarding targeted species hunted and respective hunting practices. Photographs of species including mammals, reptiles, amphibians, and birds were shown to the interviewees and the corresponding species occurrence, traditional knowledge, and taboos were recorded. A list of species hunted that could be validated by evidence—whole carcasses, skins, or body parts that the villagers had in their possession—was made, noting the hunting techniques used.

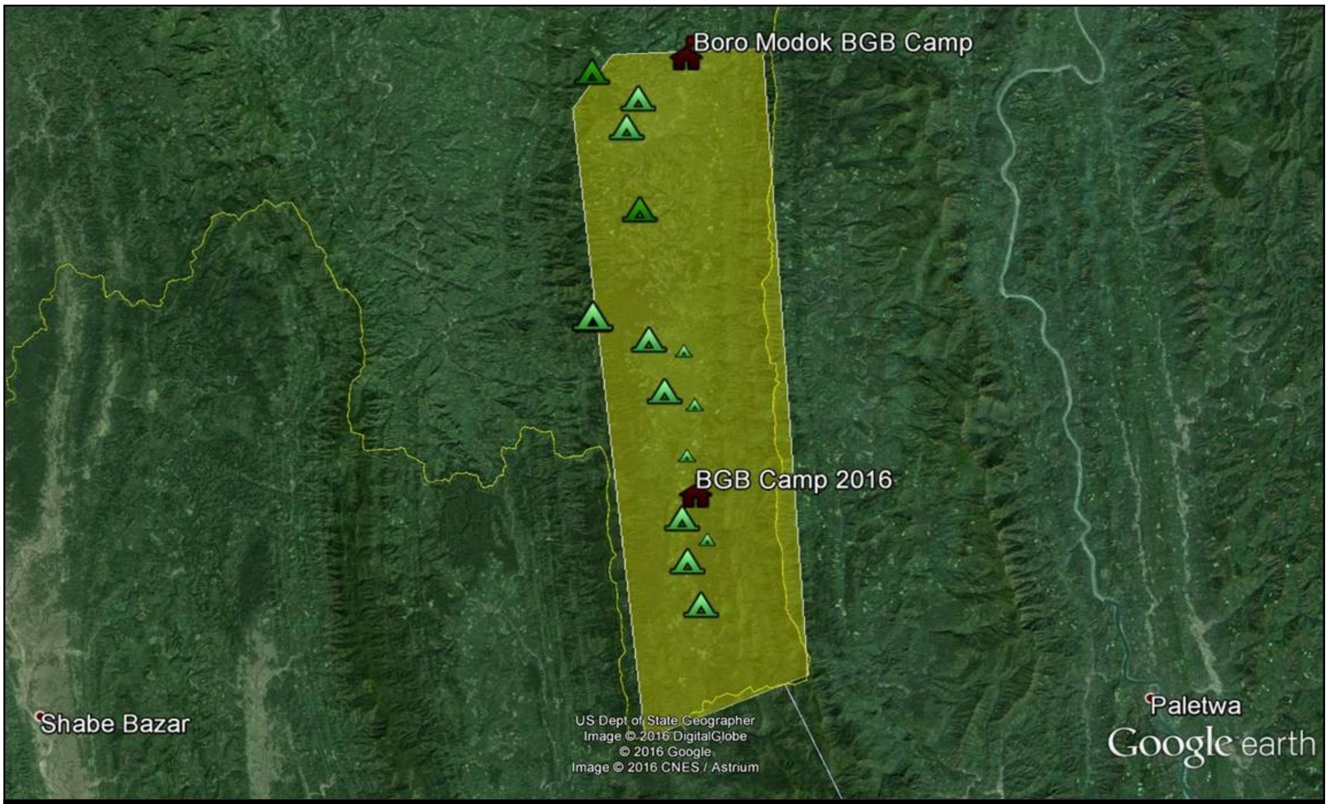

Figure 5: A satellite image showing tribal villages and BGB camps within the SRF (highlighted in yellow)

6. Survey Results

We recorded 37 species of mammals, 46 species of reptiles, 19 species of amphibians, and 11 species of birds during our study period. Following the International Union for Conservation of Nature Red List Classification, some 3% of the species hunted were listed as Critically Endangered, 10% Endangered, 17% Vulnerable, 6% Near Threatened, 59% Least Concern, and 5% Data Deficient.

6.1. Mammals

We confirmed the presence of tiger in the SRF. This confirmation is based on a 14 cm pug mark that we have recorded from one of our study sites in February, 2016. We still do not have any data to show whether a resident population occurs within the CHT or if the pugmarks belong to transient individuals. In several villages, especially in the southern part of the SRF, the locals have mentioned during the interviews that they often see tigers in the area. There have been at least six different sightings of tigers in the area in the last two years. We were also told that Burmese hunters poached two tigers from the area in 2013, and at least two locals have seen mating tigers in the last two years.

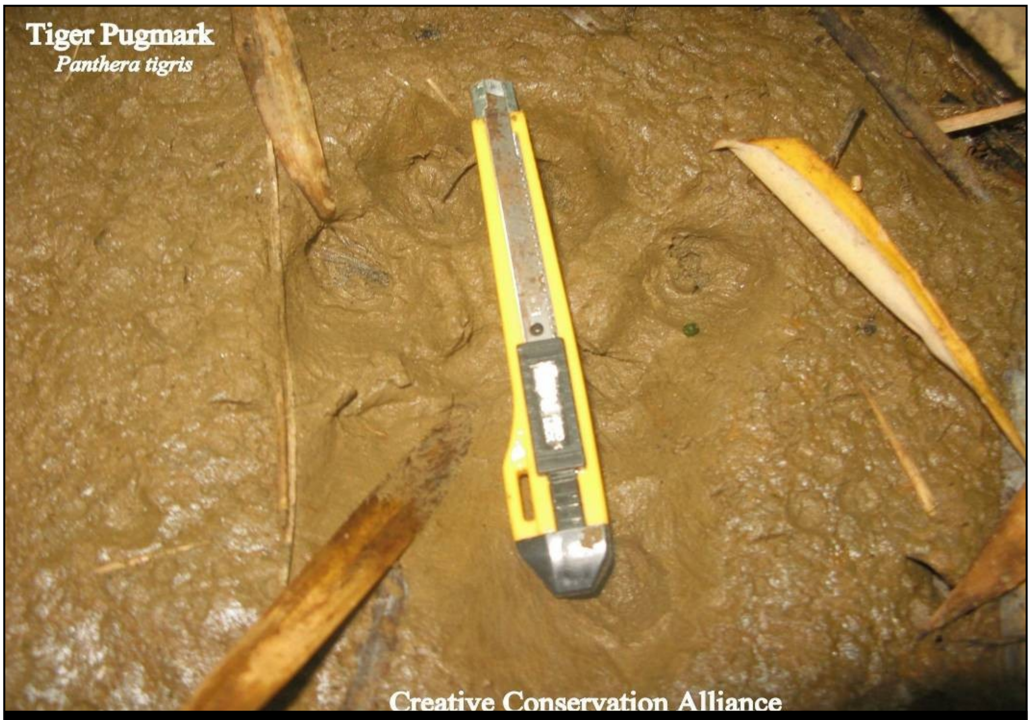

Figure 6: Tiger pugmark

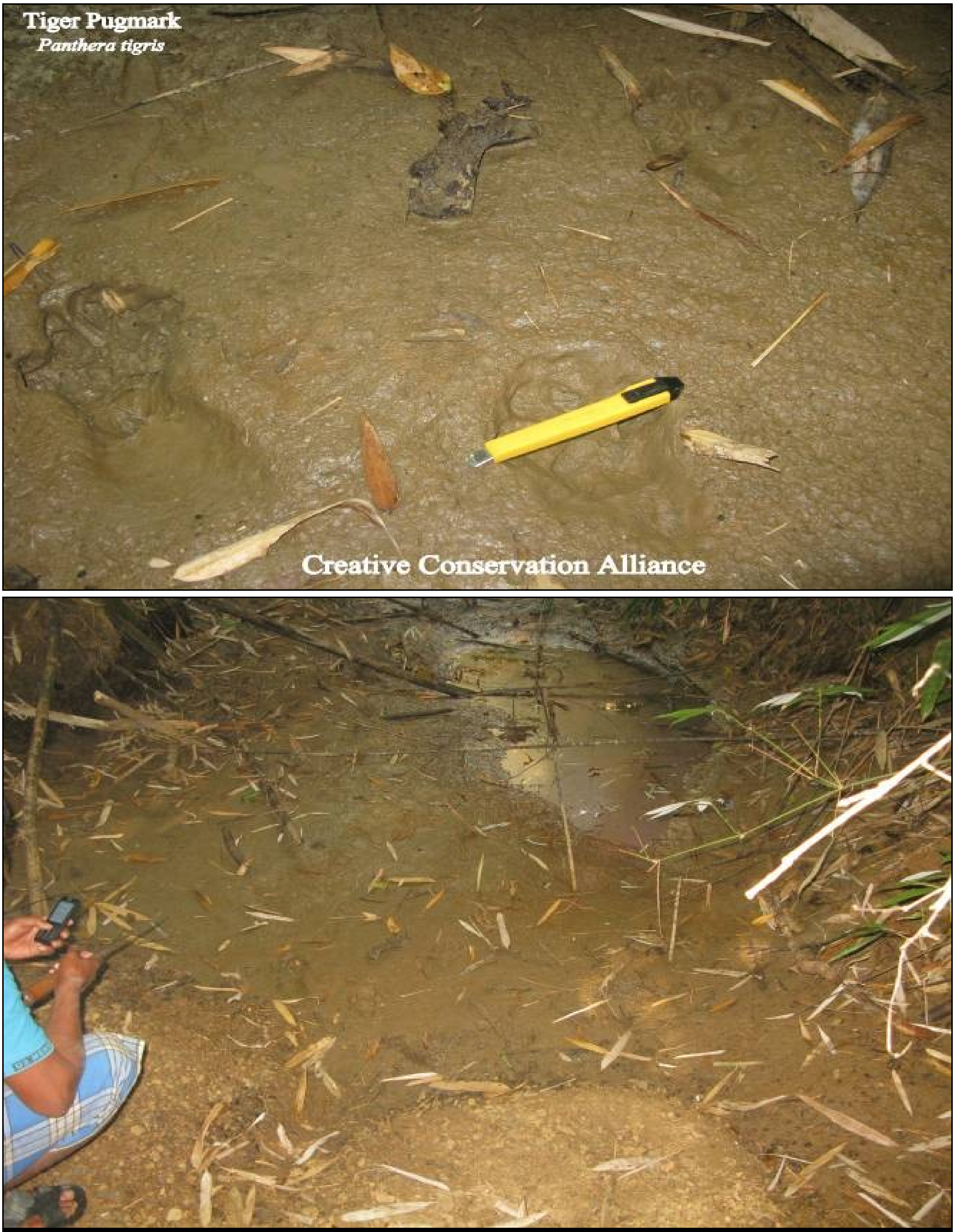

Figure 7: The creek where the tiger pug marks were found

We also recorded the presence of key tiger prey species such as Sambar, wild boar, and barking deer. These species are found in both primary forest and degraded habitat. The locals mentioned that they have seen wild boar herds of over 200 individuals. Sambars are relatively rare, but do occur throughout the SRF. Barking deer and wild boar appeared to be very common in the SRF and throughout the Sangu-Matamuhuri Valley.

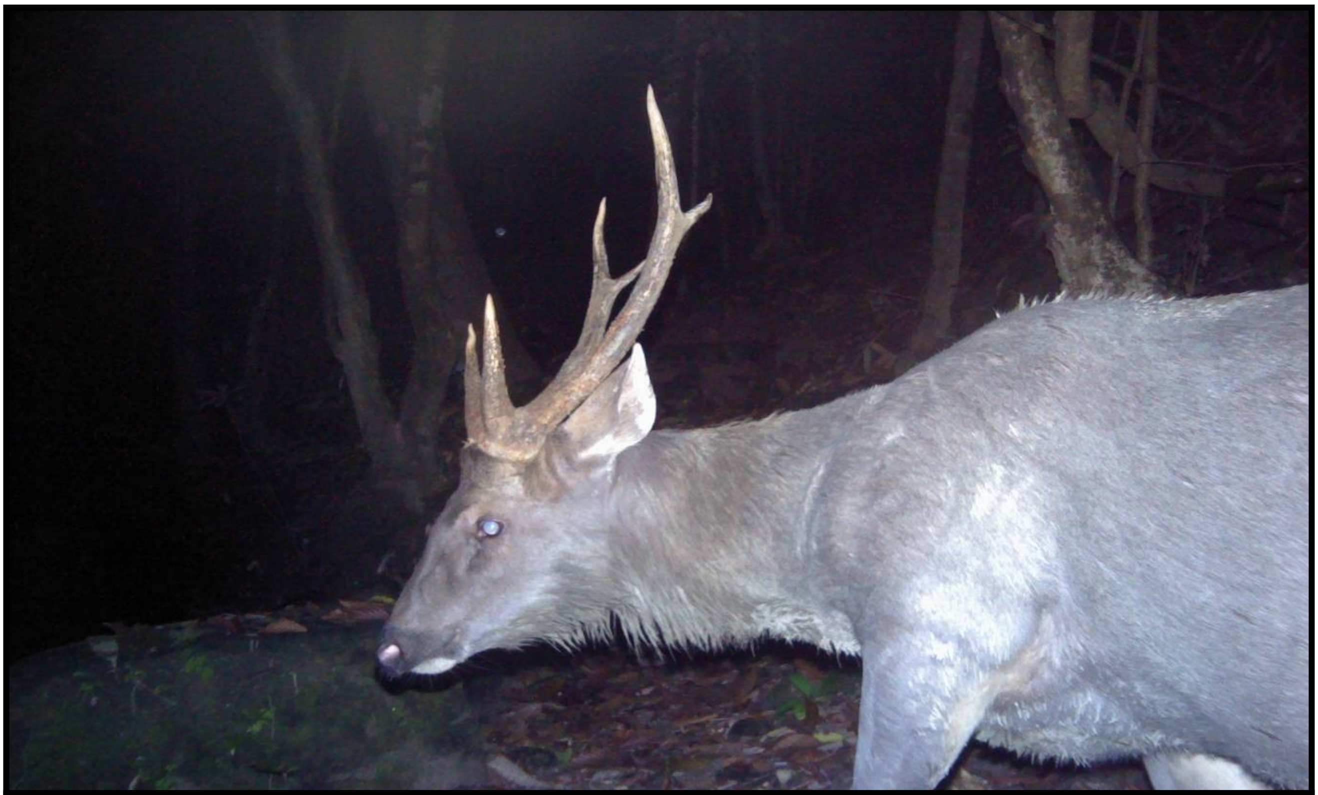

Figure 8: A male sambar

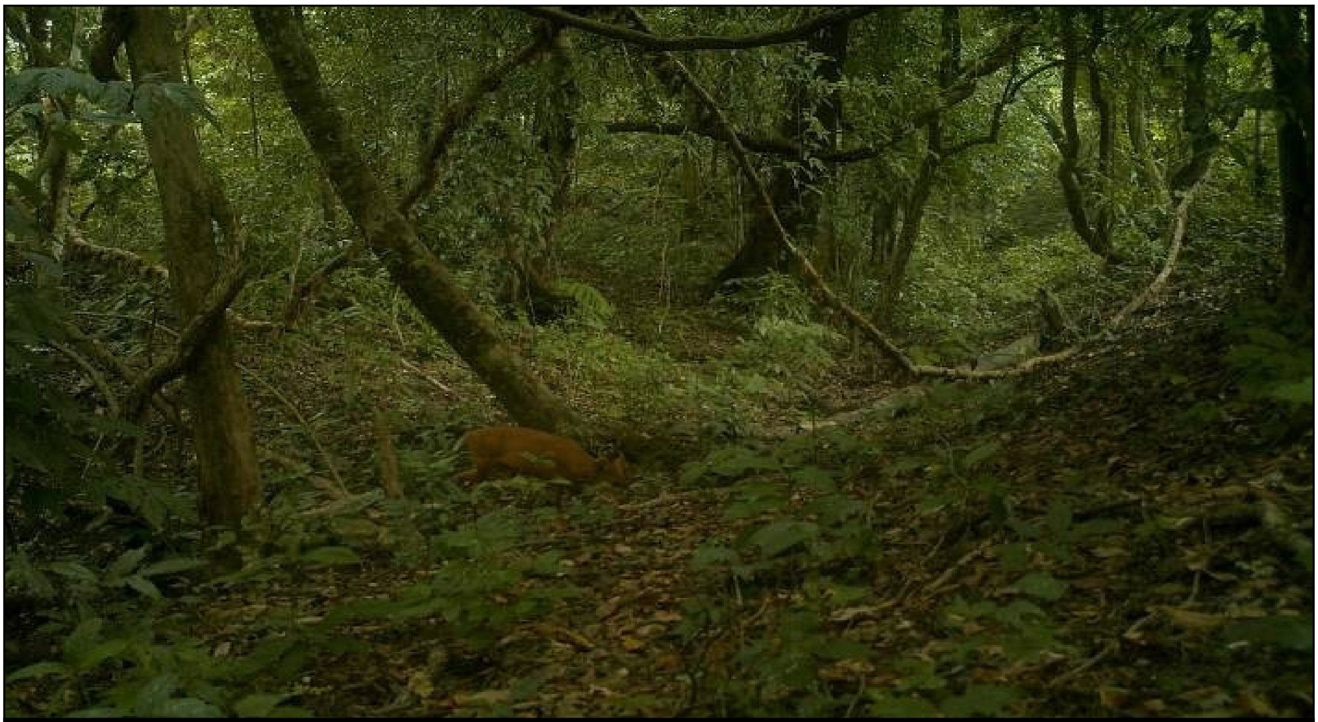

Figure 9: Barking deer

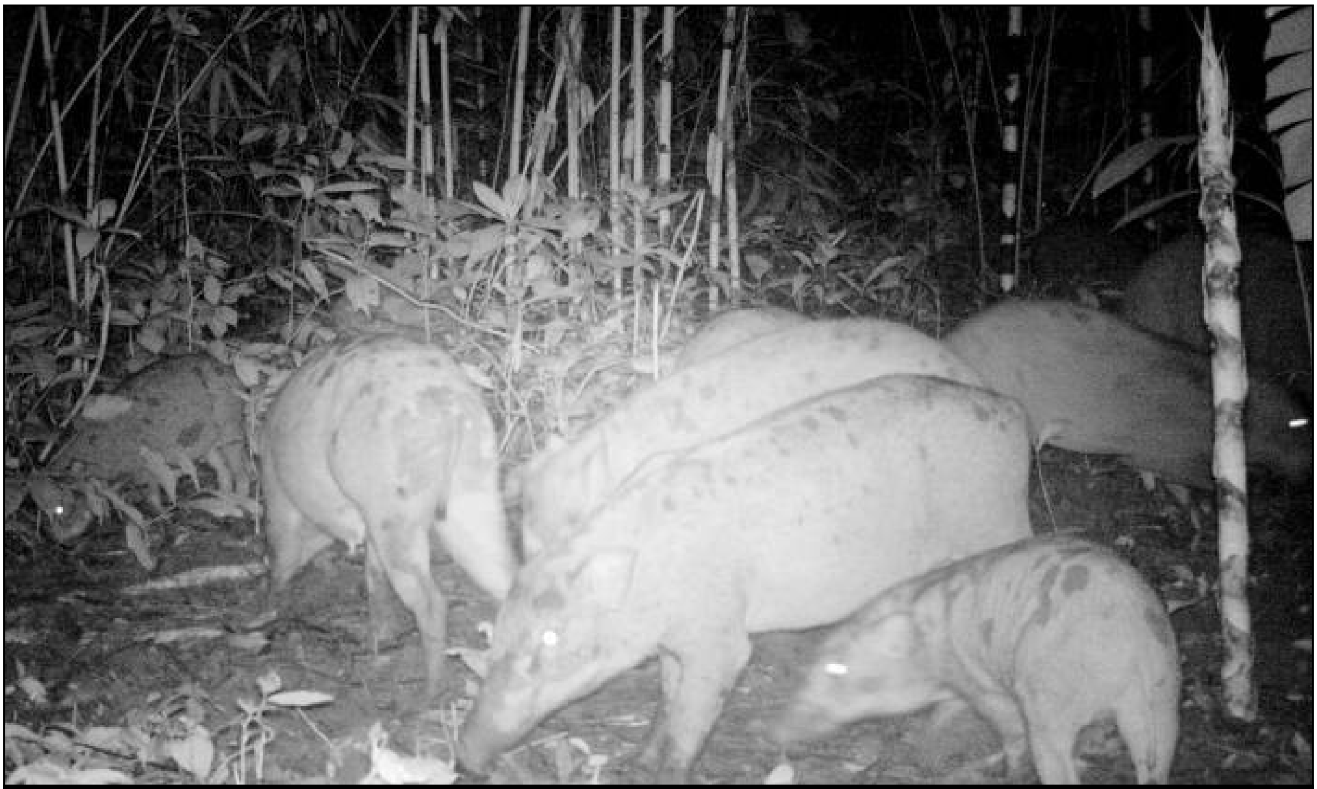

Figure 10: Wild boar

Apart from tigers, we have documented the presence of six other wild cat species within the SRF. We have found camera trap images and recently hunted skins of leopard (*Panthera pardus*), clouded leopard (*Neofelis nebulosa*), marbled cat (*Pardofelis marmorata*), Asiatic golden cat (*Catopuma temminckii*) and leopard cat (*Prionailurus bengalensis*). Wild cat species are opportunistically hunted by locals primarily for subsistence. Hunters from neighboring Myanmar are also reported to visit the SRF occasionally to trap for tigers and other wild cats. We did not record fishing cat (*Prionailurus viverrinus*) during our study period but they are known to occur within the SRF (S. Chakma, Pers. Comm.). The smallest of the locally occurring cat species, the jungle cat (*Felis chaus*), probably occurs closer to urban areas and human settlements.

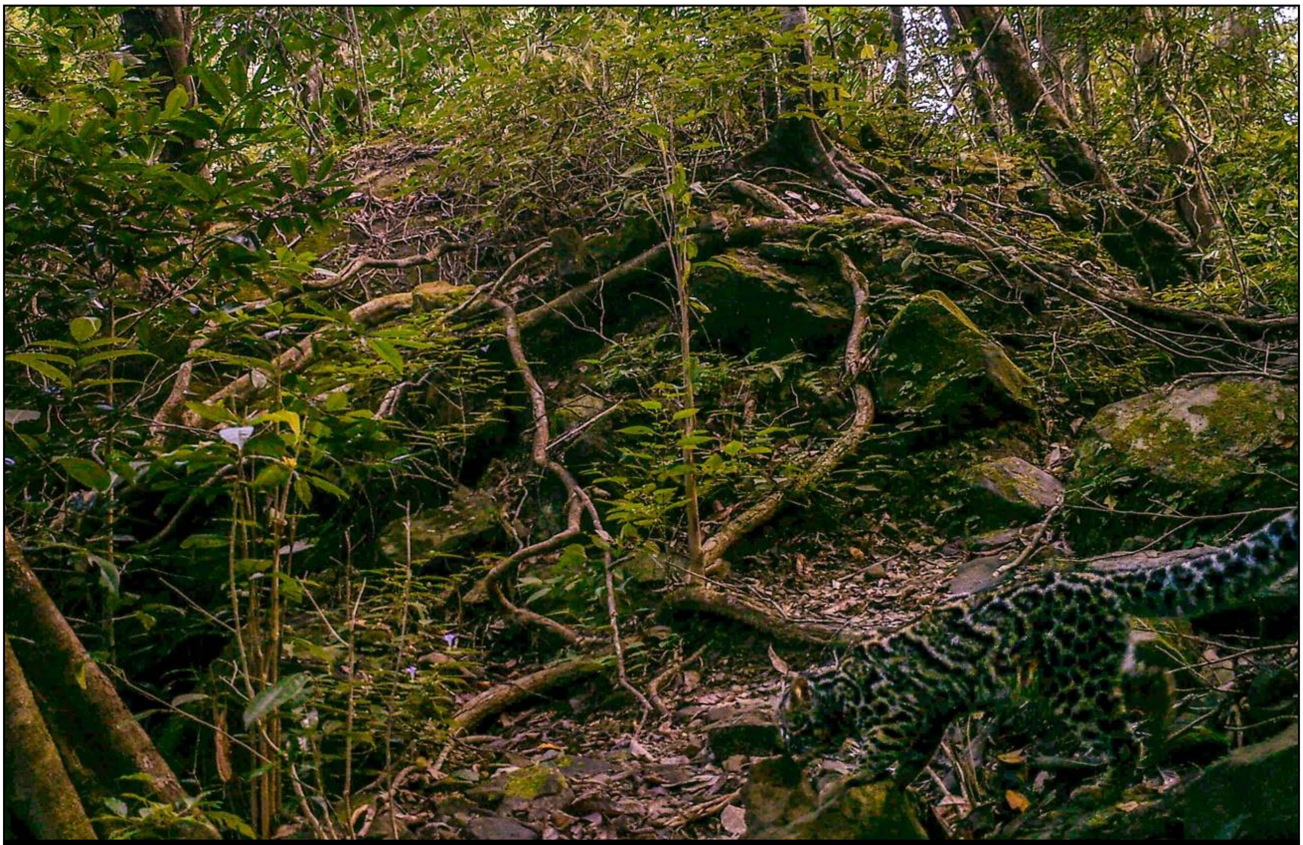

Figure 11: Marbled cat

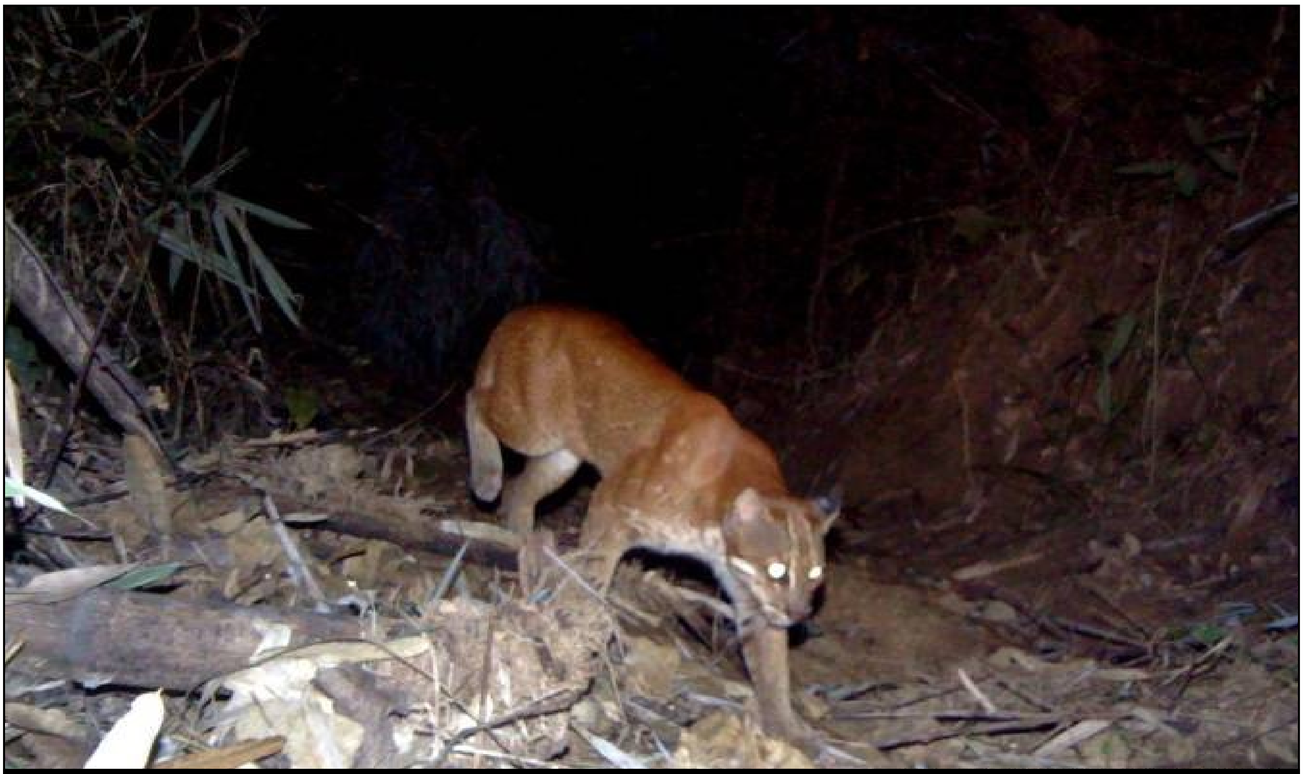

Figure 12: Asiatic golden cat

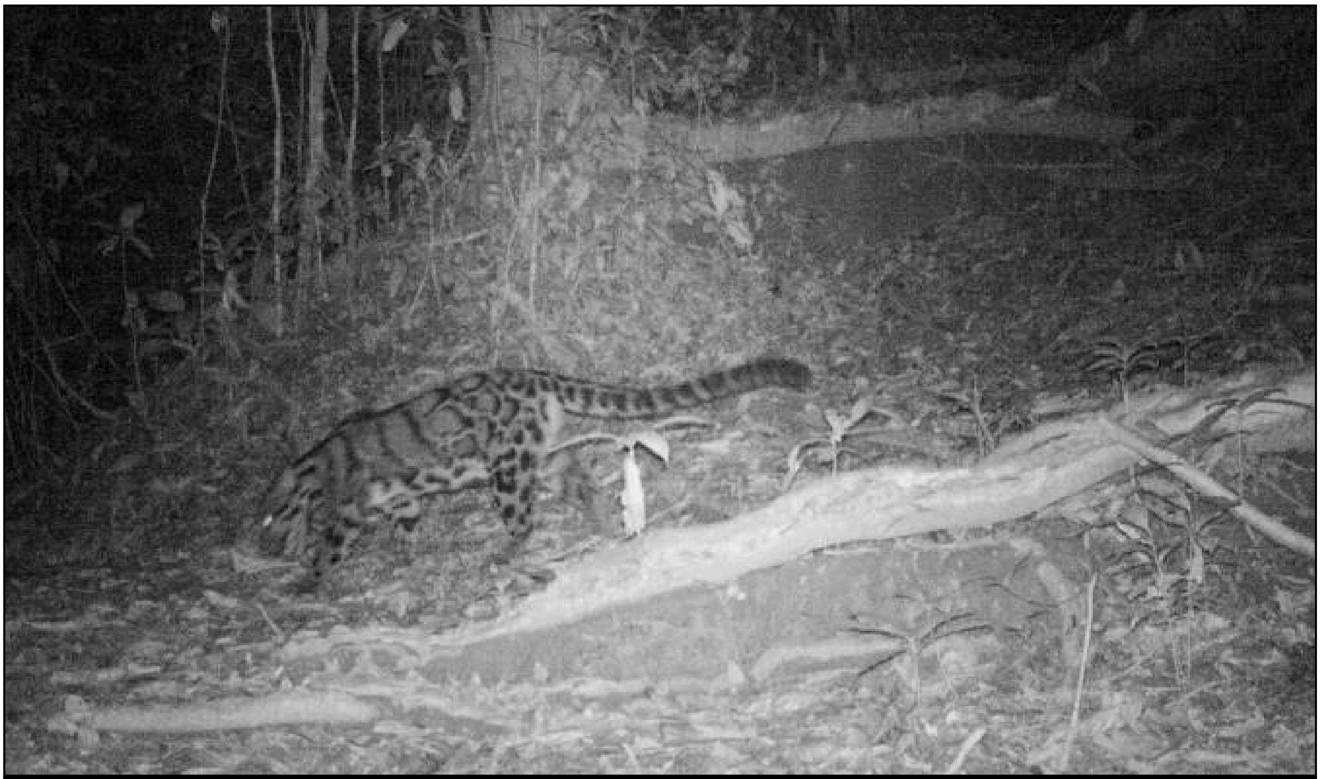

Figure 13: Clouded leopard

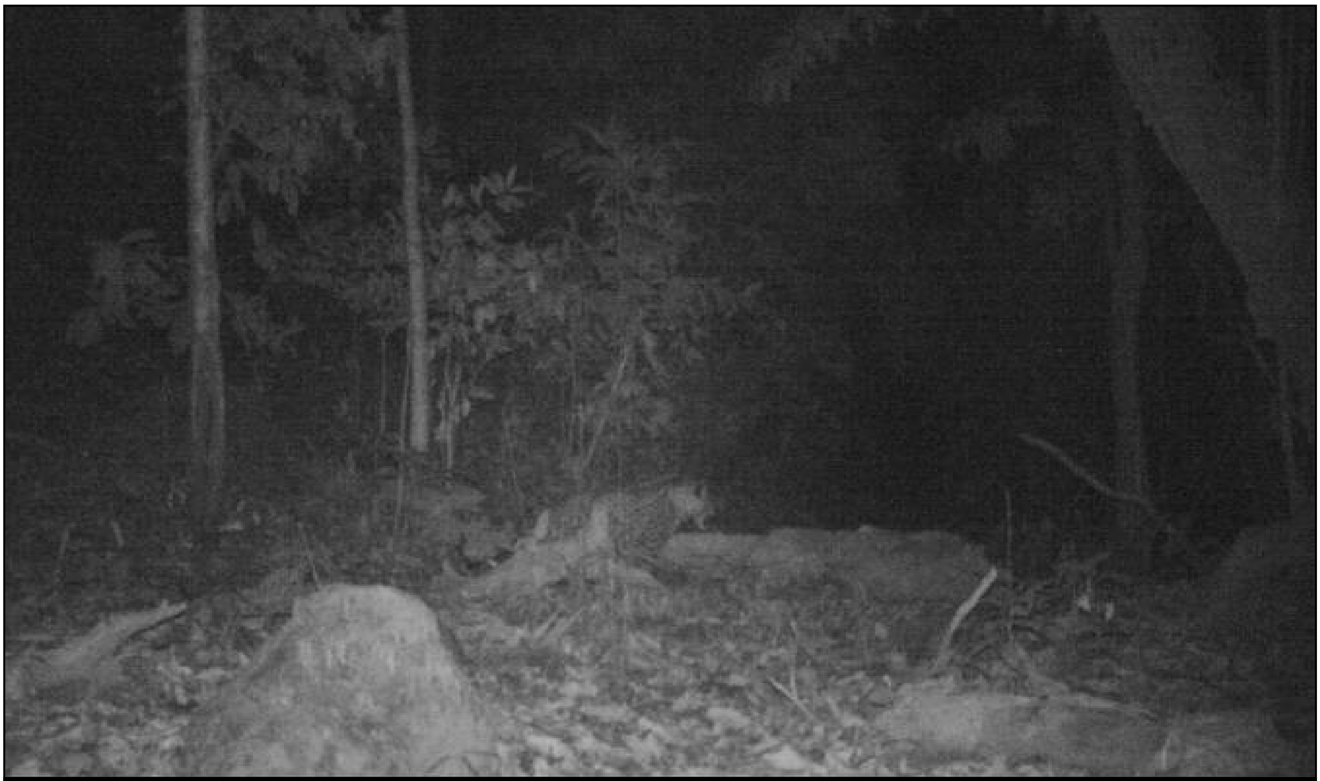

Figure 14: Leopard

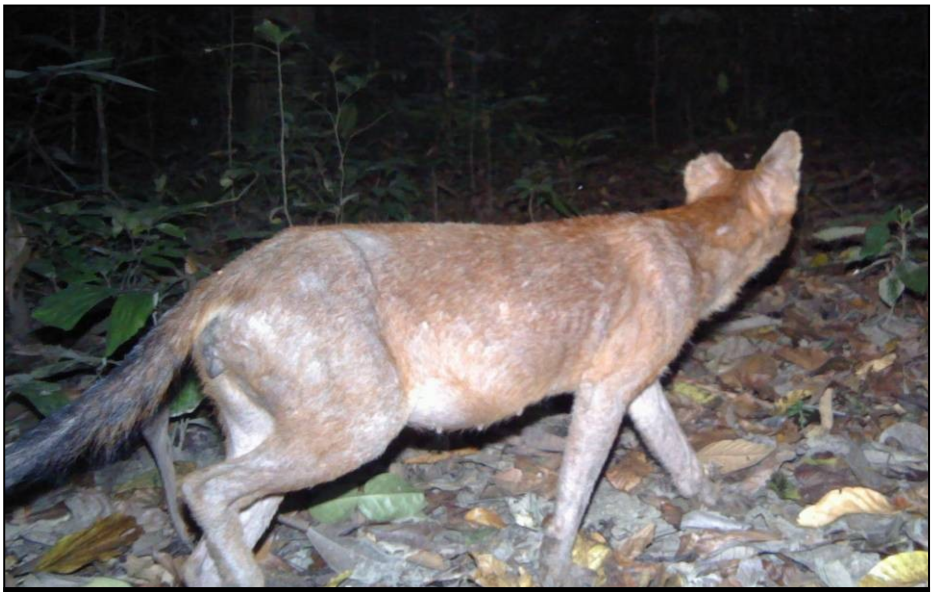
Figure 15: Asiatic wild dog/Dhole

Among the wild canids, we have recorded the globally endangered Asiatic Wild Dog/Dhole *(Cuon alpinus)* and golden jackals (*Canis aureus*) in the SRF and adjacent areas. Dholes were found to occur throughout the SRF and the adjacent areas. We have recorded at least five different dholes in our camera traps including a pregnant individual. The locals mentioned that dholes are fairly common in thearea, while jackals appeared to be very rare in the area and have accounted for only one recent hunting record outside of the SRF.

We also have rediscovered populations of wild gaur (*Bos gaurus*) in SRF. Gaur were declared extinct from Bangladesh according to IUCN (2000) Red List. We have recorded at least three different herds of guar in our camera trap images during the study. The locals approximated that 60-100 gaur are found in this area.

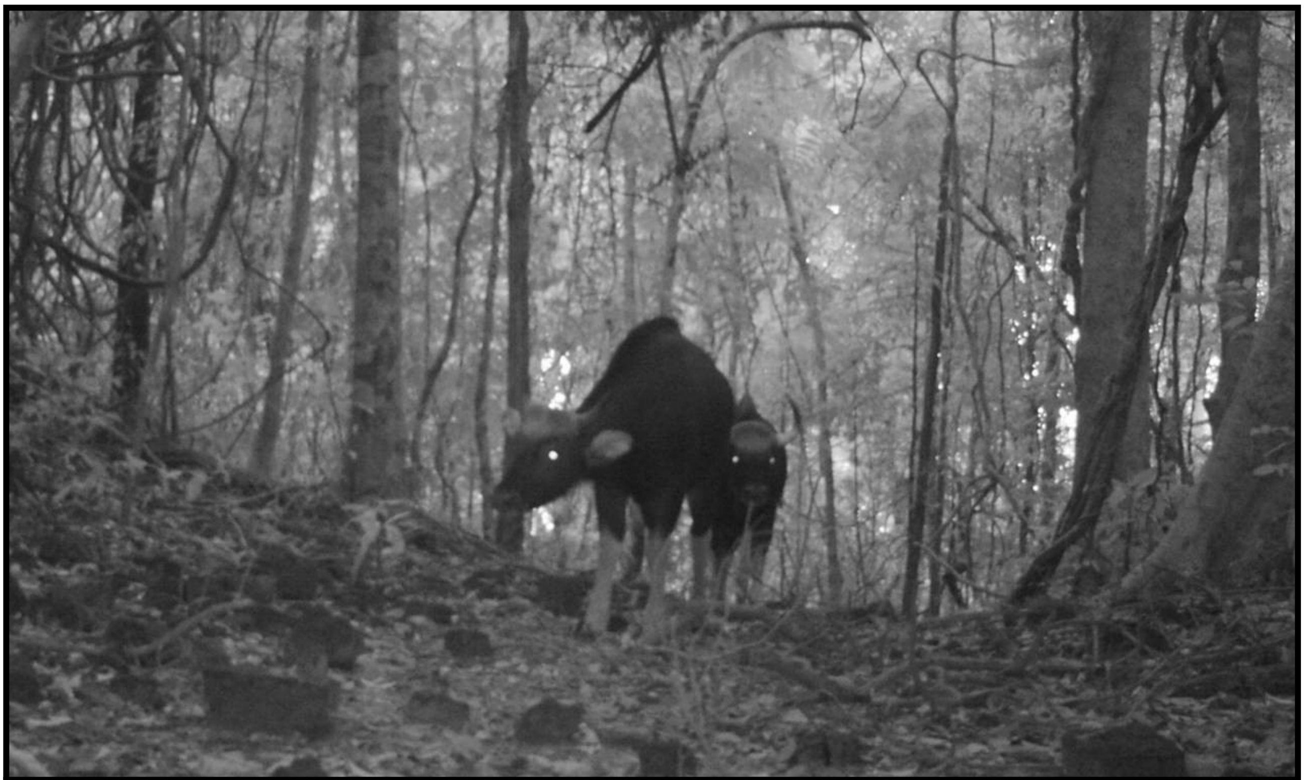
Figure 16: Gaur

We have also recorded several resident herds of Asian elephants *(Elephas maximus*) in the SRF. According to the locals, about 38 elephants live in this area. They also mentioned there has been a sharp decline in the population of elephants; poachers from Myanmar are known to visit this area to hunt wild elephants for ivory and meat.

We recorded six species of primates in SRF, including two globally endangered species- Western hoolock gibbon (*Hoolock hoolock*) and Phayre's leaf monkey (*Trachypithecus phayrei*). The gibbon population in the SRF is probably one of the largest remaining populations in Bangladesh. Arboreal species, such as hoolock gibbon, capped langur, and Phayre’s leaf monkey have been extirpated from most regions within the Bandarban; their last stronghold is in the SRF. Pig-tailed macaque (*Macaca leonina*) and Rhesus macaque (*Macaca mulatta*) are found in degraded areas adjacent to SRF as well. And among the meso-predators, we have recorded masked palm civet (*Paguma lavarta*), large Indian civet (*Viverra zibetha)*, and binturong *(Arctictis binturong*) – a globally endangered mammal.

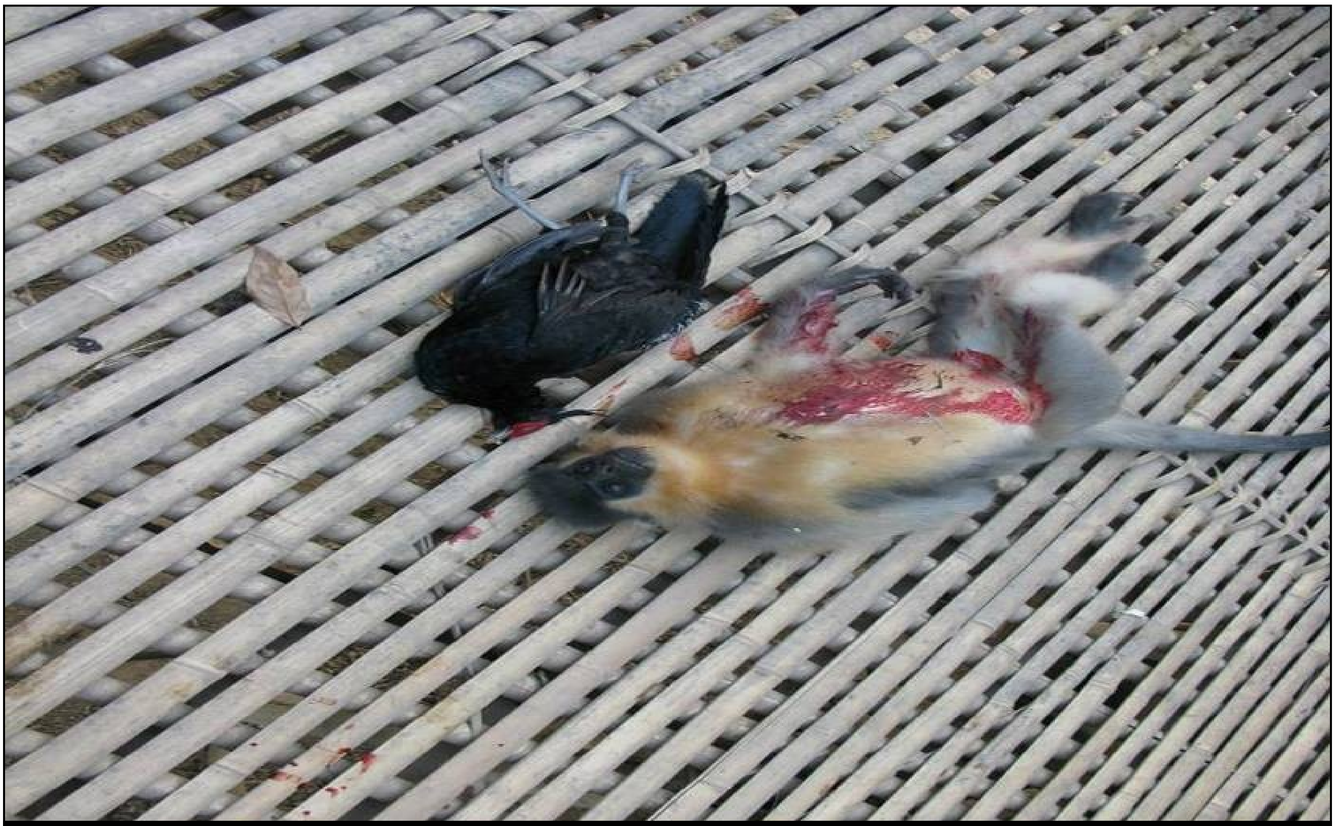

Figure 17 : Capped langur

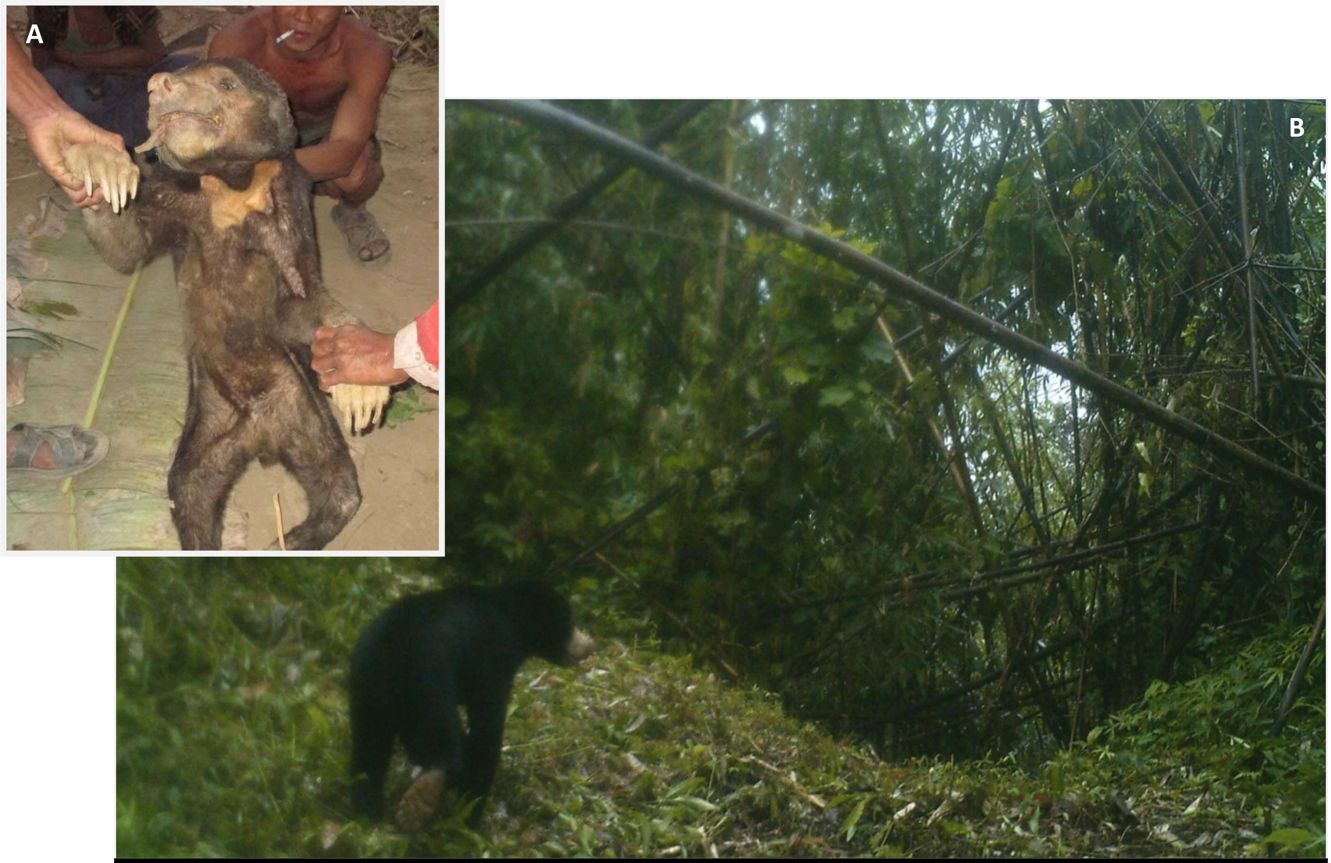

Figure 18: Sun bear

Two species of bears, the Asiatic black bear (*Ursus thibetanus*) and sun bear (*Helarctos malaynus*), were recorded in the SRF. A previous study mentioned that resident populations of sun bear do not occur in Bangladesh (Islam et al. 2013). We have captured camera trap photos of at least three different sun bears and found remains of recently hunted specimens in three different villages. This evidence indicates that resident populations of sun bear likely do occur in Bangladesh.

Both species of bears are found in primary and degraded patches of secondary forest. The local people often hunt bears during the jhum harvest season when the bears enter the fields to feed on crops.

Among rodents, we recorded two species of porcupines- Malayan porcupine (*Hystrix brachyura*) and Asiatic brush-tailed porcupine (*Atherurus macrourus*). Both species of porcupines appeared to be fairly common throughout the area and were found in both natural and degraded habitats. Two species of flying squirrels were recorded during the study – the common giant flying squirrel (*Petaurista petaurista*) and Hodgson's giant flying squirrel (*Petaurista magnificus*). Flying squirrels are nocturnal and are seldom seen during the day.

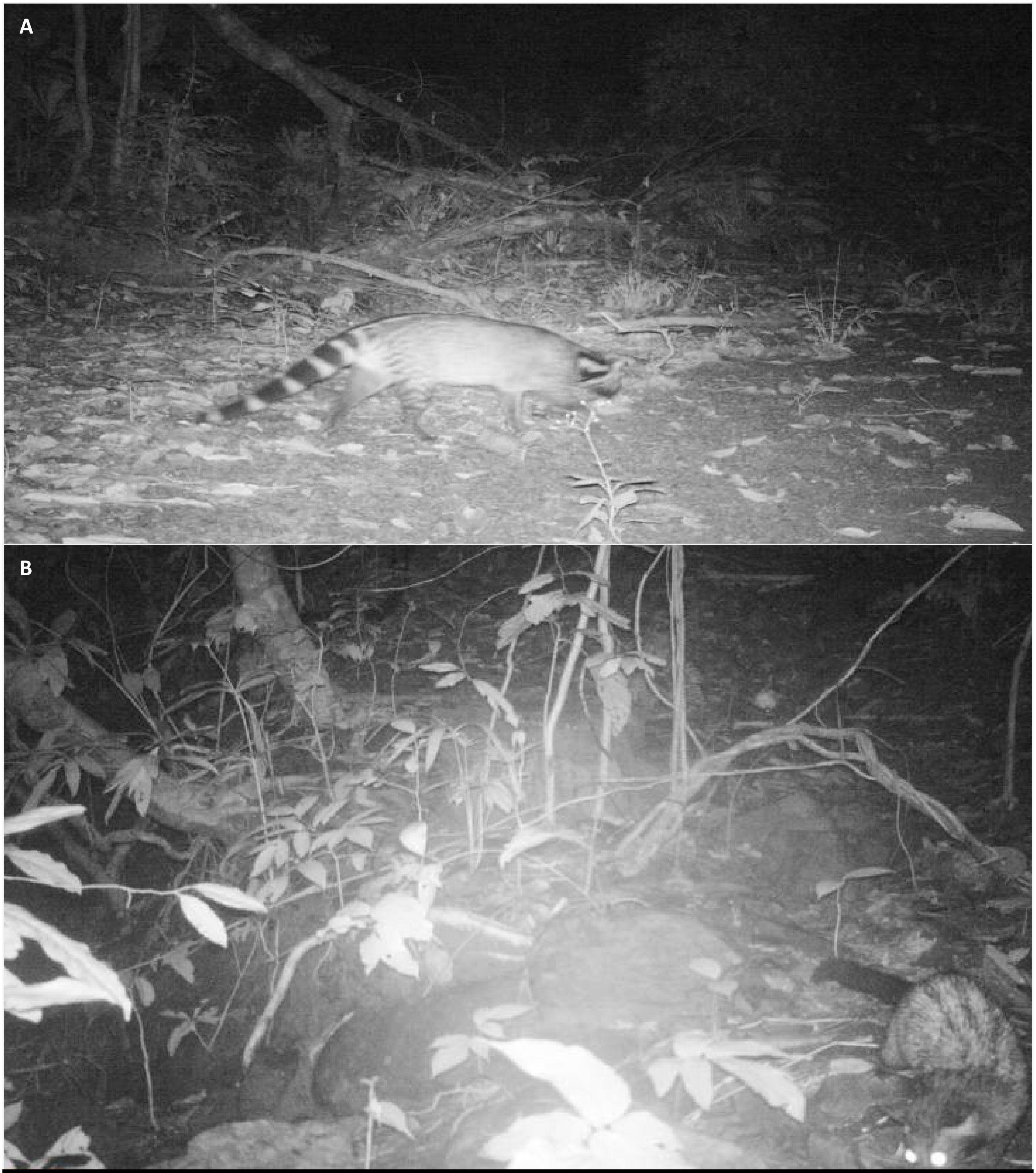

Figure 19: A. Large Indian civet; B. Masked palm civet

The critically endangered Chinese pangolin (*Manis pentadactyla*) is recorded from the region, however, the locals mentioned that pangolins are probably extirpated or are very rare due to the targeted poaching for their scales. Locals also reported that otters are also extirpated from the area. All other recorded mammals are listed below in the Appendix.

Notes on extinct megafauna:

The Mro people claim that Banteng never occurred in this area but that wild water buffalo still exists in the area. It can be safely assumed that rhinoceros no longer exist in this part of the CHT. A 90-year-old Mro man mentioned that he encountered a rhinoceros 70 years ago in the Sangu-Matamuhiri Valley.

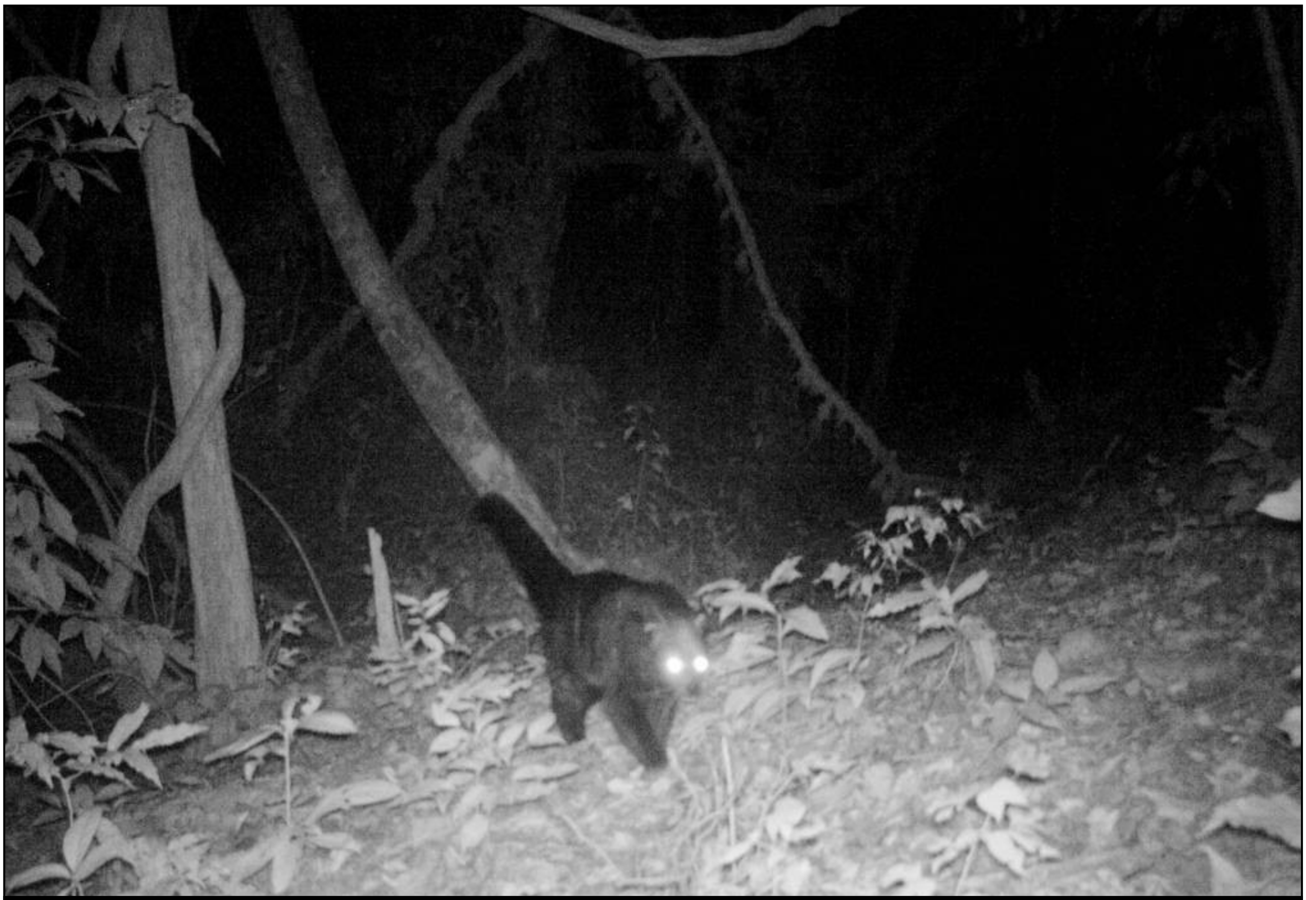

Figure 20: Binturong

6.2. Birds

While 200 + bird species may occur in the SRF and adjacent areas (M. Khan, Pers. Comm.), providing a detailed checklist of birds was beyond the scope of this report. Herein, we present information on a few notable species only.

Red junglefowl (*Gallus gallus*) and Kalij pheasants (*Lophura leucomelanos*) appeared to be very common throughout the Sangu-Matamuhuri Valley. Resident populations of grey peacock pheasant (*Polyplectron bicalcaratum*), and three species of hornbills: great hornbill (*Buceros bicornis*), wreathed hornbill (*Rhyticeros undulates*) and Oriental-pied hornbills (*Anthracoceros albirostris*) were found in the SRF, primarily in the Chimbook Hill area, west of the Sangu River. Some locals also mentioned that green peacocks still also occur in the area, however, we have yet to find any direct evidence of their presence.

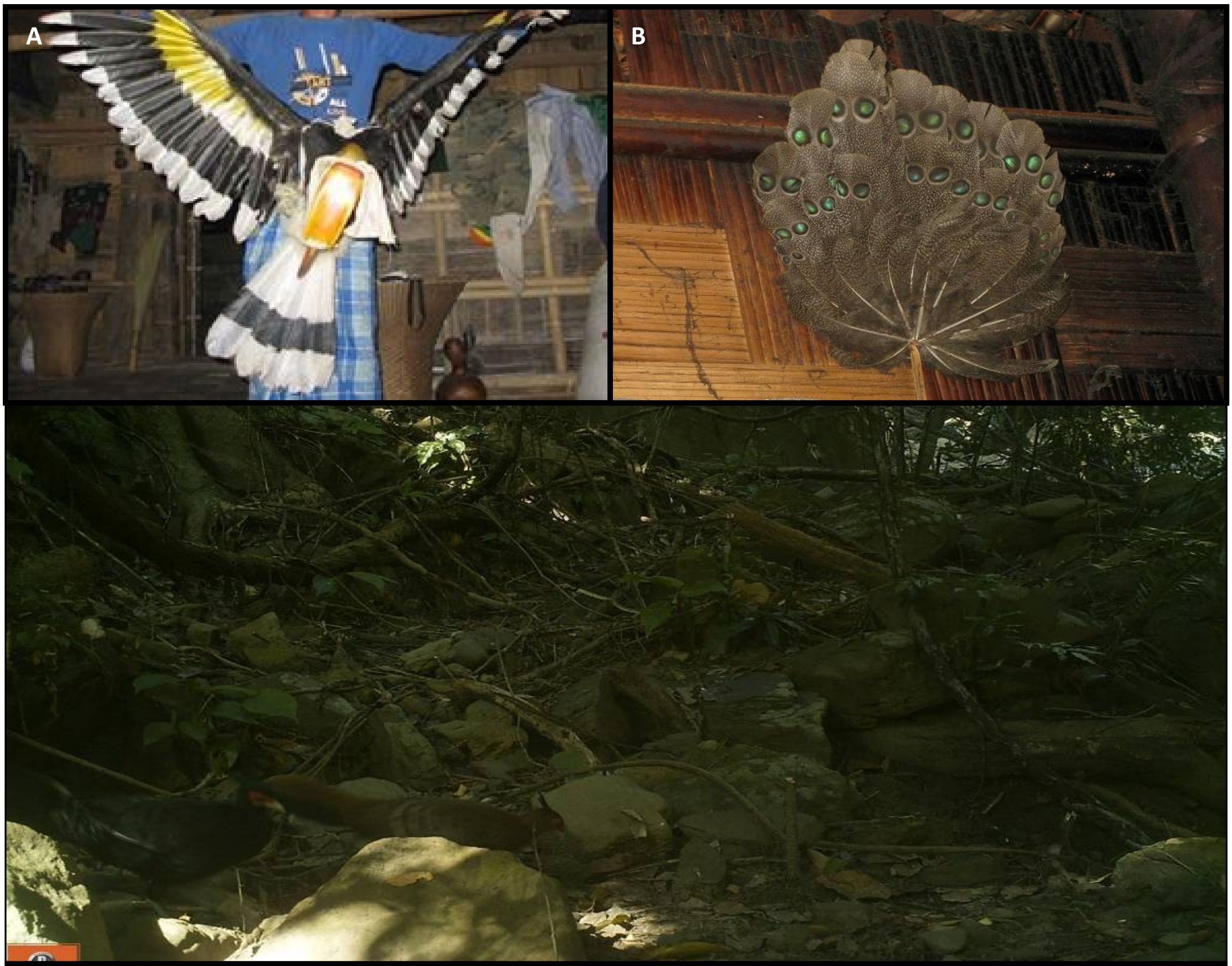

Figure 21: A. Great hornbill ; B. Grey-peacock pheasant ; C. Kalij pheasant

6.3. Reptiles and Amphibians

Thus far, we have recorded 46 species of reptiles and 19 species of amphibians. This checklist is preliminary and many more species are waiting to be discovered, some are potentially new to science. We have recorded seven species of globally threatened turtles and tortoises in the area, including the Arakan forest turtle (*Heosemys depressa*), Asian brown tortoise (*Manouria emys*), keeled box turtle (*Cuora mouhotii*), elongated tortoise (*Indotestudo elongata*), Sylhet roofed turtle (*Pangshura sylhtensis*), leaf turtle (*Cyclemys gemeli*), and Malayan softshell turtle (*Amyda ornata*). The critically endangered Arakan forest turtle and the endangered keeled box turtle were recorded from Bangladesh for the first time. The findings of the globally endangered Asian brown tortoise and Sylhet roofed turtle in the SRF opens up new doors for the conservation of these two rare turtles as both of these species were previously considered extirpated from Bangladesh.

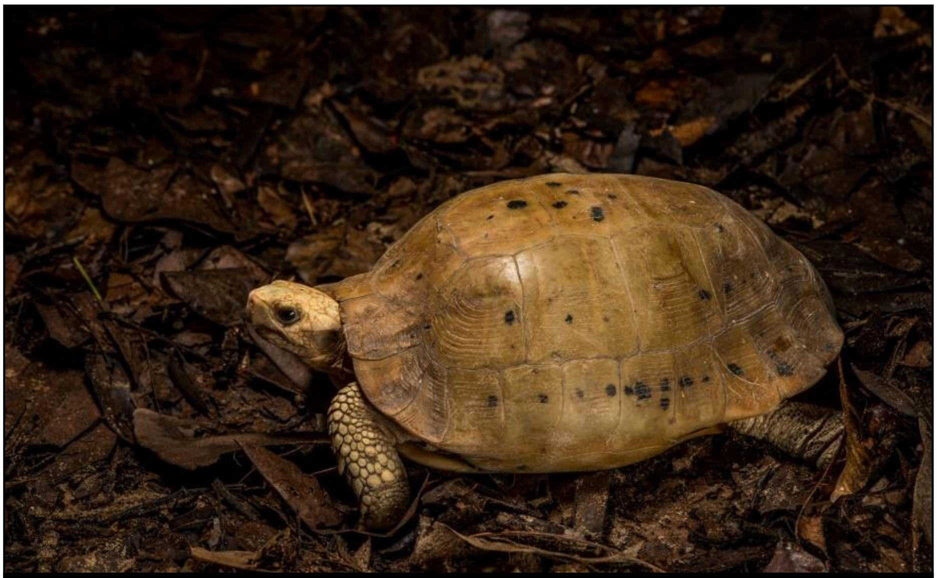

Figure 22: Elongated tortoise

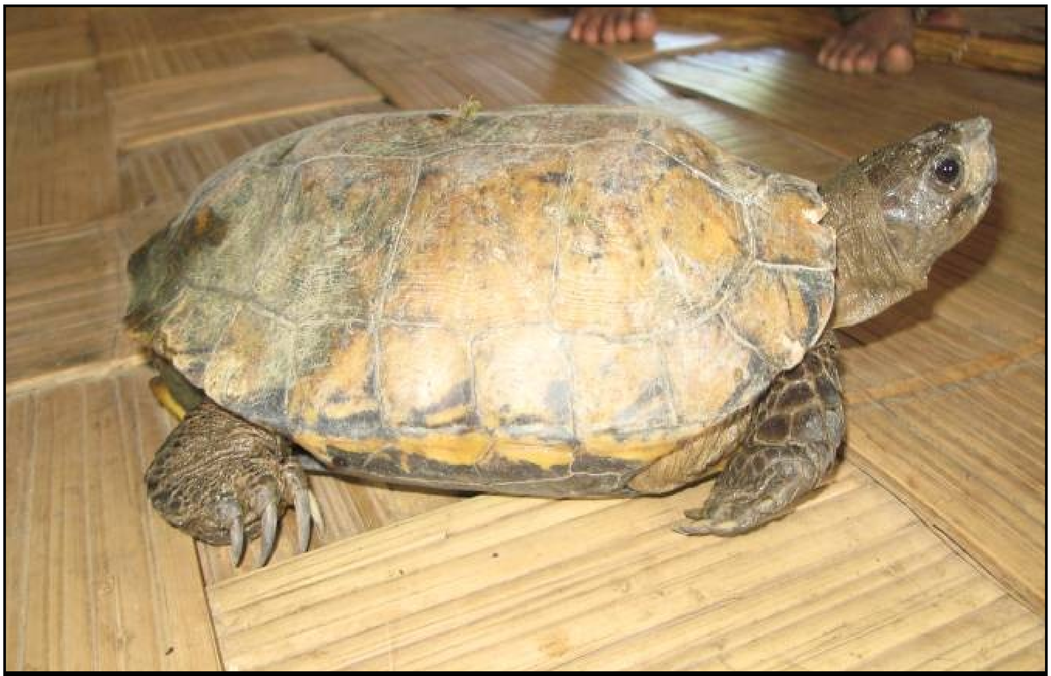

Figure 23: Arakan forest turtle

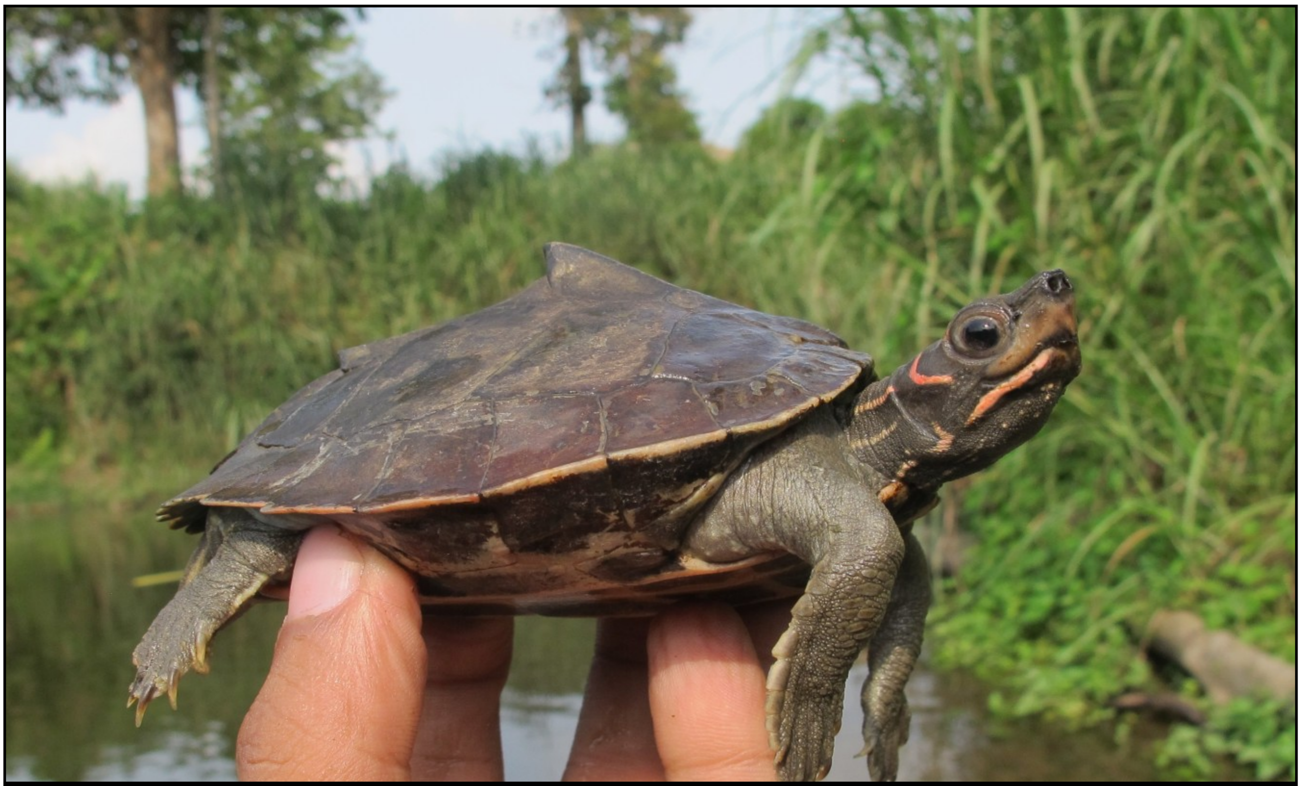

Figure 24: Assam roofed turtle

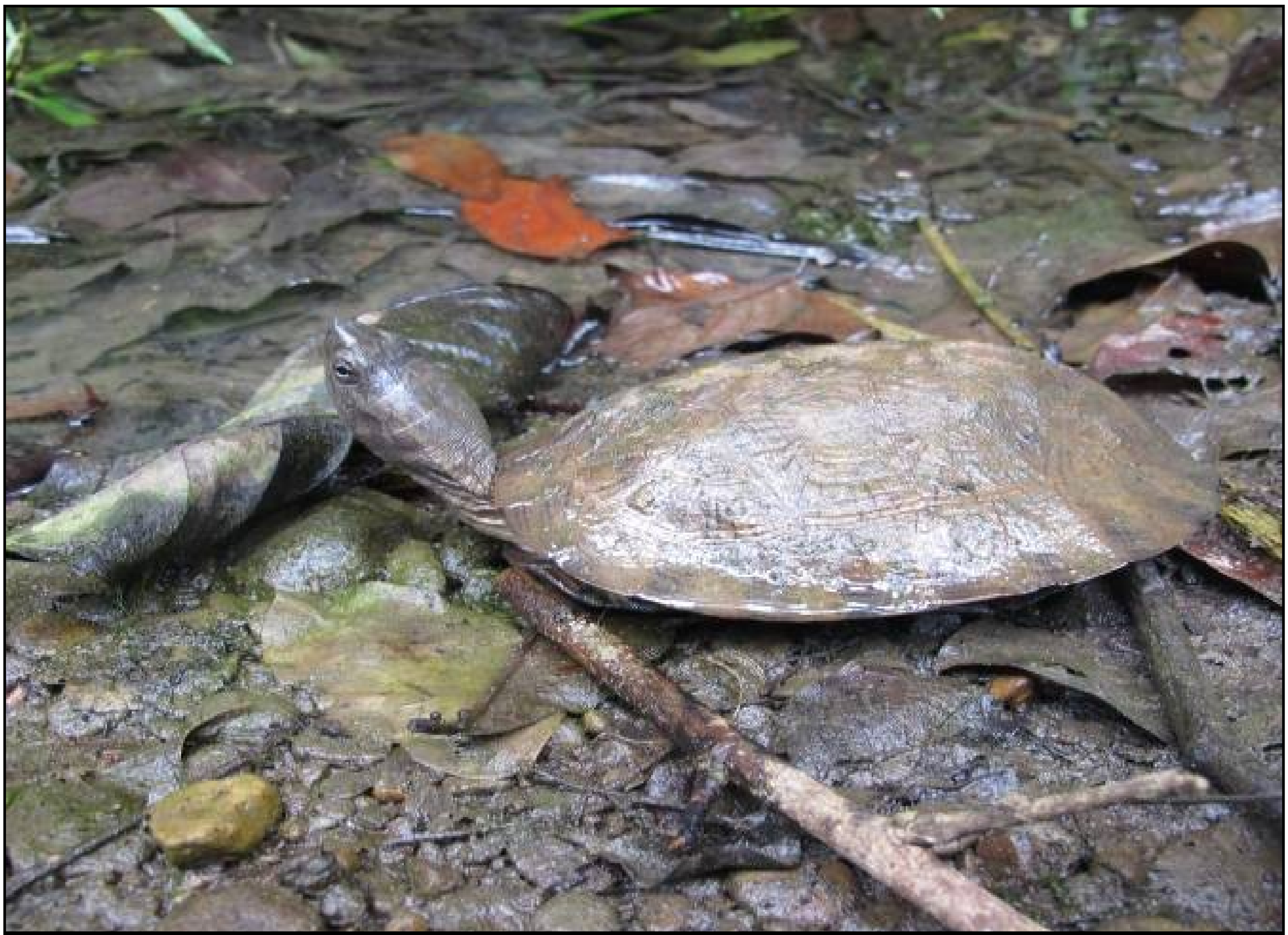

Figure 25: Asian leaf turtle

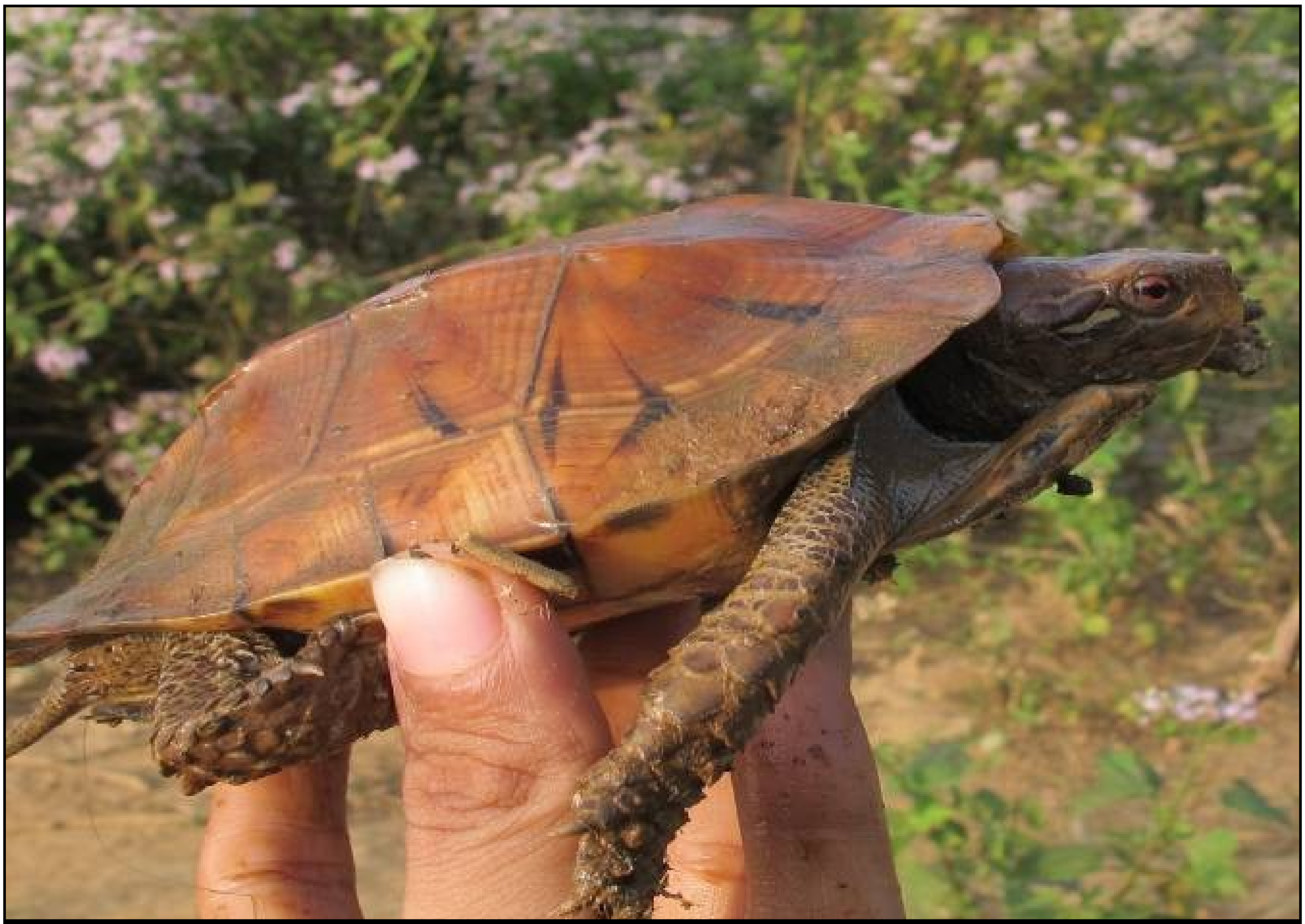

Figure 26: Keeled box turtle

Two globally threatened snake species, the Burmese python (*Python bivittatus*) and king cobra (*Ophiophagus hannah*) are recorded from the SRF. The world's longest snake, the reticulated python (*Malayopython reticulatus*) was also recorded from the SRF. Both species of pythons and the king cobra are reported to be fairly common in the area, and are found in both primary and degraded forest patches. Three other snake species were also recorded from Bangladesh for the first time.

The globally threatened Indian flying frog (*Pterorana khare*) and Asian caecilian (*Ichthyophis* sp.) were recorded during the study for the first time from Bangladesh.

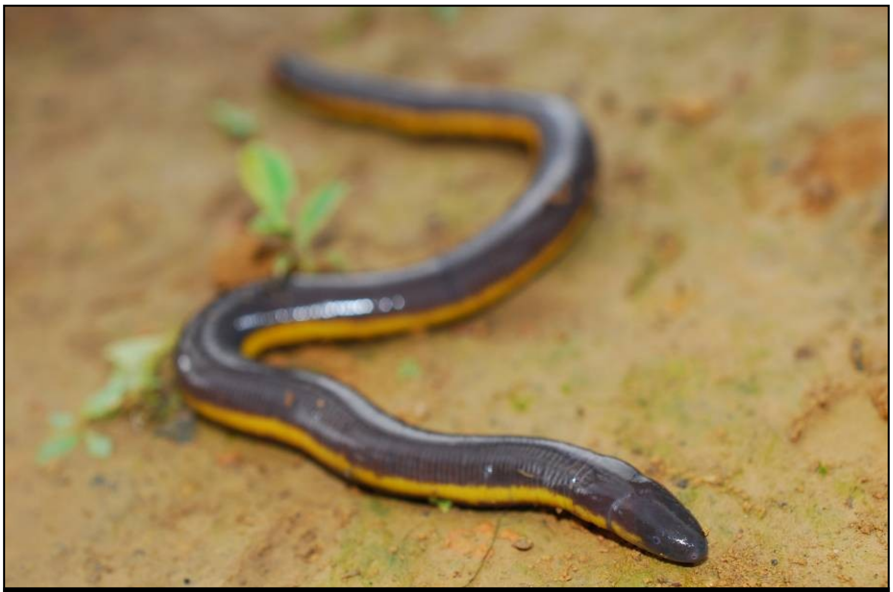

Figure 27: Asian caecilian

6.4. The People

Until very recently, there were no permanent settlements in the SRF. The majority of the settlements within the SRF were established within the last decade. The recent human influx is caused by the decreases in soil fertility and jhum production in most parts of the Bandarban forcing the tribal communities to migrate to the pristine areas of the SRF. There are 16 villages within the SRF; eleven of them are Mro villages, three Marma villages, and two Tripura villages. The approximate total population of the area is 1,632, with an average of 102 people in each village. The mean Mro village size in our study consists of 15 households (range: 5-24). The mean population of the village is 84 persons (range: 34-150; SE: 6.37); the mean aerial distance of the village to the nearest town is 22.65 km (range: 3.05-49.54 km; SE: 2.13).The tribes commonly keep cattle, goat, chicken, and pigs for festive occasions (Loffler 1961). We noted that almost every household has domesticated chickens and pigs, and some also cattle. The Mro do not

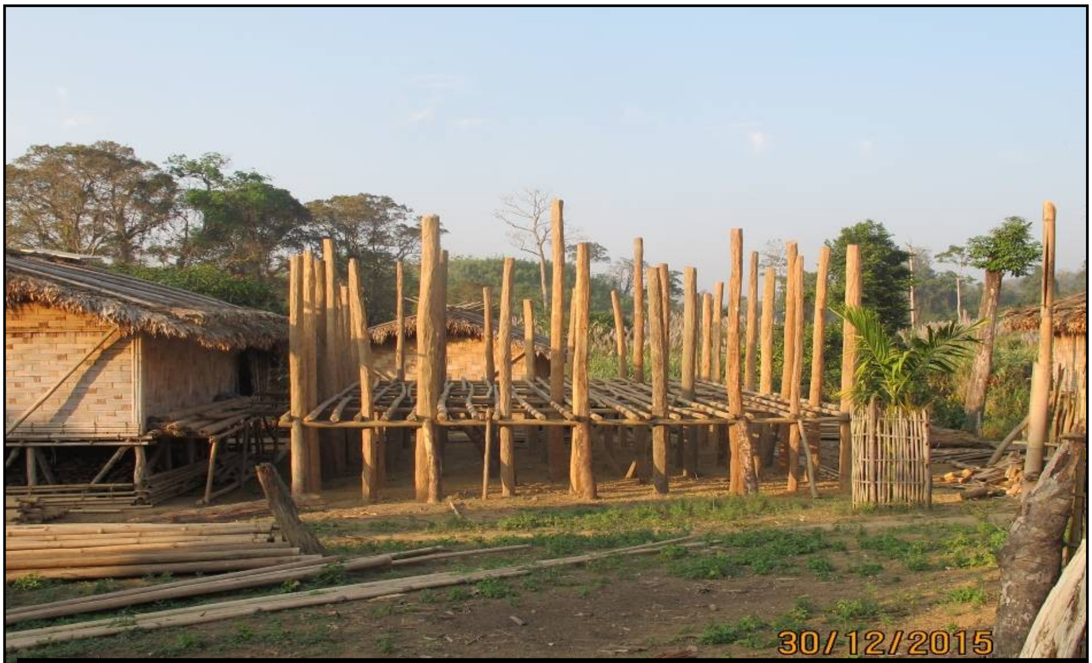

Figure 28: A Mro settlement

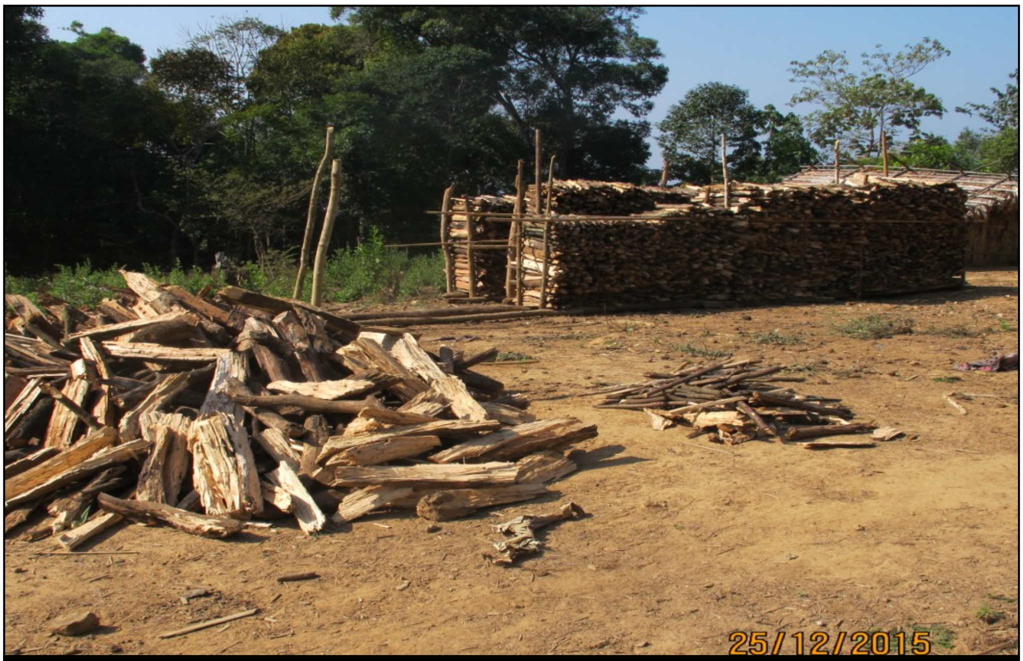

Figure 29: Firewood extraction for household use

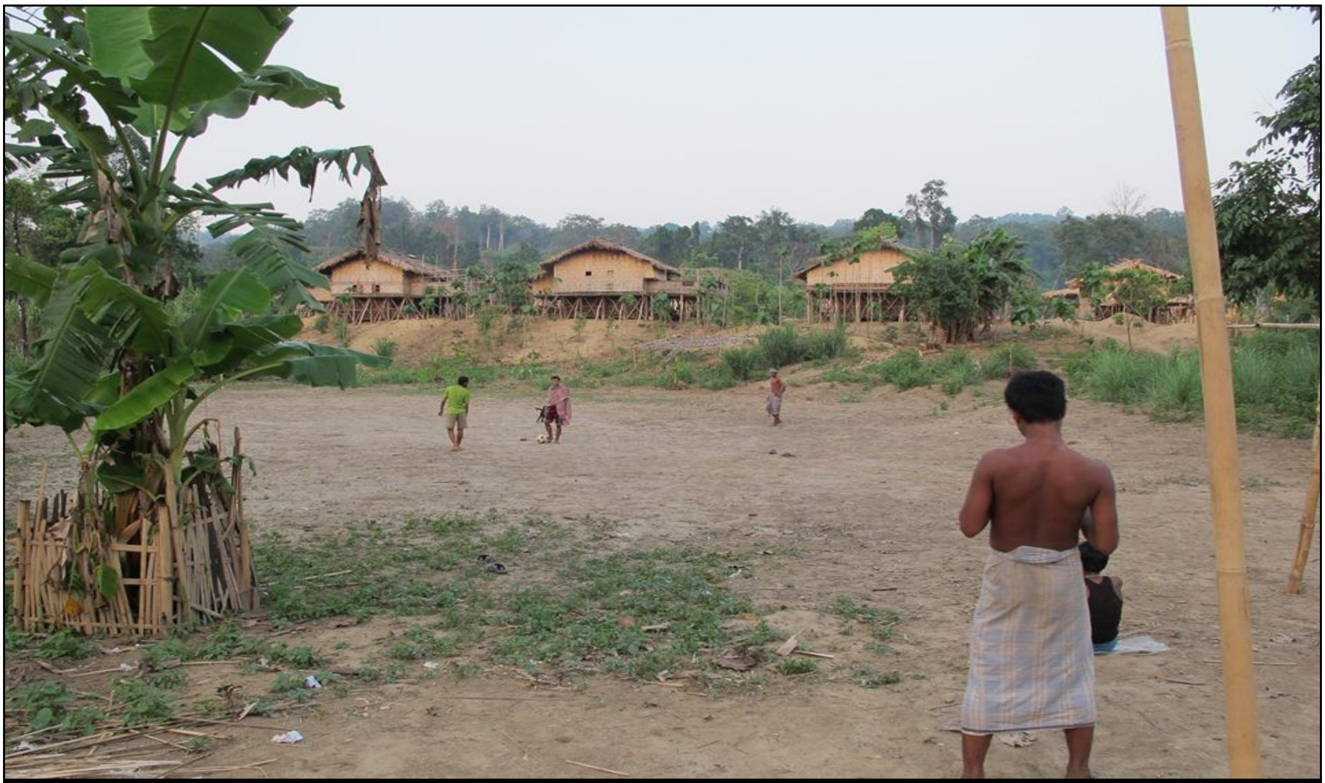

Figure 30: A Mro village

Commercially raise livestock; that which they have is usually free-ranging and is only occasionally supplementary fed. Cattle raised are usually sold to Bengali settlers or tribal traders for cash, or to consume for different festivities, such as "Chia-Shod-Poi." It seems the numbers of animals they keep are barely enough to provide a full dish for their festive occasions.

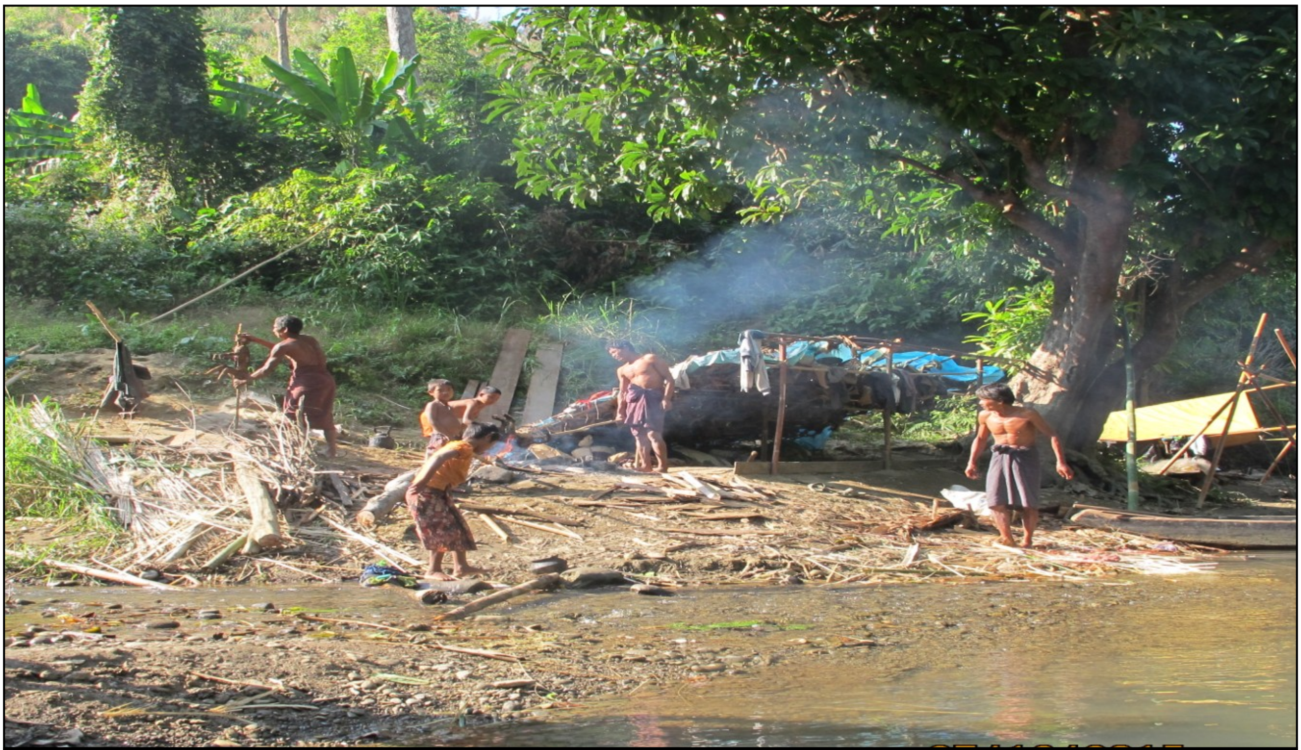

Figure 31: A temporary shelter of seasonal resource collector on the bank of the Sangu river, within the SRF

Their main vocation is swidden agriculture. *Jhum* fields are usually located within 3 km walking distance of a village. The Mro grow rice, their main staple and also pumpkin, squash, and other vegetables for domestic consumption, and cotton, ginger, and turmeric which are usually sold to Bengali traders for cash. Their diet also includes bamboo shoots, vegetables, supplemented by fish, crabs, and occasionally bush meat.

The men and women are very busy starting in February when they first clear a plot to burn, until October when they harvest their crops. During that time they do not seem to have enough time to go on hunting trips. They will then passively hunt and place traps around their fields and around their villages to catch crop-damaging meso-predators.

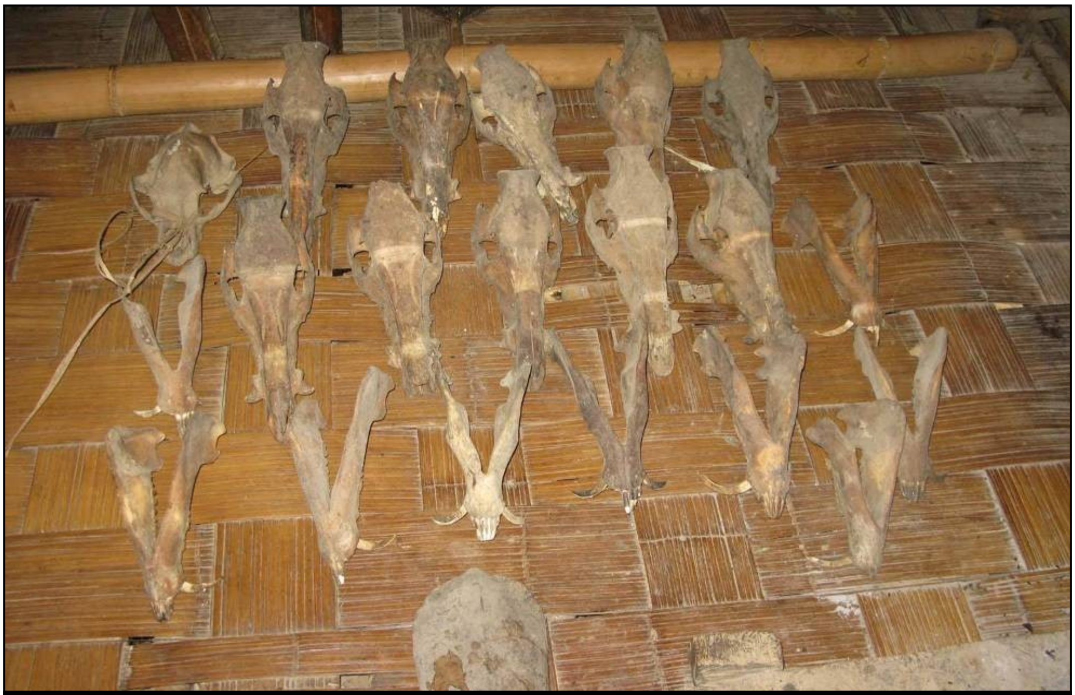

Figure 32: Wildlife trophies

The Mro are opportunistic subsistence hunters, primarily utilizing traps and guns. This may be regarded as a more reactive hunting approach by them, rather than a proactive one. Since guns have been used for >50 years they no longer rely as much on traditional hunting traps. The use of guns to hunt during the wet season decreases as that is the time that 1) they are very active weeding in their *jhum* fields; 2) due to rain and flash floods, moving and traveling about becomes difficult; and 3) it is difficult to keep gunpowder dry in the heavy rains. They use traps throughout the year, with a trapping peak in the monsoon (June to September), and with guns during the dry season after the crop harvest (November to February).

The Mro often hunt with dogs, typically capturing terrestrial turtles and tortoises. It did not appear that the dogs are trained specifically to hunt. And the skills of the dogs vary from individual to individual. We have seen dogs in every Mro village; however, not all dogs are used for hunting. Typically during hunts, one or two Mro hunters joined the dogs. The dogs roamed freely in the forest and barked if an animal was discovered. They are often used to hunt monitor lizards around *jhum* fields as well.

Large animals such as deer, large turtles, etc. that are captured, are distributed among the villagers. The person(s) who were responsible for its capture receive the larger shares.

The Mro women are not considered "hunters", but if they encounter wildlife while performing daily chores, such as encountering a turtle streamside while collecting water, they will bring it back for consumption. We have observed that children roam around villages with catapults and will kill any bird for home consumption.

6.5. Subsistence Hunting Practices

Most (n=60) species were hunted opportunistically for subsistence/domestic consumption, while less than 10% were hunted for commercial sale: for live animal trade e.g. hill myna (*Gracula religiosa*) and parakeets (*Psittacula* spp.); or for body parts e.g. Chinese pangolin (*Manis pentadactyla*) and Tokay gecko (*Gekko gecko*). Pangolins and geckos are targeted based on their body size, destined to be used in Chinese traditional medicines. Occasionally parts of animals hunted for subsistence are also traded, e.g. bear bile, canine teeth of cats, and plastrons of turtles also destined for the Chinese traditional medicine market.

The survey estimated that approximately 66 species of animals, including mammals (about 51%), reptiles (27%), birds (17%), and amphibians (5%) were documented as hunted species by the Mro. Based on villagers' information, it is found that in the recent past otters were heavily hunted due to the high demand of their skin, and the villagers are concerned that this species is already extirpated in the region. Pangolins were primarily hunted for scales which are used in traditional Chinese medicine and are now on the verge of extirpation from the area; and are now listed as Critically Endangered (Table 1).

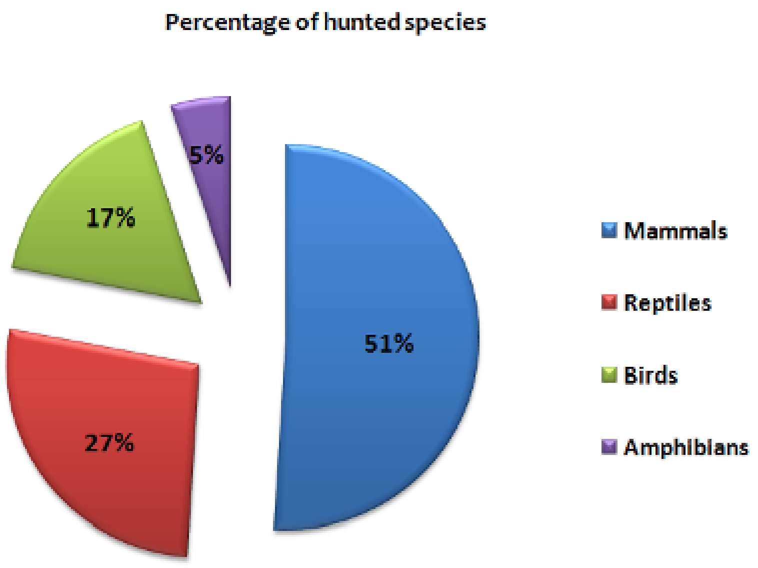

Figure 33 : Percentage of hunted species

Of the animals for which offtake numbers were obtained , many of the turtles and tortoises are listed as endangered, one of the hornbill species is listed as near threatened, and those other species taken in larger numbers such as barking deer, porcupines, and Bengal monitor are listed as of least concern.

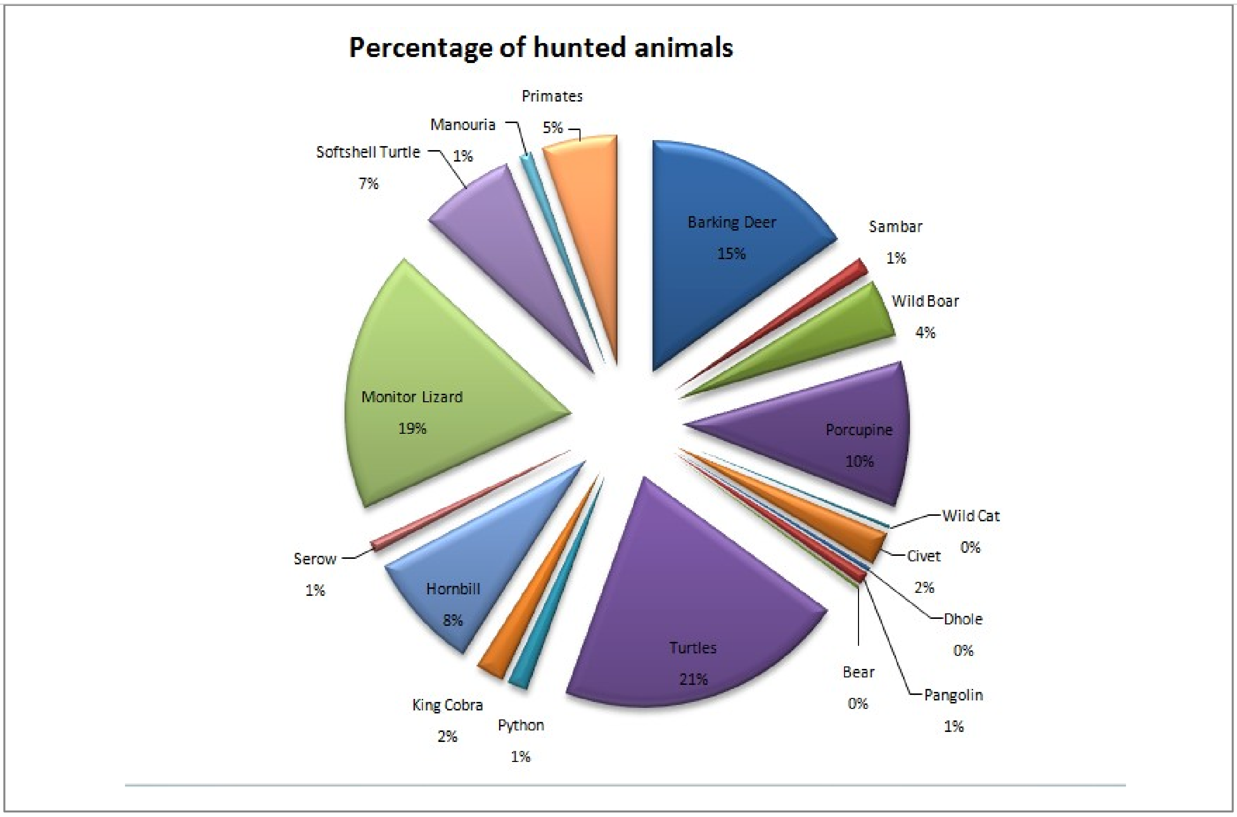

Figure 34: Percentage of hunted animals

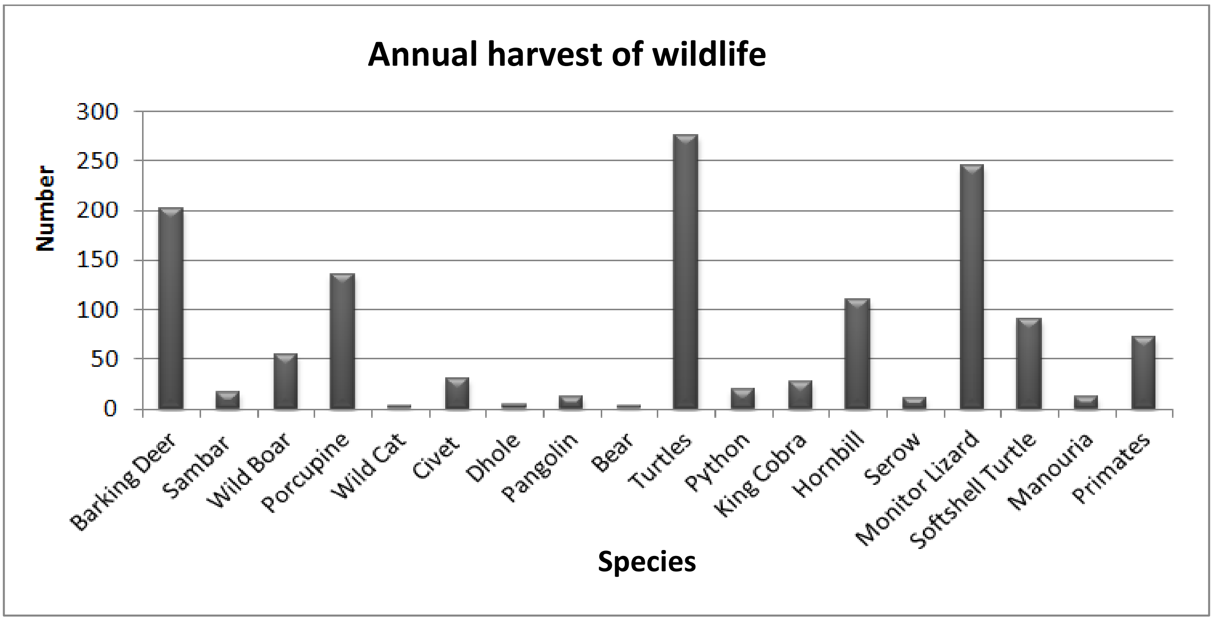

Figure 35: Number of hunted animals (survey data collected from 26 Mro villages)

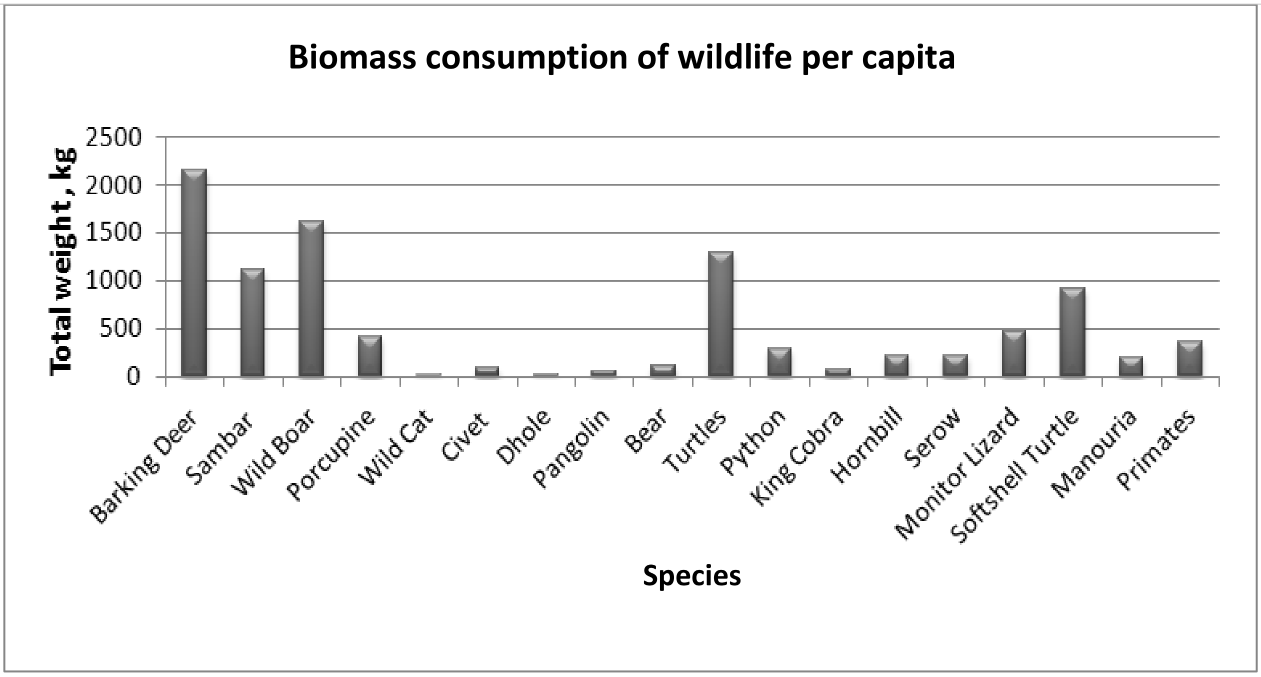

Figure 36 : Per capita biomass consumption for different taxa(survey data collected from 26 Mro villages)

However, if threats like hunting pressure and deforestation are not addressed properly and immediately, then in near future many of those least concern species will also be found in threatened or near threatened categories with few exceptions based on adaptability. For example, the Bengal monitor is a generalist predator and is highly adaptable, meaning it will probably readily survive with additional habitat changes and high hunting pressure. The great hornbill, in contrast, feeds on tree fruits and needs large old-growth trees with cavities in which to nest, so the clearing of new land for *jhum* agriculture is extremely detrimental to its survival. It may also be particularly sought by hunters due to its great size and its attractive black and white feathers.

Hunting of mammal species such as hog badger, ferret badger, and mongoose was avoided because they have an unpleasant taste, possibly due to their strong musk glands, and therefore do not have any consumption value. Villagers also do not hunt poisonous snakes such as the red-necked keelback (*Rhabdophis subminiatus*) which has a nuchal gland containing toxins, but they do hunt the highly venomous king cobra (*Ophiophagus hannah*) which is considered a delicacy. Monocled cobras (*Naja kaouthia*), rat snakes (*Ptyas* spp.,*Coelognathus* spp.), and keelbacks (*Xenochrophis* spp.) are taken when opportunistically encountered, while pit vipers (*Trimeresurus* spp.) and smaller snakes, such as mock vipers (*Psammodynastes pulverulentus*), wolf snakes (*Lycodon* spp.), and kukri snakes (*Oligodon* spp.) were avoided**.** Larger frogs (*Hoplobatrachus* spp., *Amalops* spp.) and tadpoles are usually consumed, but tree frogs (Family: Rhacophoridae) and other small frogs are generally

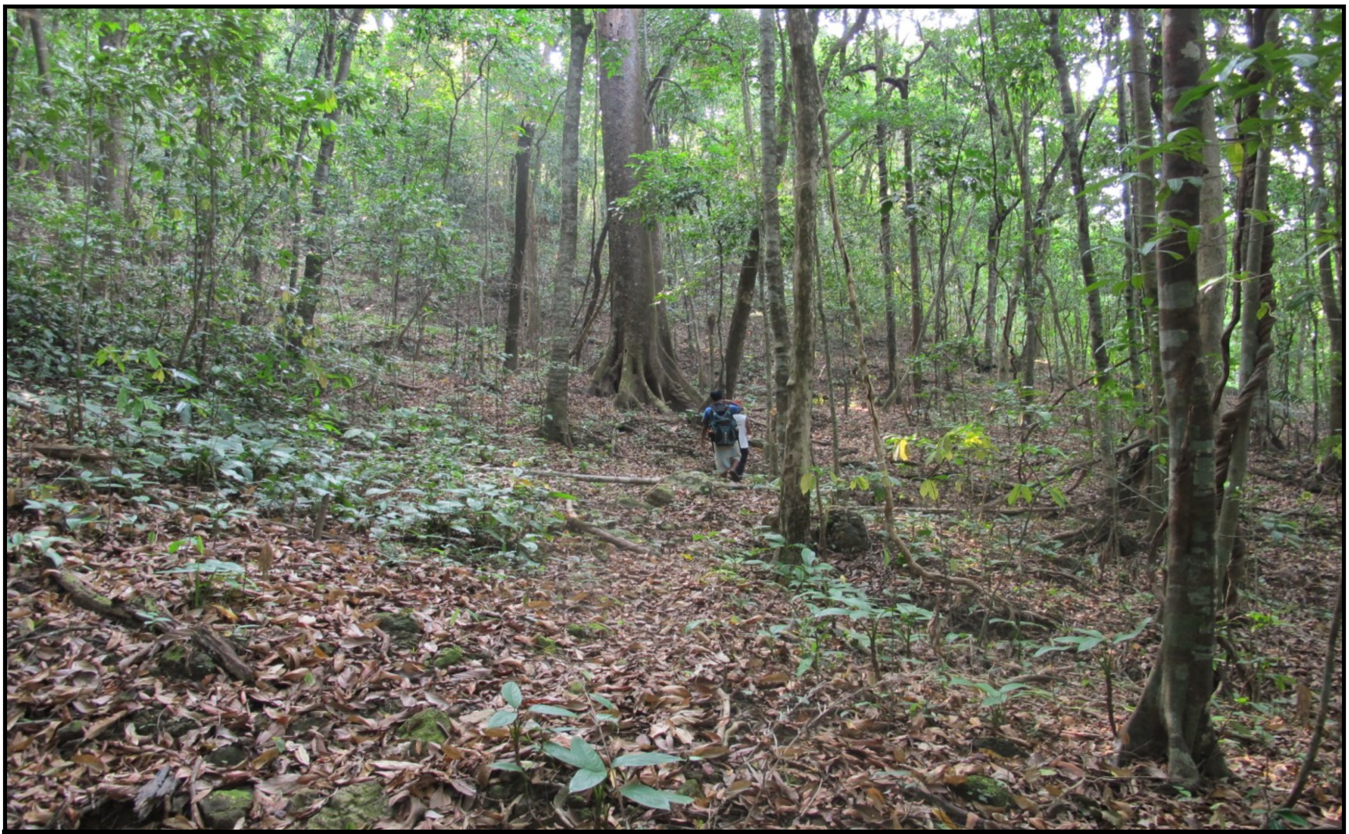

Figure 37: Landscape image of hill forest

avoided. Monitor lizards (*Varanus* spp.) are commonly eaten and considered a delicacy, while skinks (Family: Scincidae) and other lizards are not consumed.

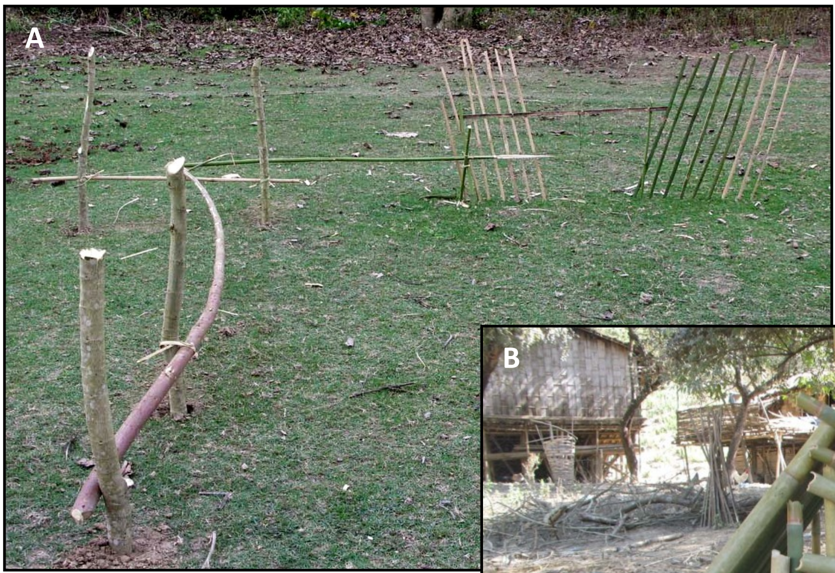

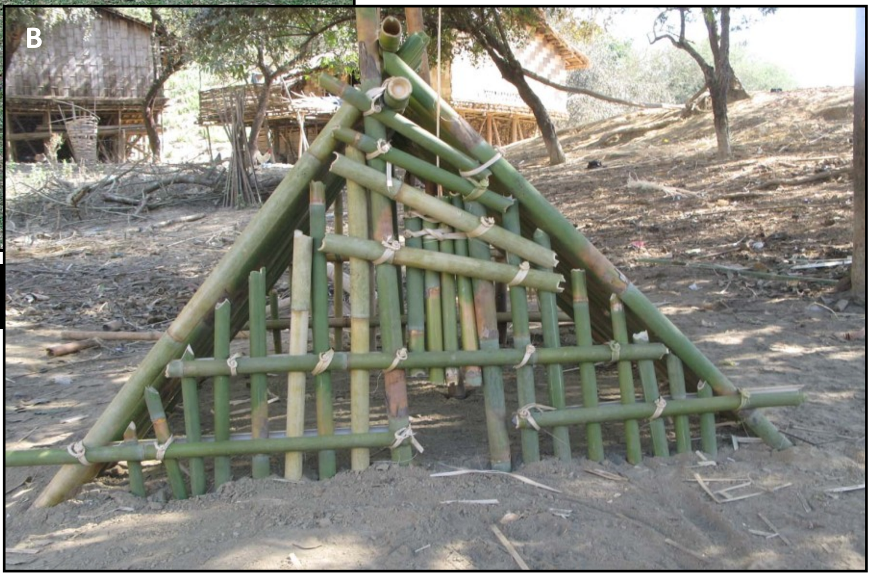

Figure 38: A) Shot; B) Pre-chek

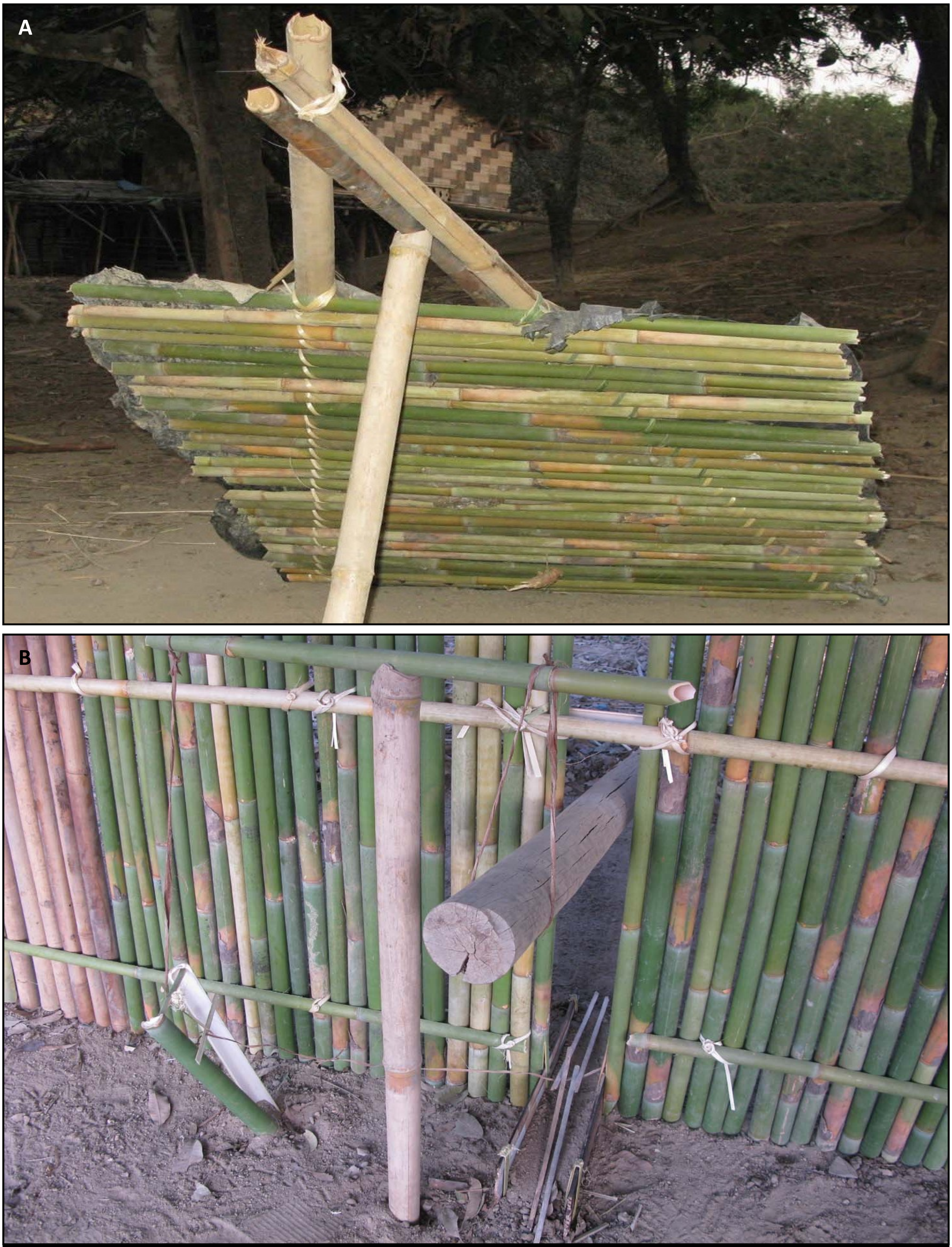

Fig.ure 39: A. Rip front view; B. Clong

Thirteen hunting techniques (Table 2) were considered passive approaches: snare traps, box traps, spear traps, pit fall traps, glue traps, dead fall traps and mist netting; the remaining were active methods: searching for animals, employing hand-made guns, hunting dogs, catapults, blow-darting, bow-and-arrow, and spears. Both forms of hunting took place within a 3 km radius of the village.

A simple correlation plot was created to see the correlation between distance of a village from the nearest town, length of fallow years, village population, number of hunter, number of hunters who use guns and traps, and the total number of animals hunted in a village.

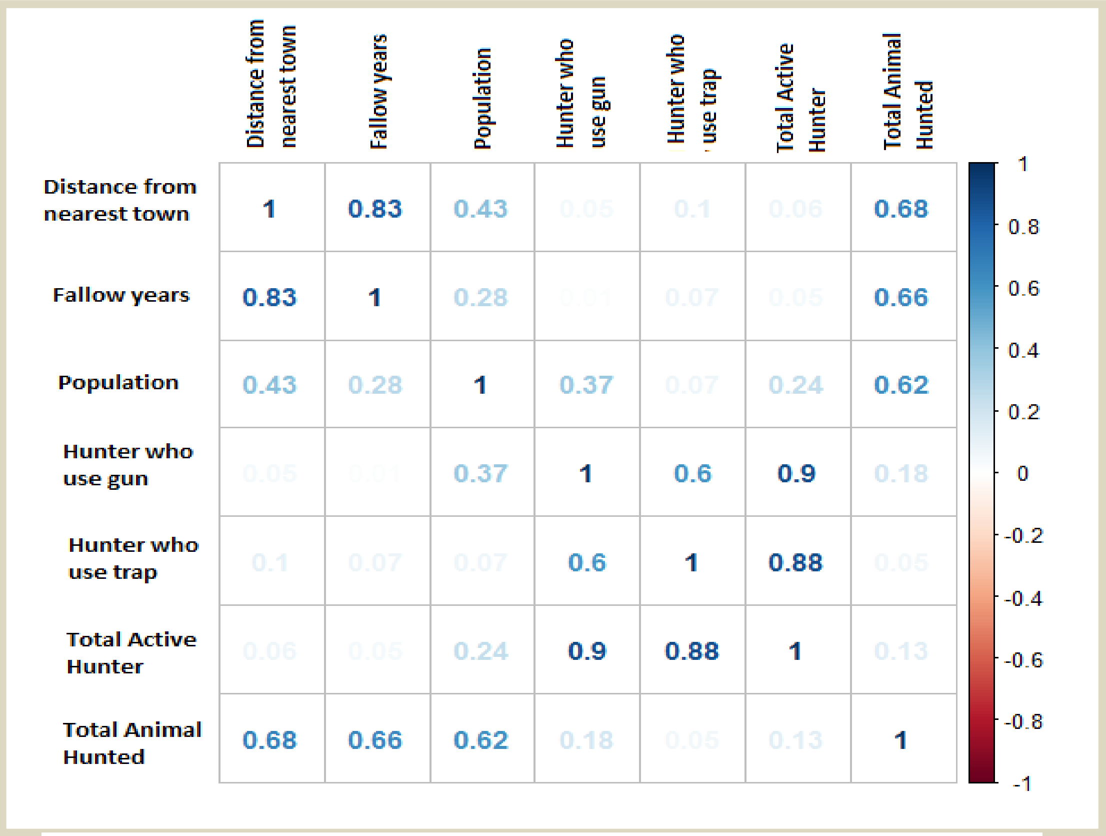

Figure 40 : Correlation matrix

There is a likelihood of underestimation of hunted species as the survey failed to account the passerine birds, bats, and small mammals which are widely hunted. These are usually not represented, or represented by only fragments, in any of the catch data we were able to obtain. Furthermore, even when intact, these are often difficult to identify to genus and species. The hill myna was an exception, as villages believe bad things will happen to them if they consume one, and thus recall is high. These birds are also highly sought after for the live animal trade and therefore have higher recall value.

Although exact quantification of the abundance of wildlife available was beyond the scope of this survey, the area’s remoteness allows for the existence of higher quality habitat for wildlife and thus a

presumably higher abundance of wildlife. Therefore, hunting offtake would be higher in more remote villages with better forest coverage. The continuation of subsistence hunting, combined with the destruction and alteration of primary forest patches for the swidden agriculture system, will likely cause depletion of many wildlife species, and, without immediate intervention will cause extirpation of already low populations of many globally threatened species, such as turtles, gaur, and primates

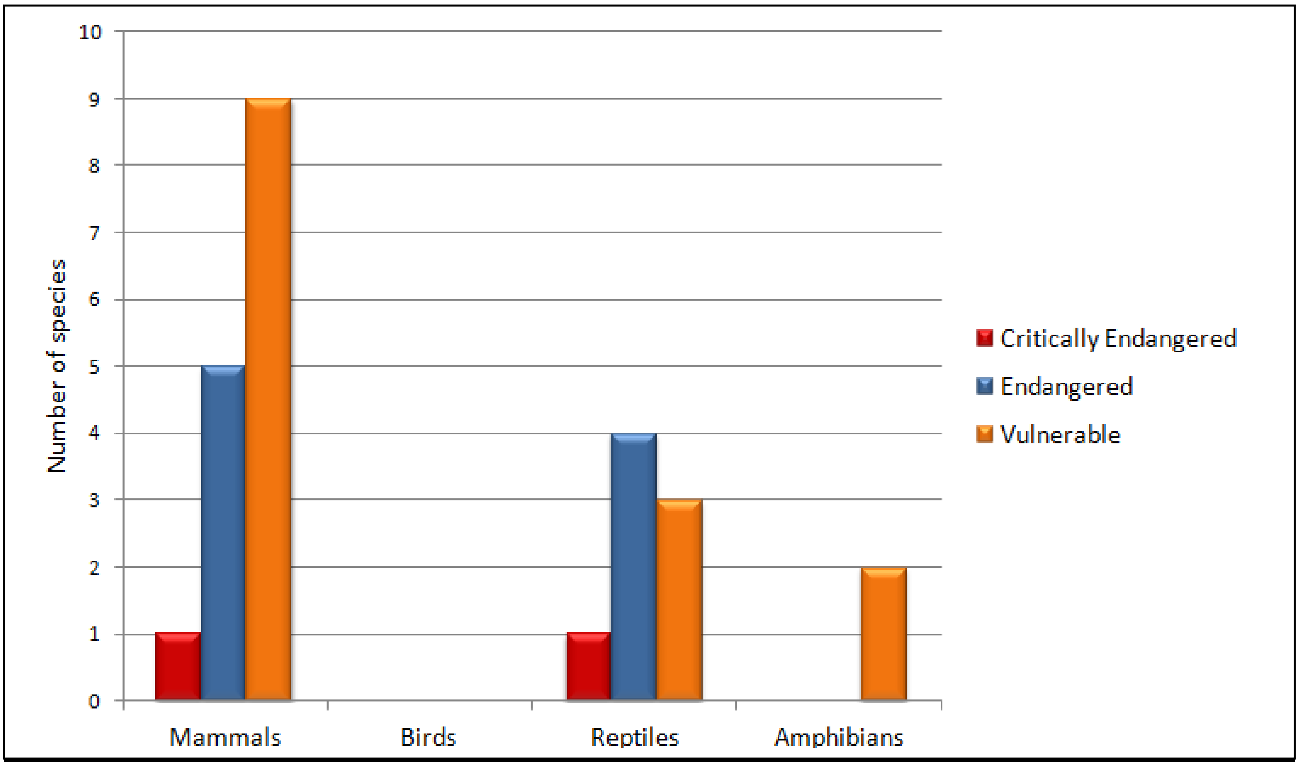

Figure 41: Taxa representing different Red List threatened categories

7. Threats to biological diversity

1. Human influx

Historically there were no human settlements in the SRF. Due to the decrease in soil fertility in adjacent areas, more and more people are moving into the SRF every year with hopes for better crop production. The establishment of new villages within the core area of the SRF in recent years is a serious threat for the biodiversity of the area.

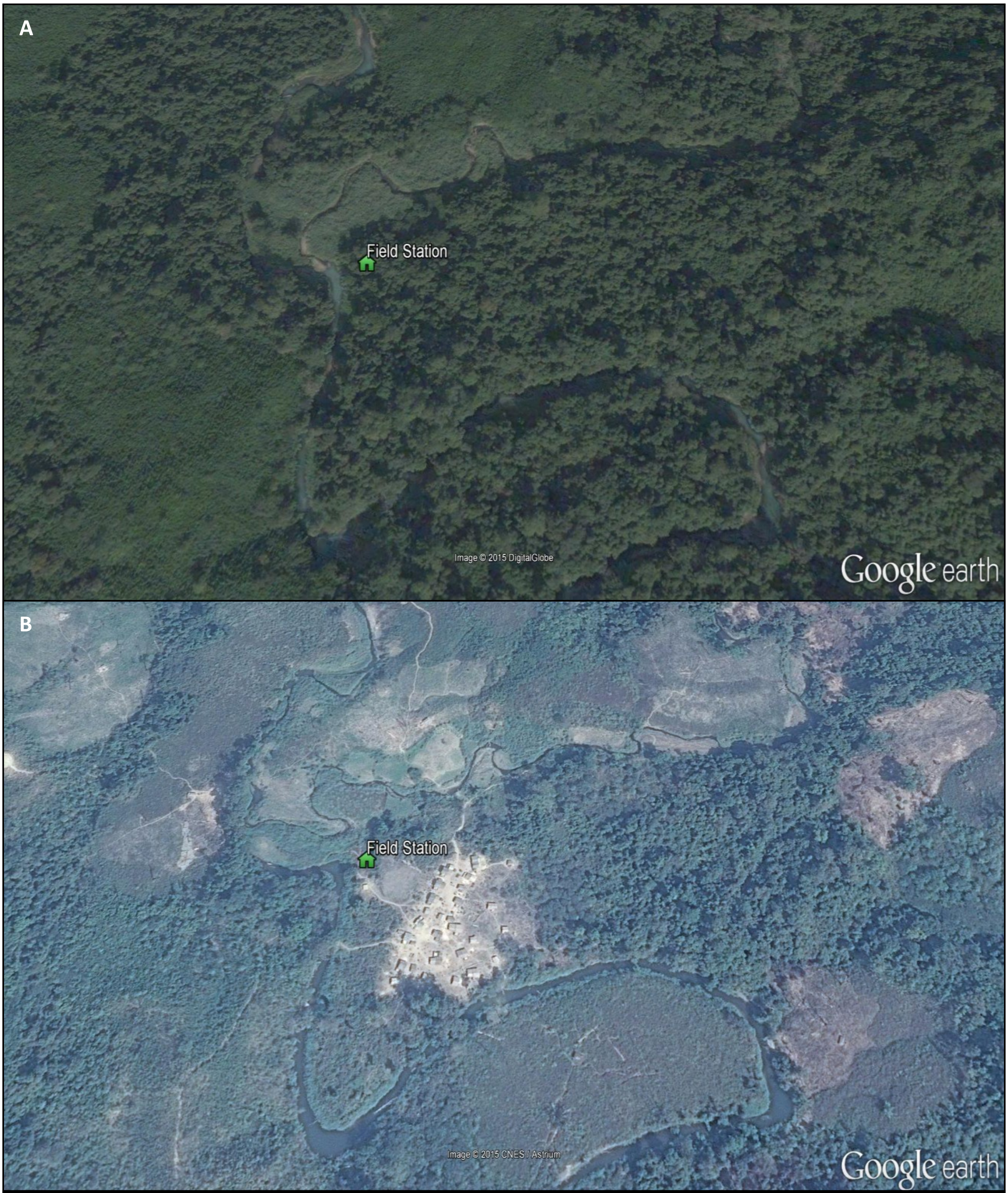

Figure 42 : Satellite image showing landscape change between 2002 (A) and 2015 (B) due to human

2. Jhum cultivation

Due to the increase in human population and rapid influx of people from other areas, jhum cultivation, without regulation, will likely cause the destruction of the remaining forest patches within the core of the SRF.

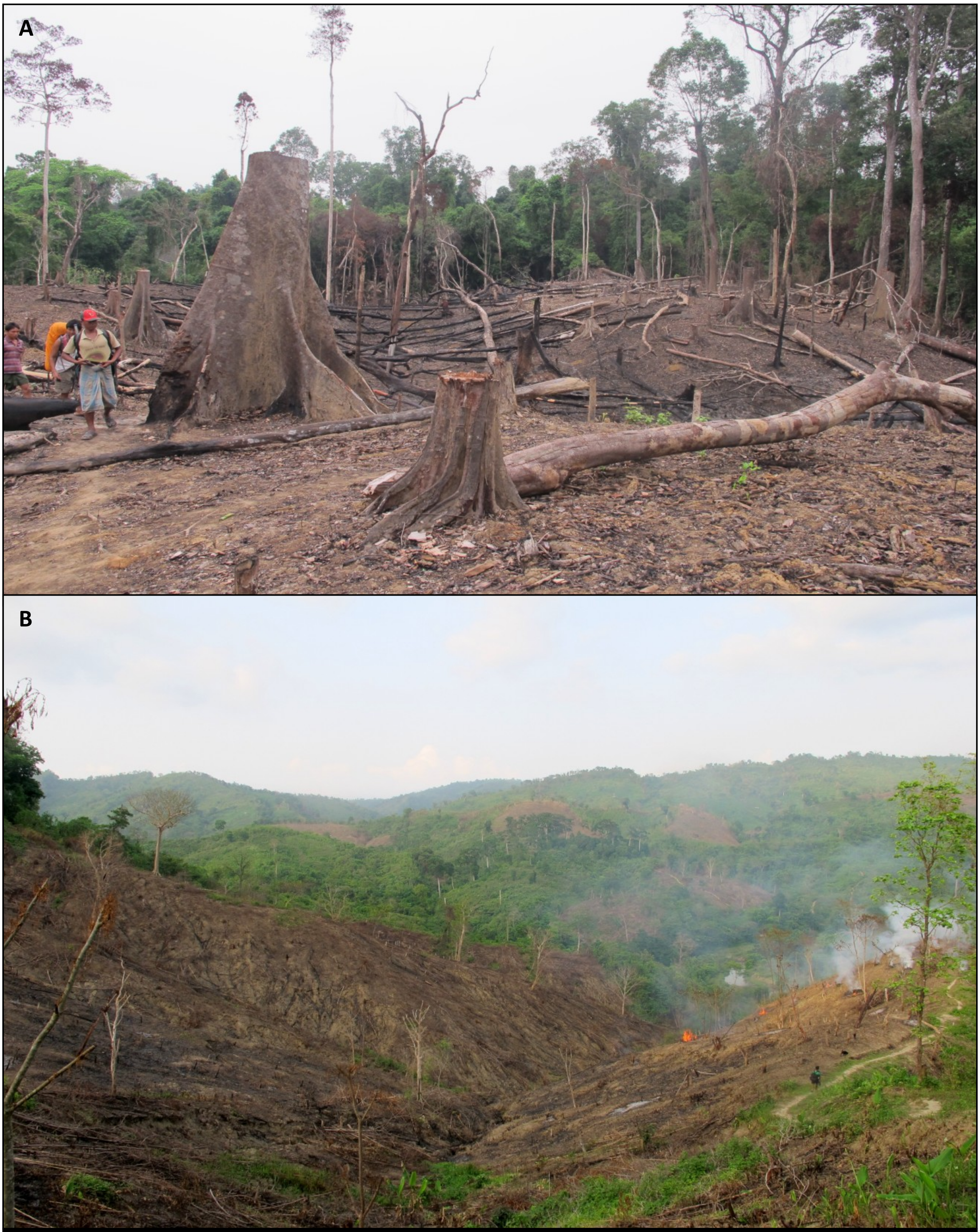

Figure 43: A. Cutting of old growth forest for jhum B. Slash and burn process for the cultivation of Jhum

3. Extraction of forest products by seasonal resource collectors

During the dry season (October to March), thousands of people, mostly of tribal communities, enter the SRF to collect rattan, bamboo, trees without any regulation. This practice is unsustainable and with the current rate of harvest continues it would cause serious degradation of the area.

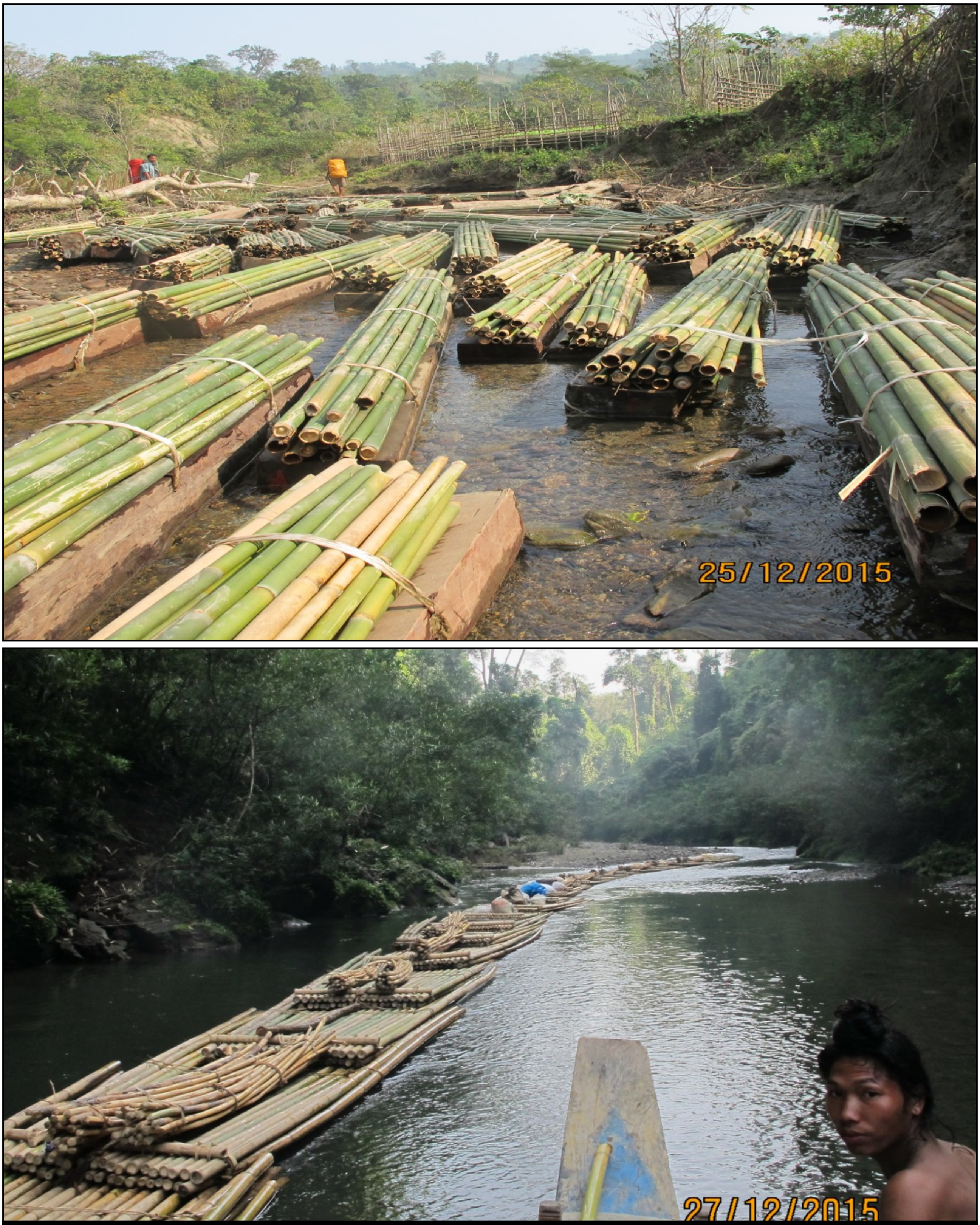

Figure 44: A. Logging in Matamuhuri Reserve Forest; B. Unsustainable harvest of bamboo and rattan during dry season from Sangu Reserve Forest

4. Logging

The selective logging in the remaining primary forest patches of SRF and MRF, primarily, by the Bengali people, is a serious threat for the biodiversity of the area.

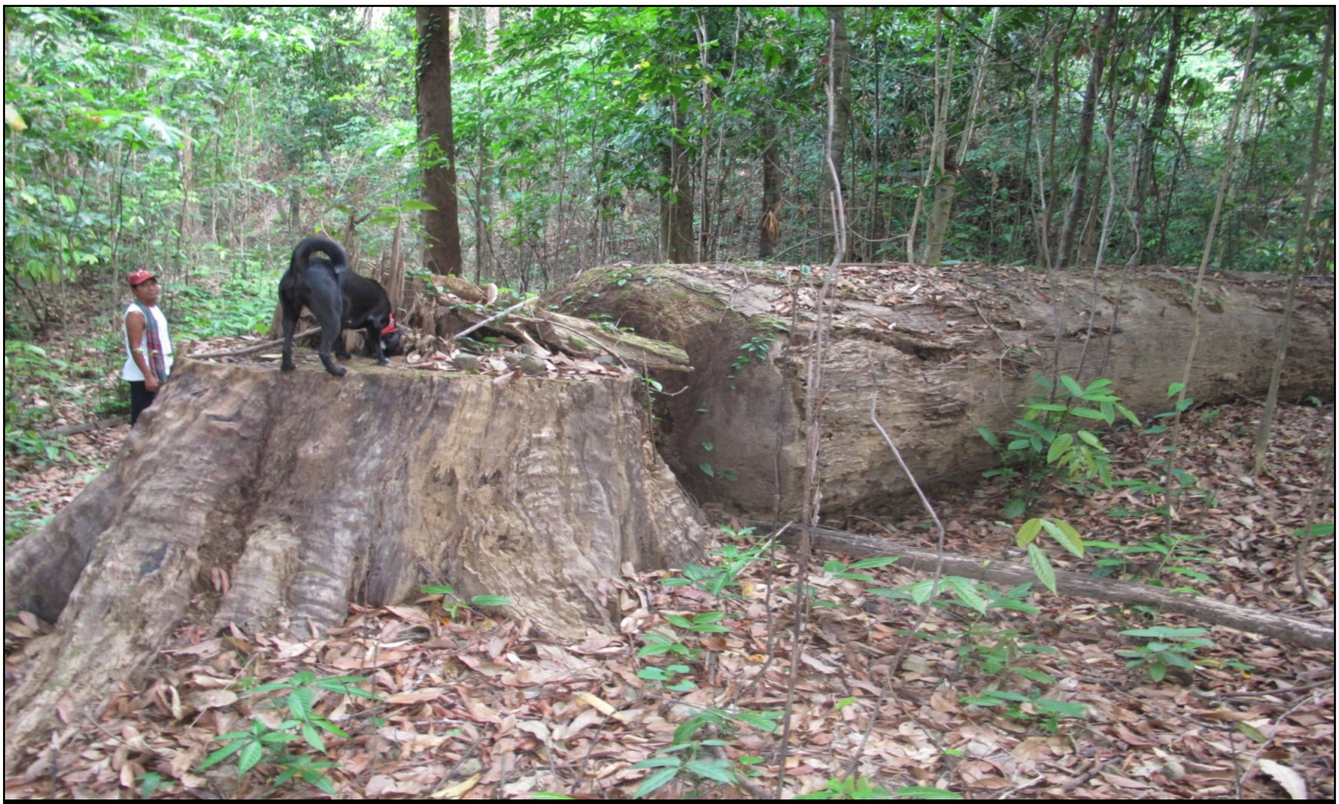
Figure 45: Selective Logging

5. Subsistence hunting and poaching

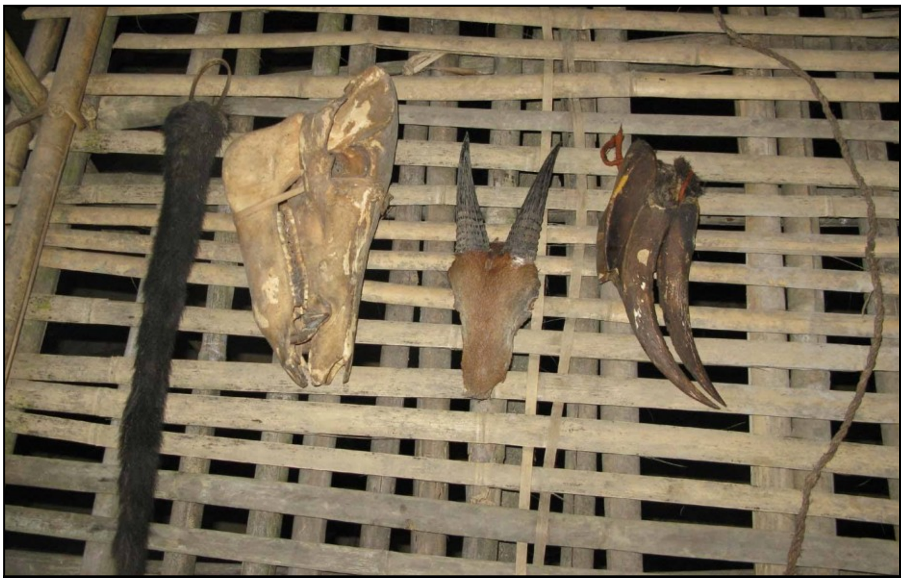
Figure 46: Hunting practice

The tribal communities in the SRF appeared to be largely composed of opportunistic, subsistence hunters. However, the wide spread, chronic, subsistence hunting practice can cause the local extinctions of many species and their prey. Trans-boundary hunting is also a threat in the area. Hunters from Myanmar would often visit the area for targeted species, such as pangolin, tiger, elephants, and other big cats.

8. WAY FORWARD?

During our initial reconnaissance in the Sangu Reserve Forest we managed to observe a wide array of wildlife, much of which can be seen nowhere else in the country. In spite of our surveys being preliminary and exploratory in nature, lacking rigorous scientific protocols, the amount of biodiversity that our tribal field assistants (parabiologists) managed to acquire with their handheld cameras and few of our camera traps, has far exceeded anyone's expectations. A number of nationally and globally threatened species of amphibians, reptiles, and mammals were photographed, many of which require immediate attention. Our data indicates that Sangu Reserve Forest is one of the most important forests of Bangladesh. The adjacent Matamuhuri Reserve Forest has been degraded substantially due to the encroachment by both ethnic and Bengali communities. Without intervention, the remaining treasure trove of biodiversity might be lost from Sangu Reserve Forest forever. Most importantly, this forest is part of the watershed of Bangladesh's two indigenous river-Sangu and Matamuhuri. A methodic and collaborative approach by the Forest Department, involving the local communities and other government stakeholders, is likely the only way we can protect and restore this forgotten gem to its former glory.

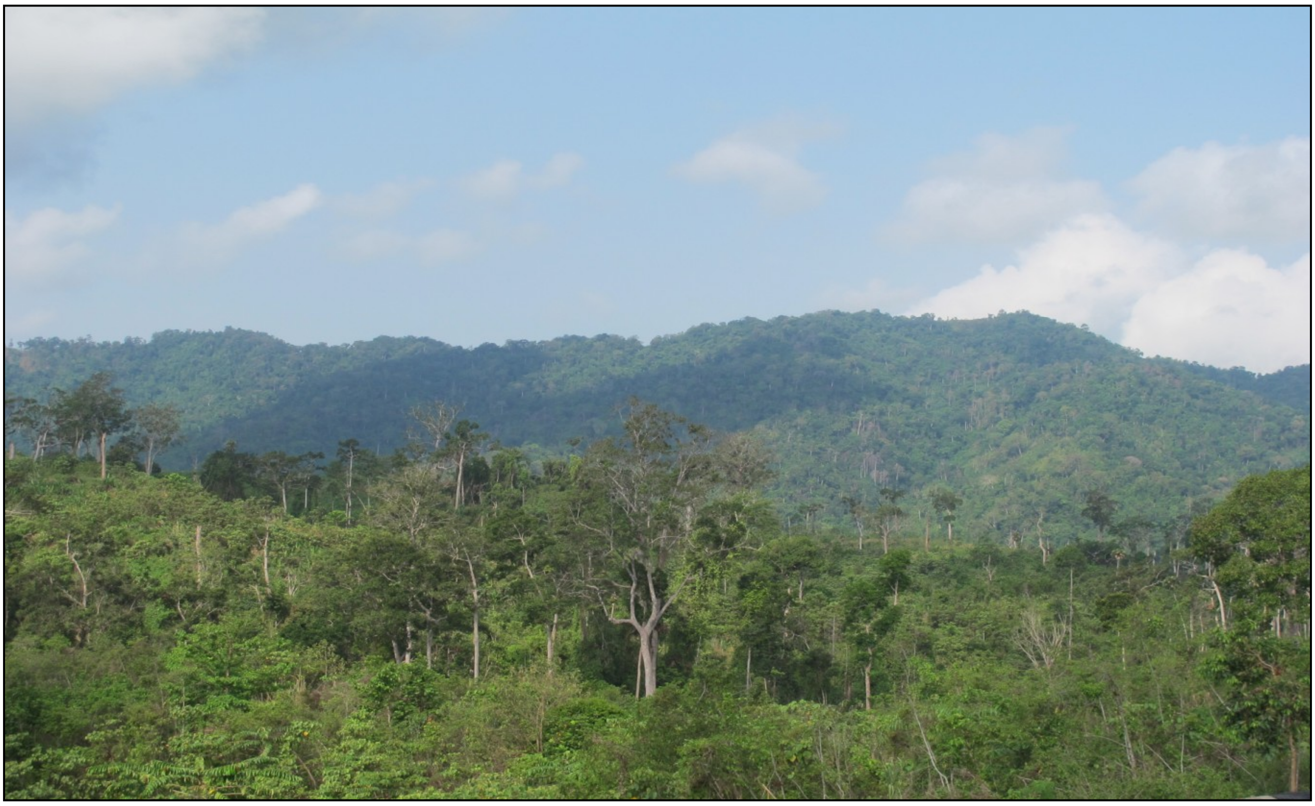

9. Appendix

Table 1: A preliminary checklist of wildlife of Sangu Reserve Forest, an adjacent areas, observed by our parabiologists (Only species with photographic evidence were included in this checklist).

Common Name	Scientific Name	IUCN Red List Classification	Primary Use
**Mammals (37)**			
**Asian Elephant**	*Elephas maximus*	Endangered	Meat
**Tiger**	*Panthera tigris*	Endangered	Wildlife trade
**Asiatic Golden Cat**	*Catopuma temminckii*	Near Threatened	Meat
**Leopard**	*Panthera pardus*	Least Concern	Meat
**Clouded Leopard**	*Neofelis nebulosa*	Vulnerable	Meat
**Leopard Cat**	*Prionailurus bengalensis*	Least Concern	Meat
**Marble Cat**	*Pardofelis marmorata*	Near Threatened	Meat
**Asiatic Black Bear**	*Ursus thibetanus*	Vulnerable	Meat
**Sun Bear**	*Helarctos malayanus*	Vulnerable	Meat
**Binturong**	*Arctictis binturong*	Vulnerable	Meat
**Gaur**	*Bos gaurus*	Vulnerable	Meat
**Civet sp.**	Family: Viverridae	Least Concern	Meat
**Large Indian Civet**	*Viverra zibetha*	Least Concern	Meat
**Masked Palm Civet**	*Paguma lavarta*	Least Concern	Meat
**Malayan Porcupine**	*Hystrix brachyura*	Least Concern	Meat
**Brush-tailed Porcupine**	*Atherurus macrourus*	Least Concern	Meat
**Red Serow**	*Capricornis sp.*	Near Threatened	Meat
**Sambar**	*Rusa unicolor*	Vulnerable	Meat
**Barking Deer**	*Muntiacus vaginalis*	Least Concern	Meat
**Wild Boar**	*Sus scrofa*	Least Concern	Meat
**Phayre's Leaf Monkey**	*Trachypithecus phayrei*	Endangered	Meat
**Bengal Slow Loris**	*Nycticebus bengalensis*	Vulnerable	Meat
**Capped Langur**	*Trachypithecus pileatus*	Vulnerable	Meat
**Northern Pig-tailed Monkey**	*Macaca leonina*	Data deficient	Meat
**Western Hoolock Gibbon**	*Hoolock hoolock*	Endangered	Meat

**Rhesus Macaque**	*Macaca mulata*	Least Concern	Meat
**Tree Shrew**	*Tupaia glis*	Least Concern	Meat
**Rats/Mice**	*Muridae spp.*	Least Concern	Meat
**Flying Fox**	*Pteropus giganteus*	Least Concern	Meat
**False Vampire**	*Megaderma sp.*	Data deficient	Meat
**Chinese Pangolin**	*Manis pentadactyla*	Critically Endangered	Wildlife trade
**Pallas' Squirrel**	*Callosciurus erythraeus*	Least Concern	Meat
**Malayan Giant Squirrel**	*Ratufa bicolor*	Near Threatened	Meat
**Flying squirrel**	*Petaurista sp.*	Least Concern	Meat
**Particolored Flying Squirrel**	*Hylopetes alboniger*	Least Concern	Meat
**Orange-bellied Himalayan Squirrel**	*Dremomys lokriah*	Least Concern	Meat
**Irrawady Squirrel**	*Callosciurus pygerythrus*	Least Concern	Meat
**Reptiles (46)**			
**Arakan Forest Turtle**	*Heosemys depressa*	Critically Endangered	Meat
**Sylhet Roofed Turtle**	*Pangshura sylhetensis*	Endangered	Meat
**Malayan Softshell Turtle**	*Amyda ornata*	Vulnerable	Meat
**Asian Leaf Turtle**	*Cyclemys gemeli*	Data Deficient	Meat
**Indian Flapshell Turtle**	*Lissemys punctata*	Least Concern	Meat
**Keeled Box Turtle**	*Cuora mouhotii*	Endangered	Meat
**Elongated Tortoise**	*Indotestudo elongata*	Endangered	Meat
**Asian Brown Tortoise**	*Manouria emys*	Endangered	Meat
**Reticulated Python**	*Python malayo*	Least concern	Meat
**Burmese Python**	*Python bivittatus*	Vulnerable	Meat
**King Cobra**	*Ophiophagus hannah*	Vulnerable	Meat
**Monocled Cobra**	*Naja kaouthia*	Least Concern	Meat
**Checkered Keelback**	*Xenochrophis piscator*	NA	Meat
**Yellow-spotted Keelback**	*Xenochrophis flavipunctatus*	Least Concern	Meat
**Blind Snake**	*Typhlops diardi*	Least Concern	Not Preferred
**Brahminy Blind Snake**	*Indotyphlops braminus*	Least Concern	Not Preferred
**Short-nosed Vine Snake**	*Ahaetulla prasina*	Least Concern	Not Preferred
**Cherapunjee Keelback**	*Amphiesma xenura*	Least Concern	Not Preferred

**Venning's Keelback**	*Amphiesma venningi*	Least Concern	Not Preferred
**Green Cat Snake**	*Boiga cyanea*	Least Concern	Not Preferred
**Tawny Cat Snake**	*Boiga ochracea*	Least Concern	Not Preferred
**Zawi Wolf Snake**	*Lycodon Zawi*	Least Concern	Not Preferred
**Kukri Snake**	*Oligodon albocinctus*	NA	Not Preferred
**Kukri Snake**	*Oildogodon spp.*	NA	Not Preferred
**Indochinese Rat Snake**	*Ptyas korros*	NA	Meat
**Mock Viper**	*Psammodynastes pulverulentus*	NA	Not Preferred
**Red-necked Keelback**	*Rhabdophis subminatus*	Least Concern	Not Preferred
**Trinket Snake**	*Coelognathus radiatus*	Least Concern	Meat
**Forest Snake**	*Rhabdops sp.*	NA	Not Preferred
**Assam Snail Eater**	*Pareas monticola*	NA	Not Preferred
**Black Krait**	*Bungarus niger*	NA	Not Preferred
**Banded Krait**	*Bungarus fasciatus*	Least Concern	Not Preferred
**Reed Snake**	*Calamaria spp.*	NA	Not Preferred
**Bengal Monitor**	*Varanus bengalensis*	Least Concern	Meat
**Spiny-headed Forest Lizard**	*Calotes emma*	NA	Meat
**Indian Garden Lizard**	*Calotes versicolor*	NA	Meat
**Blue-throated Lizard**	*Ptyctolameus gularis*	NA	Meat
**Tokay Gecko**	*Gekko gekko*	Least Concern	Meat
**Bent-toed Gecko**	*Cyrtodactylus spp.*	NA	Meat
**House Gecko**	*Hemidactylus spp.*	NA	Meat
**Long Tailed Lizard**	*Takydromus spp.*	NA	Meat
**Many-lined Grass Skink**	*Eutrophis multifasciata*	NA	Meat
**Bronze Grass Skink**	*Eutrophis macaluria*	NA	Meat
**Spotted Litter Skink**	*Spenomorphus spp.*	NA	Meat
**Water Skink**	*Tropidophorous assamensis*	NA	Meat
**Amphibians (19)**			
**Caecilian**	*Ichthyophis sp.*	Least Concern	Not Preferred
**Cascade Frog**	*Amalops sp.*	NA	Meat
**Indian Bull frog**	*Hoplabatrachus tigrinus*	Least Concern	Meat

**Jerdon's Bull Frog**	*Hoplabatrachus crassus*	Least Concern	Meat
**Common Asian Toad**	*Duttaphrynys melanostictus*	Least Concern	Meat
**Mountain Horned Frog**	*Xenphrys parva*	Least Concern	Not preferred
**Painted Baloon Frog**	*Kaloula pulchra*	Least Concern	Not preferred
**Ornamented Pigmy Frog**	*Microhyla ornata*	Least Concern	Not preferred
**Two-striped Tree Frog**	*Chiromantis viattatus*	Least Concern	Not preferred
**Unidentified Frog**	*Microhyla sp.*	NA	Not preferred
**Cricket Frog**	*Fejarvarya spp.*	NA	Not preferred
**Red-eyed Frog**	*Leptobrachium smithii*	Least Concern	Not preferred
**Rivulet Frog**	*Limnonectes laticeps*	Least Concern	Not preferred
**Not preferred**	*Ingerana borealis*	Vulnerable	Not preferred
**Cascade Frog**	*Amalops spp.*	NA	Meat
**Assam Forest Frog**	*Sylvarina leptoglossa*	Least Concern	Meat
**Bush Frog**	*Philatus sp.*	NA	Not preferred
**Tree Frog**	*Polypedates spp.*	NA	Not preferred
**Indian Flying Frog**	*Pterorana khare*	Vulnerable	Not preferred
**Birds (11)**			
**Great Hornbill**	*Buceros bicornis*	Near Threatened	Meat
**Wreathed Hornbill**	*Rhyticeros undulatus*	Least Concern	Meat
**Oriental-pied Hornbill**	*Anthracoceros albirostris*	Least Concern	Meat
**Pigeons/Doves**	*Columbidae spp.*	Least Concern	Wildlife trade
**Racket-tailed Drongo**	*Dicrurus sp.*	Least Concern	Wildlife trade
**Hill Myna**	*Gracula religiosa*	Least Concern	Wildlife trade
**Red Jungle Fowl**	*Gallus gallus*	Least Concern	Meat
**Kalij Pheasant**	*Lophura leucomelanos*	Least Concern	Meat
**Vernal Hanging Parrot**	*Loriculus vernalis*	Least Concern	Wildlife trade
**Grey-Peacock Pheasant**	*Polyplectron bicalcaratum*	Least Concern	Meat
**Parakeets**	*Psittacula sp.*	Least Concern	Wildlife trade

Table 2. List of hunting techniques and methods used by the Mro

No.	Traps	Local name	Trap location	Materials used	Target Species	Seasonal Use
Passive trapping methods						
1	Stone/mud dead fall	*Rip*	Ground; Near stream	Mud slab, bamboo	Baited; civet; birds	Winter
2	Trigger and release spear trap	*Shot*	Ground	Bamboo, cane	Wild dog, wild boar, wild cats, porcupine, deer, medium-size mammals	Monsoon near jhum field; winter
3	Arboreal snare trap	*Dong*	Trees	Rope, bamboo	Squirrels; Rats	Monsoon mostly; and winter
4	Jungle Foul Snare Trap	*Oram-Pow*	On Ground	Rope snare; baited with trained domesticated chicken	Jungle fowl	Summer
5	Neck-hold snare trap	*Nong-Lnag*	Ground	Rope, bamboo	Porcupines, civets; small-medium size mammals; jungle fowl	Monsoon near jhum fields; less in winter and summer
6	Gum trap	*Nye*	Trees	Resin, Insect, bamboo pole	Insectivorous birds	Winter
7	Bird Snare Trap	*Ober-Lang*	Trees	Rope baited with fruits	Frugivorous birds	Winter;
8	Leg-hold snare trap	*Khog-Lang*	Ground	Rope, tree pole	Deer, wild boar, sambar, wild cats, large mammals	Monsoon and Winter
9	Fixed Bamboo-spear trap	*Chaw-ow*	Ground	Bamboo spear	Deer, wild boar; medium to large mammals	Fall and winter
10	Pit fall trap	*Wam*	Ground	Pit fall	Terrestrial turtles	Monsoon
11	Box trap	*Pre-check*	Ground	Bamboo	For live trapping nuisance animals	Year round; Monsoon
12	Log fall	*Clong*	Ground	Large log, bamboo	Snakes; jungle fowl; rats	Monsoon and fall;
13	Mist Net	*Pauk*	Ground	Nets	Birds, bats	Winter;
Active trapping methods						
14	Gun	*Napauk*	-	-	Birds, monkeys, deer, wild boar	Winter
15	Catapult	*Yaiba*	-	-	Birds, squirrels	Year round; Winter
16	Blow Dart	*Muklong*	-	-	Birds	Winter
17	Hunting Dog	*Kui-shuk*	-	-	Terrestrial turtles, monitor lizards, wild boar, deer	Monsoon;
18	Spear	*Longachu*	-	-	Soft shell turtles *(Amydaornata)*	Year round; Winter

10. References

Aiyadurai, A., Singh, N. J., & Milner-Gulland, E. J. (2010). Wildlife hunting by indigenous tribes: a case study from Arunachal Pradesh, north-east India.*Oryx*, *44*(04), 564-572.

Aziz, S. A., Clements, G. R., Rayan, D. M., & Sankar, P. (2013). Why conservationists should be concerned about natural resource legislation affecting indigenous peoples' rights: lessons from Peninsular Malaysia.*Biodiversity and conservation*, *22*(3), 639-656.

Bonham, C. A., Sacayon, E. &Tzi, E. (2008). Protecting imperilled paper parks: potential lessons from the Sierra Chinajá, Guatemala. Biodiversity and Conservation, 17, 1581–1593.

Corlett, R. T. (2007). The impact of hunting on the mammalian fauna of tropical Asian forests. *Biotropica*, *39*(3), 292-303.

Cox, P. A., & Elmqvist, T. (1991). Indigenous control of tropical rain-forest reserves: an alternative strategy for conservation. *Ambio*, 317-321.

Doughty, H. L., Karpanty, S. M., & Wilbur, H. M. (2015). Local hunting of carnivores in forested Africa: a meta-analysis. *Oryx*, *49*(01), 88-95.

Dove, M. R. (2006). Indigenous people and environmental politics. *Annu. Rev. Anthropol.*, *35*, 191-208.

Drury, R. (2009). Reducing urban demand for wild animals in Vietnam: examining the potential of wildlife farming as a conservation tool. *Conservation Letters*, *2*(6), 263-270.

Fa, J. E., & Brown, D. (2009). Impacts of hunting on mammals in African tropical moist forests: a review and synthesis. *Mammal Review*, *39*(4), 231-264.=
Fox, S. R. (1981). *The American conservation movement: John Muir and his legacy*. Univ of Wisconsin Press.

Gubbi, S., &Linkie, M. (2012). Wildlife hunting patterns, techniques, and profile of hunters in and around Periyar Tiger Reserve. *Journal of the Bombay Natural History Society*, *109*(3), 165-172.

Islam, M.A., M. Uddin , M.A. Aziz , et al. 2013. Status of bears in Bangladesh: going, going, gone? Ursus, 24, 83–90.

Khan, M.M.H. 2015. *Chittagong Hill Tracts-The Land of Diversity,* Bangladesh Forest Department, Dhaka, Bangladesh. 167 pp.

Kramer, R., Schaik, C. V., & Johnson, J. (1997). *Last stand: protected areas and the defence of tropical biodiversity*. Oxford University Press.

Horowitz, L. S. (1998). Integrating indigenous resource management with wildlife conservation: a case study of Batang Ai National Park, Sarawak, Malaysia. *Human Ecology*, *26*(3), 371-403.

Lee, R. J. (2000). Impact of subsistence hunting in North Sulawesi, Indonesia, and conservation options. *Hunting for sustainability in tropical forests*, 455-472.

Luskin, M. S., Christina, E. D., Kelley, L. C., & Potts, M. D. (2014). Modern hunting practices and wild meat trade in the oil palm plantation-dominated landscapes of Sumatra, Indonesia. *Human Ecology*, *42*(1), 35-45.

Ling, S., & Milner-Gulland, E. J. (2006). Assessment of the sustainability of bushmeat hunting based on dynamic bioeconomic models. *Conservation Biology*, *20*(4), 1294-1299.

Milner-Gulland, E. J., & Bennett, E. L. (2003). Wild meat: the bigger picture.*Trends in Ecology & Evolution*, *18*(7), 351-357.

Mlambo, M. C. (2012). The urgent need for human well-being elements in biodiversity research. *Biodiversity and Conservation*, *21*(4), 1149-1151.

Myers, N., Mittermeier, R. A., Mittermeier, C. G., Da Fonseca, G. A., & Kent, J. (2000). Biodiversity hotspots for conservation priorities. *Nature*, *403*(6772), 853-858.

Naughton-Treves, L., Mena, J. L., Treves, A., Alvarez, N., &Radeloff, V. C. (2003). Wildlife Survival Beyond Park Boundaries: the Impact of Slash-and-Burn Agriculture and Hunting on Mammals in Tambopata, Peru. *Conservation Biology*, *17*(4), 1106-1117.

O'Brien, T. G., & Kinnaird, M. F. (2000). Differential vulnerability of large birds and mammals to hunting in North Sulawesi, Indonesia, and the outlook for the future. *Hunting for sustainability in tropical forests*, 199-213.

Peterson, J. T. (1981). Game, farming, and interethnic relations in northeastern Luzon, Philippines. *Human Ecology*, *9*(1), 1-22.

Quinten, M., Stirling, F., Schwarze, S., Dinata, Y., & Hodges, K. (2014). Knowledge, attitudes and practices of local people on Siberut Island (West-Sumatra, Indonesia) towards primate hunting and conservation. *Journal of Threatened Taxa*, *6*(11), 6389-6398.

Rao, M., Myint, T., Zaw, T., &Htun, S. (2005). Hunting patterns in tropical forests adjoining the Hkakaborazi National Park, north Myanmar. *Oryx*, *39*(03), 292-300.

Roy, R. D. (2002). Land and forest rights in the Chittagong Hill Tracts. *Talking Points*, *4*(02).

Robinson, J., and K. Redford. 1994. Measuring the sustainability of hunting in tropical forests. Oryx 28:249–256.

Robinson, J. G., & Bennett, E. L. (2004). Having your wildlife and eating it too: an analysis of hunting sustainability across tropical ecosystems. *Animal Conservation*, *7*(4), 397-408.

Scheffers, B. R., Corlett, R. T., Diesmos, A., &Laurance, W. F. (2012). Local demand drives a bushmeat industry in a Philippine forest preserve. *Tropical Conservation Science*, *5*, 133-141.

Schuster, S., &Schüle, W. (2000). Anthropogenic Causes, Mechanisms and Effects of Upper Pliocene and Quaternary Extinctions of Large Vertebrates.*Oxford journal of archaeology*, *19*(3), 223-239.

Sodhi N.S., Brook B.W. & Bradshaw C.A.J. 2009. Causes and consequences of species extinctions. In: Princeton Guide to Ecology (Levin S.A., editor). Princeton University Press. pp. 514-520.

Usher, P.J. & Wenzel, G. (1987) Native harvest surveys and statistics:a critique of their construction and use. Arctic, 40, 145–160.

Wadley, R. L., Colfer, C. J. P., & Hood, I. G. (1997). Hunting primates and managing forests: the case of Iban forest farmers in Indonesian Borneo.*Human Ecology*, *25*(2), 243-271.

West, P., Igoe, J., & Brockington, D. (2006). Parks and peoples: the social impact of protected areas. *Annu. Rev. Anthropol.*, *35*, 251-277.

Velho, N., &Laurance, W. F. (2013). Hunting practices of an Indo-Tibetan Buddhist tribe in Arunachal Pradesh, north-east India. *Oryx*, *47*(03), 389-392.

Yu, D. W., Levi, T., & Shepard, G. H. (2010). Conservation in Low-Governance Environments. *Biotropica*, *42*(5), 569-571.
